# Dual-Mode Tumor Imaging Using Probes That Are Responsive to Hypoxia-Induced Pathological Conditions

**DOI:** 10.3390/bios12070478

**Published:** 2022-06-30

**Authors:** S. A. Amali S. Subasinghe, Robia G. Pautler, Md. Abul Hassan Samee, Jason T. Yustein, Matthew J. Allen

**Affiliations:** 1 Department of Chemistry, Wayne State University, 5101 Cass Avenue, Detroit, MI 48202, USA; gb4621@wayne.edu; 2 Department of Integrative Physiology, Baylor College of Medicine, Houston, TX 77030, USA; rpautler@bcm.edu (R.G.P.); md.abulhassan.samee@bcm.edu (M.A.H.S.); 3 Integrative Molecular and Biomedical Sciences and the Department of Pediatrics in the Texas Children’s Cancer and Hematology Centers and The Faris D. Virani Ewing Sarcoma Center, Baylor College of Medicine, Houston, TX 77030, USA; yustein@bcm.edu

**Keywords:** dual-mode imaging, hypoxia, molecular imaging, tumor microenvironment

## Abstract

Hypoxia in solid tumors is associated with poor prognosis, increased aggressiveness, and strong resistance to therapeutics, making accurate monitoring of hypoxia important. Several imaging modalities have been used to study hypoxia, but each modality has inherent limitations. The use of a second modality can compensate for the limitations and validate the results of any single imaging modality. In this review, we describe dual-mode imaging systems for the detection of hypoxia that have been reported since the start of the 21st century. First, we provide a brief overview of the hallmarks of hypoxia used for imaging and the imaging modalities used to detect hypoxia, including optical imaging, ultrasound imaging, photoacoustic imaging, single-photon emission tomography, X-ray computed tomography, positron emission tomography, Cerenkov radiation energy transfer imaging, magnetic resonance imaging, electron paramagnetic resonance imaging, magnetic particle imaging, and surface-enhanced Raman spectroscopy, and mass spectrometric imaging. These overviews are followed by examples of hypoxia-relevant imaging using a mixture of probes for complementary single-mode imaging techniques. Then, we describe dual-mode molecular switches that are responsive in multiple imaging modalities to at least one hypoxia-induced pathological change. Finally, we offer future perspectives toward dual-mode imaging of hypoxia and hypoxia-induced pathophysiological changes in tumor microenvironments.

## 1. Introduction

**Figure 1:**
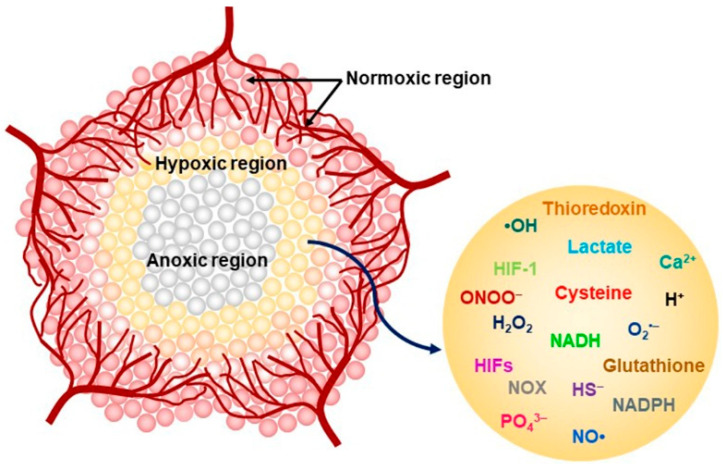
Schematic representation of regions of a tumor (**left**) and chemicals that are overproduced in hypoxic regions (**right**).

Hypoxia occurs as a consequence of inadequate delivery of oxygen to living cells [1,2], and it is a prognostic biomarker of several diseases including heart arrhythmia [3], Alzheimer’s disease [4,5], and cancer [6,7,8,9]. With cancer, hypoxia plays a critical role in solid tumors that possess rapidly proliferating abnormal cells with fast metabolic rates. Oxidative stress is the imbalance between the production and detoxification of reactive oxygen species that occurs as one of the major consequences of hypoxia [10]. As tumors expand, the distance between cells and blood vessels increases, and when tumors outgrow blood supply, the demand for oxygen is not satisfied by oxygen supply. Cells located roughly 150 μm beyond functioning blood vessels usual become necrotic due to lack of oxygen [11], and three distinct regions are observed in most solid tumors: normoxic (rapidly proliferating viable cells located close to blood vessels, pO_2_ > 2%), hypoxic (viable cells that are capable of surviving at a low oxygen concentration, pO_2_ ≤ 2%), and anoxic (mostly composed of dead cells without oxygen) [12]. To adapt to a dwindling supply of oxygen and nutrients, hypoxic cells activate a family of transcription factors, called hypoxia-inducible factors (HIFs) [13,14]. These factors, particularly hypoxia-inducible factor-1 (HIF-1), result in unique heterogenous tumor microenvironments compared to non-hypoxic cells as a result of altering the metabolic activity of hypoxic cells [15]. Tumor microenvironments have characteristic pathophysiological parameters, including low extracellular pH (<7.4) [16,17]; elevated levels of reactive oxygen [18,19], nitrogen [20,21], and sulfur [22,23] species; redox imbalance [24,25]; and accumulation of some inorganic ions (Figure 1) [26]. Moreover, these changes stabilize HIFs, further promoting tumor growth and eventually increasing resistance to radiotherapy and chemotherapy [27,28]. Therefore, the monitoring of hypoxia and oxidative stress is a critical endeavor.

Tumor hypoxia can be measured using O_2_ needle electrodes or imaging methods [29,30,31,32]. Needle electrodes are invasive and are limited in accuracy based on their placement in the tumor—they do not give a full tumor profile. Noninvasive detection of hypoxic tumors that are located deep within tissues is possible only with imaging methods, but each imaging method is limited by properties inherent to the method. For example, fluorescence imaging is highly sensitive, but due to light absorption and scattering, this modality has limited depth penetration [33].

Individual imaging modalities can be improved with respect to signal enhancement and specificity through the use of probes or contrast agents, and an emerging field of biomedicine involves the use of responsive probes and contrast agents with imaging modalities to characterize tumors [34,35,36]. These exogenous molecules often change their structure and chemical properties in response to hypoxia or hypoxia-induced changes in tumor microenvironments. Responsive imaging probes are studied with imaging modalities including positron emission tomography (PET), single-photon emission tomography (SPECT), and X-ray computed tomography (X-ray CT) because these imaging modalities possess nearly unlimited depth penetration [37], despite involving ionizing radiation that could damage cells [38,39]. Responsive contrast agents are also studied with magnetic resonance imaging (MRI) and ultrasound imaging to characterize hypoxic environments without the use of ionizing radiation. It is important to study each of these modalities because the orthogonal advantages and limitations of the modalities make them useful in different environments, and combinations of imaging modalities with distinct properties can overcome the limitations of any single modality, including the limitation of concentration-dependent probe response [40].

In this review, we describe dual-mode imaging methods reported for the ability to characterize hypoxia-relevant characteristics reported since the year 2000. For reviews of single-mode detection of hypoxia, readers are referred elsewhere [41,42,43,44,45,46,47]. This article is divided into two introductory sections—characteristics of oxidative stress and hypoxia that can be imaged and imaging modalities used to study hypoxia—followed by descriptions of dual-modality imaging of hypoxia using cocktails of probes in combination with multiple imaging modalities and then dual-modality imaging of hypoxia with multimodal probes for multiple imaging modalities. Thoughts for the future of the field close the article.

## 2. Hypoxia-Mediated Pathophysiological Changes in the Tumor Microenvironment

In addition to low oxygen partial pressure (pO_2_ < 15 mmHg (pO_2_ ≤ 2%)) [48], there are several other changes in cellular homeostasis that are associated with hypoxia in tumors (Table 1). This section describes those pathophysiological conditions that are inherent to hypoxic environments that have been used for imaging. In this review, we focus on multimodal imaging using probes that are responsive to changes in these conditions in addition to pO_2_.

### 2.1. Acidic pH (Acidosis)

A key regulator of hypoxia, HIF-1, upregulates the expression of glucose transporters, and as a consequence, the rate of glucose uptake into hypoxic cells is fast relative to that of nonhypoxic cells [49,50,51]. HIF-1 also increases the gene expression involved in the enzymatic breakdown of glucose into pyruvate and the conversion of pyruvate into lactate [52]. Lactic acid is eventually released into the extracellular matrix, resulting in acidosis. There are multiple pathways for intracellular glucose metabolism [53,54]; however, in hypoxia, HIF-1 promotes the glycolytic breakdown of glucose. Additionally, HIF-1 indirectly inhibits the enzymatic activity of pyruvate dehydrogenase that is necessary to convert pyruvate into acetyl-CoA [55,56]. Production of acetyl-CoA from pyruvate is the first step of the citric acid cycle that occurs in mitochondria. The citric acid cycle is the main mechanism that provides electrons to the electron transport chain; consequently, mitochondria do not function at their maximum levels under hypoxic conditions. To reduce the accumulation of pyruvate and nicotinamide adenine dinucleotide (NADH), HIF-1 induces lactate dehydrogenase, an enzyme needed to convert pyruvate into lactate while consuming NADH [57,58]. Although the generation of lactate is the main pathway to reducing the pH of the tumor microenvironment, other metabolic reactions also contribute to acidosis [59,60,61,62,63,64,65,66,67,68,69]. Probes that are responsive to pH or the products or intermediates of the biochemical pathways associated with acidosis are potentially useful in the study of hypoxia.

### 2.2. Aberrant Levels of Reactive Chemical Species

Reactive oxygen species are formed as byproducts of aerobic respiration and the major source of reactive oxygen species in cells is the mitochondria [70,71,72]. Some reactive oxygen species are reactive radicals, including the hydroxyl radical (^•^OH) and superoxide radical (O_2_^•−^), and comparatively less-reactive molecules including hydrogen peroxide (H_2_O_2_) [73]. Reactive oxygen species are cytotoxic and short-lived and, therefore, nearly instantaneously converted into less-reactive molecules as they form. In healthy cells, the decomposition of reactive oxygen species is mediated by antioxidants including glutathione and *N*-acetyl cysteine resulting in homeostasis between the generation and decomposition of reactive oxygen species with a slight favoring of antioxidant activity to prevent oxidative damage to cells [74,75,76]. However, HIF-1 activates enzymes, including oxidases and hydroxylases, and these enzymes release reactive oxygen species as byproducts [77]. For example, HIFs promote the activity of the nicotinamide adenine dinucleotide phosphate oxidase (NOX) enzyme complex that catalyzes the production of superoxide, protons, and NADP^+^, while consuming oxygen [78].

Abnormal quantities of reactive nitrogen species are also distinct biomarkers of tumor microenvironments [79,80]. Hypoxia induces the regulation of some isoforms of nitric oxide synthase and the release of nitric oxide radicals (NO^•^) [81,82,83]. Excess production of nitric oxide is detrimental when it reacts with superoxide to form large amounts of peroxynitrite (ONOO^−^) [84,85,86]. In healthy cells, low levels of ONOO^−^ are beneficial and regulate apoptosis. In tumor cells, excessive amounts of ONOO^−^ promote tumor immunosuppression [87], and consequently, accelerate tumor growth [88]. Therefore, imaging of reactive nitrogen species is a target for detecting and monitoring hypoxic tumors.

In addition to reactive oxygen and nitrogen species, oxidative stress also promotes the synthesis of reactive sulfur species including H_2_S via non-enzymatic pathways [89,90]. H_2_S serves as an antioxidant to protect hypoxic cells from reactive oxygen and nitrogen species [91,92]. Therefore, both reactive species and the chemicals that are responsive to reactive species could be used as markers of hypoxia for imaging probes.

### 2.3. Elevated Levels of Cellular Redox Buffers

Redox homeostasis is tightly regulated in healthy cells by redox buffers including glutathione, thioredoxin, and NADPH [93,94,95]. As a defense against the production of reactive species in hypoxic cells, HIFs increase the production of antioxidants including NADPH and glutathione [96]. These antioxidants buffer reactive oxygen and nitrogen species resulting in less-reactive molecules and, thus, adaptation to oxidative stress [97,98]. Additionally, H_2_S increases the concentration of glutathione, resulting in the scavenging of H_2_O_2_ and reducing the accumulation of reactive oxygen species [99,100]. Furthermore, one of the consequences of upregulated glycolysis and NADPH synthesis is the generation of large concentrations of glutathione. Therefore, elevated levels of glutathione and NADPH are major biomarkers of hypoxic tumors. The accumulation of reducing molecules, including NAD^+^, is a consequence of oxidative stress [101]. Necrotic cells leach these components, making the tumor extracellular matrix more reducing than the intracellular matrix [102,103]. Therefore, molecules that respond to redox regulatory molecules are important probes for imaging hypoxia.

### 2.4. Accumulation of Inorganic Ions

The imbalance between influx and efflux of Ca^2+^ in cells occurs as a consequence of the generation of reactive oxygen species in cells under oxidative stress, and studies have shown that Ca^2+^ ions accumulate in hypoxic tissues [104]. Furthermore, upon low oxygen supply, the major energy currency of cells, adenosine triphosphate (ATP), is hydrolyzed to power oxidative phosphorylation and glycolysis, resulting in an increase in the concentration of PO_4_^3−^ in cells. Consequently, hypoxic cells are usually rich in phosphate compared to healthy cells [105,106]. Therefore, probes that are responsive to these inorganic ions could be used as imaging agents for hypoxia.

## 3. Types of Imaging Modalities

The hypoxia-induced pathological changes described in the previous section can be detected using various imaging modalities [107,108,109,110,111,112,113,114,115,116]. Although each method has its own advantages and limitations with respect to accurate detection of hypoxia, single-mode detection methods have been widely used to provide details about hypoxia-induced pathophysiological changes over the past few decades [117,118]. In this section, we summarize common imaging modalities (Table 2) that are currently used to detect and study hypoxia and cancer.

### 3.1. Optical Imaging

Optical imaging is a powerful tool that can visualize subcellular morphologies based on the use of molecules that absorb and emit light [135]. Optical imaging probes described in this review for tumor imaging are fluorescent materials (for example, organic fluorophores and quantum dots) and luminescent probes (for example, lanthanide luminophores). With long-lifetime luminophores, the sensitivity of optical imaging can be increased using time-gated imaging [136,137]. However, scattering and absorbance occur when light travels through media; consequently, optical imaging is not an efficient method to image deep into tissues. In addition to luminescent probes, colorimetric imaging can be used in detecting cellular biomarkers. This modality involves compounds that change color in response to stimuli and, consequently, are used to generate pictures of the environment.

### 3.2. Ultrasound Imaging

Ultrasound imaging involves the use of sound waves with frequencies above the upper audible limit of human hearing (˃20,000 Hz) [138]. After sending ultrasound pulses into tissue using a probe, ultrasound echoes that vary with the properties of tissues are turned into images. There four different modes of ultrasound are used in current medical imaging. These include A-mode, B-mode, M-mode, and Doppler mode [139]. Examples described in this manuscript are based on B-mode (a two-dimensional ultrasound image display composed of bright dots representing the ultrasound echoes) [140] and Doppler mode (estimate the blood flow through blood vessels by bouncing high-frequency ultrasound waves off circulating red blood cells) [141]. Ultrasound is usually used to image soft tissues and organs including muscles, blood vessels, heart, and brain [142,143,144,145,146]. Because ultrasound uses sound waves, it is generally considered to be a noninvasive method of imaging. Sometimes gas-filled microspheres (microbubbles) with specific acoustic properties are used to enhance contrast in ultrasound images [147].

### 3.3. Photoacoustic (Optoacoustic) Imaging

Photoacoustic imaging, also known as multispectral optoacoustic tomography (MSOT), is an emerging technique that uses light and sound waves to generate images. Optical energy (for example, pulsed laser light) is used to excite endogenous (for example, hemoglobin or melanin) or exogenous (for example, MnO_2_ nanoparticles) chromophores that undergo thermoelastic expansion leading to the generation of pressure waves that can be detected using ultrasound transducers [148]. Photoacoustic imaging is not a sensitive method compared to fluorescence-based methods, but because the scattering of sound waves in tissue is reduced compared to light waves, photoacoustic imaging offers a contrast in tissues thicker than those that can be imaged using purely optical methods [149]. The oxy-hemo mode is used in photoacoustic imaging to measure oxygenation saturation and hemoglobin levels in oxygenated and deoxygenated blood in the tissue area or volume [150,151,152,153].

### 3.4. Single-Photon Emission Computed Tomography (SPECT)

SPECT is a medical imaging technique that is based on molecular probes labeled with gamma-emitting radionuclides distributed in tissues and organs. Some of the gamma ray-emitting radioactive tracers used in SPECT include iodine-123, technetium-99 m, and fluorine-18 [154,155]. Based on biodistribution properties, radiopharmaceuticals are accumulated in different organs or tissues. After administering SPECT probes, scintillation camera systems are used to trace the probes via gamma rays that are emitted from decaying probes accumulated in hypoxic areas. Although ionizing gamma radiation is used, SPECT is a promising imaging method in terms of high sensitivity and practically limitless depth penetration that makes it a vital molecular imaging modality in preclinical and clinical imaging of not only tumors but also neurodegenerative diseases and cardiac function [156,157,158,159].

### 3.5. X-ray Computed Tomography (X-ray CT)

X-ray CT is a noninvasive imaging modality that can be used to obtain detailed three-dimensional anatomical images [160,161]. Narrow beams of X-rays pass through samples, and an unattenuated portion of X-rays is detected using digital detectors located directly opposite the source. X-ray CT is used to image complex bone fractures; the presence of tumors in the lungs, and the presence of blood clots, hemorrhages, and injuries in the brain [162,163,164]. Dense structures can be imaged with X-ray CT, but soft tissues are difficult to observe. Probes with large electron densities that interact with X-rays can be used to enhance contrast, and these probes often include iodine or heavy metals including gold [165,166,167]. The largest limitation of X-ray CT is the ionizing radiation exposure to patients.

### 3.6. Positron Emission Tomography (PET)

PET is an imaging method based on the detection of distribution of radiopharmaceuticals labeled with a positron (β^+^)-emitting radionuclides, including fluorine-18, copper-64, and gallium-68 [168]. Positrons interact with electrons to annihilate, resulting in two gamma rays emitted at 180° from each other that are detected and used to produce images [169]. PET is a sensitive and noninvasive method that offers practically unlimited depth penetration. The rate of consumption of glucose (^18^F-labeled), blood flow, and oxygen consumption are some of the main biomedical applications of PET [170,171,172]. Therefore, PET is also used to study hypoxic tumors [173,174,175]. Although picomolar quantities of radiopharmaceuticals are administered, patient exposure to high-energy gamma radiation is a limitation of this technique. In clinical imaging, the lack of anatomical information provided by PET promotes the use of combinations of imaging modalities to provide anatomical information to overlay with PET images.

### 3.7. Cerenkov Radiation Energy Transfer (CRET) Imaging

Cerenkov radiation is the result of a charged particle passing through a dielectric medium at a speed greater than the phase velocity of light in that medium [176]. Cerenkov radiation occurs during PET imaging when the velocity of β^+^ particles exceeds the speed of light in the tissue [177]. Cerenkov radiation is usually readily absorbed by biomolecules including hemoglobin, water, and cytochromes; however, surface-weighted images can be obtained using sensitive charge-coupled devices and optical cameras [178]. Furthermore, Cerenkov energy can be effectively transferred (CRET) to nanomaterials with large Stokes shifts to produce red-shifted photonic emissions. CRET imaging is relatively a new modality that has potential use in hypoxia research [179,180].

### 3.8. Magnetic Resonance Imaging (MRI) Including Chemical Exchange Saturation Transfer (CEST) and Paramagnetic Shift (PARASHIFT) Imaging

MRI is a noninvasive imaging modality that is used to visualize morphological and functional anatomy with practically unlimited depth penetration [181]. In MRI, samples are exposed to a strong external magnetic field, and radiofrequency pulses are applied to perturb the alignment of nuclear spins within the magnetic field. As spins relax upon ceasing of the applied radiofrequency pulse, radiowaves are emitted, and these radiowaves are detected and used to generate three-dimensional images using gradients of the magnetic field [182]. Paramagnetic chemicals (contrast agents) are used to enhance the contrast of MRI by increasing the relaxation rate of nuclear spins, and the efficiency of contrast agents as a function of concentration is measured as longitudinal or transverse relaxivity (*r*_1_ or *r*_2_, respectively). Tissue environment, radiofrequency pulse sequences, and the presence of contrast agents all contribute to contrast in MRI. A variety of MRI-active nuclei are used in biomedical applications [for example, hydrogen-1 (^1^H-MRI) and fluorine-19 (^19^F-MRI)] that possess unique advantages and limitations [183,184,185,186,187,188,189]. For example, ^1^H-MRI is more sensitive but possesses a considerable background signal compared to ^19^F-MRI [190].

CEST imaging is a variation of MRI that involves chemical probes that usually contain exchangeable protons, although other exchanging nuclei can be used with non-^1^H-MRI [191]. The exchanging protons are selectively saturated using radiofrequency pulses, and those saturated protons exchange with bulk water protons to transfer some of the saturation to water, thus altering the water signal. Some CEST agents are sensitive to biomarkers including oxygen concentration and pH; therefore, responsive CEST agents can be used to study tumors [192,193,194,195,196].

PARASHIFT imaging is another variation of MRI that involves the use of metal complexes with an anisotropic magnetic susceptibility for direct detection of proton-containing tracer molecules having a negligible background signal in vivo [197]. Because anisotropic magnetic susceptibilities of some paramagnetic metal ions including Dy^3+^, Tb^3+^, Pr^3+^, Tm^3+^, Tb^3+^, Fe^2+^, and Co^2+^ can shift the proton NMR signals of chelating ligands beyond the diamagnetic range of endogenous proton (water and fat in biological systems) resonances. PARASHIFT imaging agents can also shift resonances of nuclei other than ^1^H that include nuclei in chelating ligands. For example, some ^19^F-PARASHIFT imaging agents have been used as sensitive probes for detecting overproduced chemicals during hypoxia including hydrogen peroxide [198].

### 3.9. Electron Paramagnetic Resonance Imaging (EPRI)

EPRI detects the magnetic resonance transitions between energy states of unpaired electrons in an applied magnetic field using microwave radiation [199]. EPRI can probe species with unpaired electrons including radicals and paramagnetic species. Biological systems contain small amounts of paramagnetic species having short lifetimes that are intermediates of biochemical reactions. Therefore, usually, exogeneous spin probes are used to detect paramagnetic species in biological systems. These probes form paramagnetic adducts in the presence of the species of interest to enable quantification and localization of the species. EPRI can also be used to image environmental parameters including partial pressure of oxygen, pH, and temperature [200,201]. These physical parameters alter the resonance frequencies of the exogenous spin probes. Although EPRI is a sensitive method because of the specificity of EPRI towards paramagnetic species, instrumental challenges associated with EPRI make the technique less commonly used than other imaging methods [202].

### 3.10. Magnetic Particle Imaging (MPI)

MPI is a noninvasive, quantitative, three-dimensional imaging technique that offers large signal-to-noise ratios [203,204]. Unlike structural imaging modalities–including ^1^H-MRI, ultrasound imaging, and X-ray CT–MPI is a tracer imaging technique that directly measures the amount of tracer [for example, superparamagnetic iron oxide (SPIO) nanoparticles] [205]. MPI uses two magnets pointed at each other to produce a magnetic field gradient with a field-free region at the center. The field-free region is rapidly moved across a sample to produce an image. Magnetic field gradients used in MPI can saturate the magnetization of SPIO nanoparticles outside the field-free region. The magnetization of SPIO nanoparticles changes nonlinearly as the field-free region transverses the location of an SPIO nanoparticle. This time-varying magnetization induces a voltage in the receiver coil that is linearly proportional to the number of nanoparticles and, thereby, enables the quantification of nanoparticles. MPI is a sensitive (detecting nanograms of iron per voxel) imaging modality [204].

### 3.11. Raman Spectroscopy and Surface-Enhanced Raman Spectroscopy (SERS)

Raman spectroscopy is a noninvasive and label-free optical imaging technique with large spatial resolution [206,207]. In biomedical imaging, it is based on the use of laser light that is inelastically scattered from biological samples. The difference in wavelengths between the incident and scattered photons depends on the molecular bonds present in the sample. Therefore, a detailed biochemical fingerprint of the sample can be obtained, enabling the identification of molecular differences between each molecule. Major limitations associated with Raman spectroscopy are relatively weak signal intensities and possible thermal damage to samples due to long acquisition times [208,209]. To ameliorate the signal intensity issue, metal nanostructures (for example, gold nanoparticles) are mixed with biological samples [210]. An enhancement of the Raman signal occurs as a consequence of the aggregation of metal nanostructures. Enhancement of Raman signal via the use of metal nanostructures is known as surface-enhanced Raman spectroscopy (SERS). SERS can enhance the Raman signal up to 10^15^-fold [211,212]; therefore, SERS can be used to analyze small concentrations. Compared to Raman spectroscopy, the shorter acquisition times and lower laser power used in SERS minimize thermal damage to samples [213]. However, SERS suffers from poor reproducibility due to plasmonic hot spots associated with metal nanostructures [214].

### 3.12. Mass Spectrometric Imaging

Mass spectrometric imaging is a powerful tool that can be used to obtain information about the spatial distribution of molecules in biological samples [215,216]. Without labeling, mass spectrometric imaging can be used to image the distribution of a variety of biomolecules including proteins, lipids, and glycans. In a thin section of a biological specimen, *x* and *y* coordinates are defined over the surface of the sample. A mass spectrometer is used to ionize molecules on the surface of the thin section, resulting in a collection of mass spectra mapped to spatial coordinates of the section. After collecting the spectra, computational software is used to determine the intensities of the mass-to-charge ratios, and these intensities are combined to generate heat-map images that display the relative distribution of specific mass-to-charge ratios over the surface of a sample. Tandem mass spectrometry and databases can be used to identify specific mass-to-charge ratios and, therefore, can identify specific biomolecules present in a sample [217,218]. A relatively new mass spectrometric imaging method involves a laser ablation system combined with an inductively coupled plasma mass spectrometer (LA-ICP-MS) to visualize the distribution of elements of interest within solid samples in two or three dimensions [219,220,221]. Briefly, a laser pulse ablates a sample, and the aerosol of the ablated sample passes through the high-energy plasma to atomize and ionize the elements in the aerosol. The resulting ions are transferred from the plasma to a mass analyzer to measure the intensity of elements of interest. These intensities correspond to the abundance of elements in the ablated sample. Although mass spectrometric imaging is promising, it is a destructive technique.

Each single imaging modality possesses inherent advantages and limitations. Therefore, it is desirable to combine multiple imaging modalities that contain complementary advantages to study hypoxic tumors while overcoming the limitations of each imaging mode and serving as a conformation for single-mode analyses.

## 4. Imaging Hypoxia with Multiple Probes and Multiple Modalities

In this section, we describe the use of dual- and multi-modal imaging modalities to detect hypoxia and hypoxia-related pathological conditions (Table 3). We describe the use of two imaging modalities followed by the use of three imaging modalities to image hypoxia-relevant properties. Overall advantages of this method include the ability to obtain information about the tumor microenvironments, overcome limitations of individual methods, and enhance the accuracy of details obtained from both imaging modalities.

### 4.1. MRI and Mass Spectrometric Imaging

**Figure 2:**
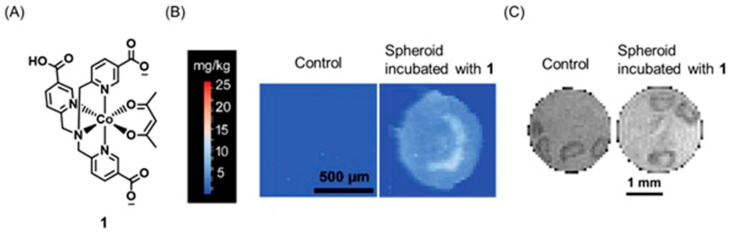
(**A**) Chemical structure of **1** in its Co^III^ oxidation state. (**B**) Laser ablation inductively coupled plasma mass spectrometric images of the control sample and tumor spheroid incubated with **1**. (**C**) *T*_1_-weighted MR images of the control sample and tumor spheroid incubated with **1**. Both laser ablation inductively coupled plasma mass spectrometric image and *T*_1_-weighted image of the tumor spheroid incubated with **1** showed accumulation of **1** in the inner hypoxic regions which is known to have a reducing and acidic environment. Adapted with permission from O’Neill, E. S.; Kaur, A.; Bishop, D. P.; Shishmarev, D.; Kuchel, P. W.; Grieve, S. M.; Figtree, G. A.; Renfrew, A. K.; Bonnitcha, P. D.; New, E. J. Hypoxia-responsive cobalt complexes in tumor spheroids: laser ablation inductively coupled plasma mass spectrometry and magnetic resonance imaging studies. *Inorg. Chem.*, **2017**, *56*, 9860–9868. Copyright 2017 American Chemical Society [236].

MRI and mass spectrometric imaging are combined to extract the spatial distribution of biomolecules with a high resolution [222,223]. A combination of mass spectrometric imaging with MRI can potentially bridge the gap between the ex vivo and in vivo imaging techniques [224]. Therefore, combined MRI and mass spectrometric imaging are powerful tools that can be used to study several diseases including cardiovascular diseases, infections, and cancer [225,226,227,228,229,230,231]. Laser ablation inductively coupled plasma mass spectrometry is a sensitive method that can be used to determine metals and chemicals in biological environments [232,233,234,235]. Therefore, laser ablation inductively coupled plasma mass spectrometry, combined with MRI provides a detailed understanding of the physiological characteristics of the tumor microenvironment. New and co-workers reported a series of cobalt-containing tris(2-pyridylmethyl)amine pro-drugs with the varying charge for distribution and activity in three-dimensional tumor spheroids using laser ablation inductively coupled plasma mass spectrometry and MRI [236]. Out of all complexes that they studied, complex **1** was selective for hypoxic regions of cells both in accumulation and activation. This observation was attributed to the fact that a Co^III^-containing complex can reduce to a Co^II^-containing complex and favorably accumulate in reducing acidic environments that are characteristic of hypoxic regions of the tumors. Furthermore, electroneutrality obtained upon protonation of the carboxylic group of **1** in the acidic extracellular region facilitates the accumulation of **1** in the inner hypoxic regions (Figure 2) [236]. As supported by MRI studies, **1** showed an increase in MR signal intensity in the inner regions of hypoxic tumors. This observation can be attributed to the reduction in diamagnetic Co^III^ to paramagnetic Co^II^ in reducing environments which are known to be prevalent in hypoxic tumors.

### 4.2. Photoacoustic and Ultrasound Imaging

**Figure 3:**
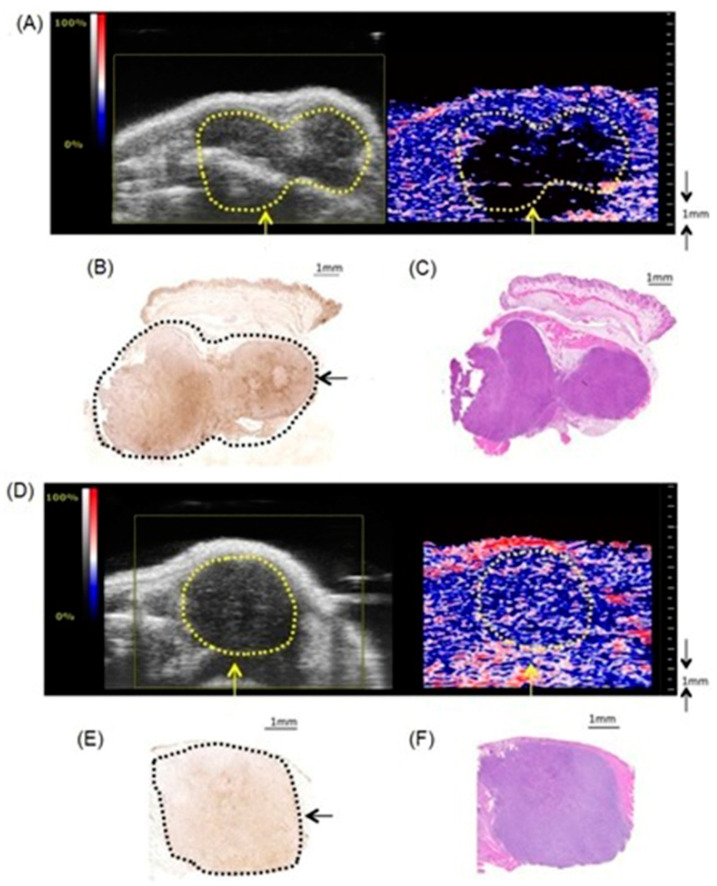
Imaging of tumor hypoxia in subcutaneous pancreatic xenografts. (**A**) Representative oxy-hemo photoacoustic image of a hypoxic tumor. B-mode image (left panel) and oxygen saturation map (oxy-hemo photoacoustic imaging, right panel). The heat map represents oxygen saturation level, ranging from 100% (red) to 0% (dark blue). The yellow dashed line defines the tumor region. (**B**) Representative hypoxia staining (brown color) of the same tumor. The black dashed line defines the tumor region. (**C**) Representative hematoxylin and eosin staining of the same hypoxic tumor. (**D**) Representative oxy-hemo photoacoustic image of a non-hypoxic tumor. B-mode image (left panel) and oxygen saturation map oxy-hemo photoacoustic image (right panel). The heat map represents oxygen saturation level, ranging from 100% (red) to 0% (dark blue). The yellow dashed line defines the tumor region. (**E**) Representative pimonidazole staining of the non-hypoxic tumor, which lacks brown staining. The black line represents the tumor region. (**F**) Representative hematoxylin and eosin staining of the non-hypoxic tumor. Adapted with permission from Gerling, M.; Zhao, Y.; Nania, S.; Norberg, K. J.; Verbeke, C. S.; Englert, B.; Kuiper, R. V.; Bergström, A.; Hassan, M.; Neesse, A.; Löhr, J. M.; Heuchel, R. L. Real-time assessment of tissue hypoxia in vivo with combined photoacoustics and high-frequency ultrasound. *Theranostics*, **2014**, *4*, 604 [243].

Current biomedical imaging uses the advantages of photoacoustic imaging with inherently co-registered high-frequency ultrasound imaging that enables accurate mapping of tumors [237,238,239]. These advantages include the ability of ultrasound imaging to produce anatomical data and the ability of photoacoustic imaging to sensitively detect light-absorbing molecules that are present in deep tissues with excellent resolution [240,241,242]. Co-registration of photoacoustic imaging and ultrasound imaging provides optical contrast and high-resolution anatomical data. Because sound waves echo differently from fluid-fill bubbles (in contrast-enhanced ultrasound imaging) and solid masses, ultrasound can be used to distinguish tumors from healthy tissues. A study by Heuchel and co-workers used high-frequency ultrasound imaging and photoacoustic imaging to assess hypoxia and tumor blood flow in murine models of intra-abdominal tumors including pancreatic and colon cancer [243]. They also observed a positive correlation of imaging data with immunohistochemical evidence of hypoxia. The study is based on the measurement of tissue oxygen saturation based on hemoglobin and deoxyhemoglobin. Absorption spectra of hemoglobin differ with oxygenation. Dual-wavelength photoacoustic imaging can be used to separately measure tissue concentration of oxyhemoglobin and deoxyhemoglobin and therefore, can calculate focal tissue oxygen saturation. Thermoelastic waves that result due to optical excitation of molecules are detected by ultrasound receivers. Accordingly, hypoxic, and non-hypoxic regions of subcutaneous pancreatic tumor-bearing murine models were imaged using combined photoacoustic and ultrasound imaging (Figure 3) [243].

**Figure 4:**
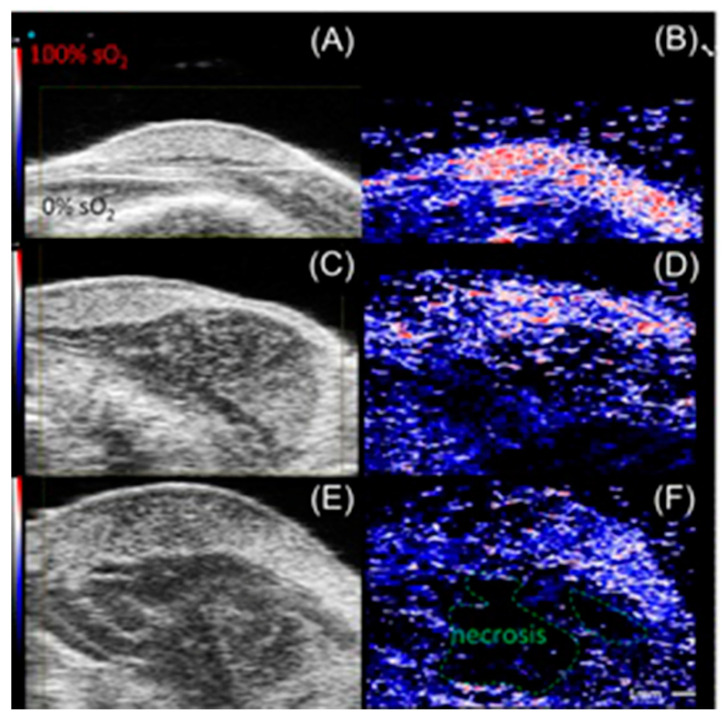
Representative set of anatomic images of a tumor recorded in B-mode and oxy-hemo mode (**A**,**C**,**E**). Different tissues (brighter shade and darker areas) correspond to photoacoustic panels (**B**,**D**,**F**) of small, medium, and large tumors and depict vital areas (brighter pixels in a B-mode and oxygenated areas in photoacoustic oxy-hemo mode are blue and red, respectively) and less oxygenated and necrotic areas inside tumors. Scale bar represents 1 mm. Adapted from *Ultrasound in Medicine and Biology*, *47*, Keša, P.; Pokorná, E.; Grajciarová, M.; Tonar, Z.; Vočková, P.; Trochet, P.; Kopeček, M.; Jakša, R.; Šefc, L.; Klener, P. Quantitative in vivo monitoring of hypoxia and vascularization of patient-derived murine xenografts of mantle cell lymphoma using photoacoustic and ultrasound imaging, 1099–1107, Copyright 2021, with permission from Elsevier [248].

High-frequency color Doppler imaging enables the visualization of blood flow with high resolution. Studies revealed that doppler ultrasound imaging can visualize blood flow with high-resolution [244,245,246,247]. Keša and co-workers combined these properties of two imaging modalities to study tumor oxygenation and vascularization in vivo during the growth of subcutaneously implanted patient-derived xenograft lymphomas (Figure 4) [248]. They reported that this multi-modal ultrasound-photoacoustic imaging method offers a new approach for rapid and direct assessment of oxygen saturation of patient-derived xenograft lymphomas without the use of exogenous contrast agents. Its high-resolution nature provides details as fine as 50 µm, as demonstrated by the detection of blood flow.

**Figure 5:**
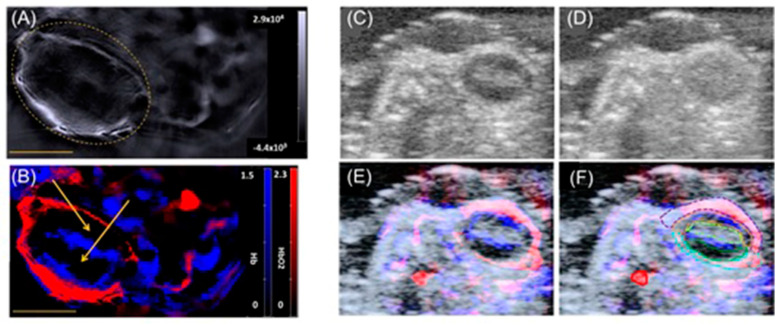
(**A**) A reconstructed single wavelength multispectral optoacoustic tomography image of tumor-bearing mouse at 800 nm. The yellow dashed line indicates the tumor region. (**B**) The corresponding deconvoluted image, showing the distribution of hemoglobin and oxyhemoglobin components in blue and red, respectively. (**C**) An ultrasound image of the tumor-bearing mouse before microbubble injection. (**D**) The ultrasound image of the tumor 40 s after microbubble injection. (**E**) An overlay of the spectrally unmixed optoacoustic image on the ultrasound image after rigid registration of the two images. (**F**) The graphic overlay on (**E**) to indicate the regions of interest analyzed, color-coded as red (blood vessel), purple dashed (upper rim), orange (upper body), light green dashed (core), dark green (lower body), and cyan dashed (lower rim). The yellow scale bars in (**A**,**B**) represent 5 mm. Adapted from *Photoacoustics*, *8*, Shah, A.; Bush, N.; Box, G.; Eccles, S.; Bamber, J. Value of combining dynamic contrast-enhanced ultrasound and optoacoustic tomography for hypoxia imaging, 15–27, Copyright (2017), with permission from Elsevier [249].

Shah and co-workers reported a method to assist the co-registration of optoacoustic images with dynamic contrast-enhanced ultrasound images to study pre-clinical tumor models [249]. A spatial correlation was observed between the dynamic contrast-enhanced ultrasound imaging properties and tumor blood oxygen saturation and hemoglobin as estimated using photoacoustic imaging. Photoacoustic imaging can detect hemoglobin and assess oxygenation. Total hemoglobin and blood oxygen saturation are important methods to infer hypoxia and its spatial and temporal variation. Combined imaging performed using a tumor modal showed that ultrasound image of the tumor hypoxic region is spatially correlated with the optoacoustic imaging, which was used to determine blood-oxygen saturation and hemoglobin (Figure 5) [249].

**Figure 6:**
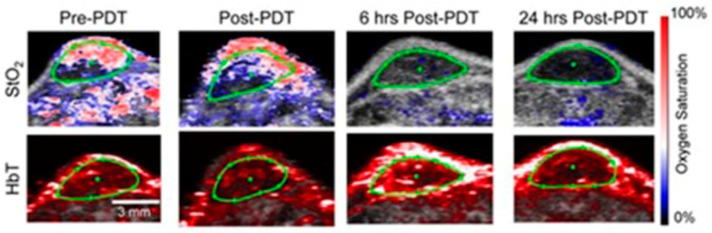
Combined ultrasound and photoacoustic images of responders in 1 h post-injection of photosensitizer before and after (immediate, 6 h, and 24 h) photodynamic therapy (PDT). (**Top row**) Ultrasound image in grayscale overlaid with oxygen saturation (StO_2_) map where blue and red represent hypoxic and oxygenated regions, respectively. (**Bottom row**) Ultrasound image in grayscale overlaid with total hemoglobin image (HbT). The green circle indicates the tumor region identified using ultrasound image. Adapted with permission from Mallidi, S.; Watanabe, K.; Timerman, D.; Schoenfeld, D.; Hasan, T. Prediction of tumor recurrence and therapy monitoring using ultrasound-guided photoacoustic imaging. *Theranostics*, **2015**, *5*, 289 [250].

Hasan and co-workers reported an ultrasound-guided photoacoustic imaging method to monitor tumor recurrence and therapy of a murine model of glioblastoma [250]. Because photoacoustic imaging can provide a three-dimensional map of tumor blood oxygen saturation by measuring oxygenated and deoxygenated hemoglobin photoacoustic imaging can be used to monitor the progress of photodynamic therapy and tumor recurrence. Photodynamic therapy consumes oxygen to generate cytotoxicity and thus causing changes in blood oxygen saturation. Therefore, by assessing blood oxygen saturation, hypoxic and normoxic tumors can be identified. The reported study distinguishes oxygenated tumors and hypoxic tumors using ultrasound and photoacoustic imaging. Imaging maps were spatially correlated with histological and immunofluorescence studies (Figure 6) [250].

### 4.3. MRI and SPECT Imaging

**Figure 7:**
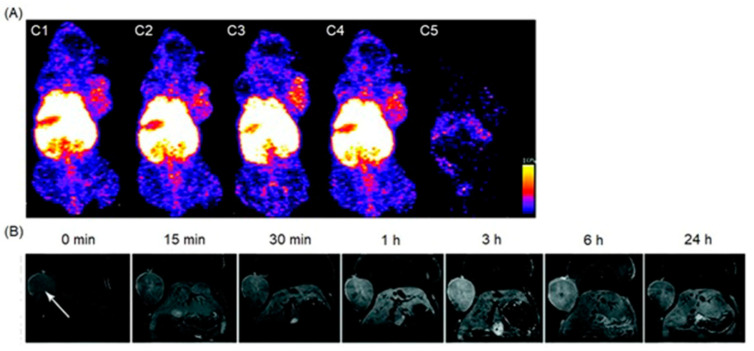
(**A**) SPECT images (C1 through C5) of a tumor-bearing mouse after intravenous administration of ^99m^Tc-containing MnO_x_-based mesoporous silica nanoparticles were combined with ^99m^Tc. The white arrow indicates the region where the tumor is located. (**B**) *T*_1_-weighted MRI images of the tumor-bearing mouse before and after intravenous administration of MnO_x_-containing mesoporous silica nanoparticles. Adapted with permission of the Royal Society of Chemistry, from ^99m^Tc-conjugated manganese-based mesoporous silica nanoparticles for SPECT, pH-responsive MRI, and anti-cancer drug delivery, Gao, H.; Liu, X.; Tang, W.; Niu, D.; Zhou, B.; Zhang, H.; Liu, W.; Gu, B.; Zhou, X.; Zheng, Y.; Sun, Y.; Jia, X.; Zhou, L. *8*, 47, **2016**; permission conveyed through Copyright Clearance Center, Inc. [256].

SPECT possesses excellent sensitivity and lower resolution compared to MRI which has greater resolution and lower sensitivity [251,252]. Therefore, dual-modal imaging using SPECT and MRI exhibits both excellent sensitivity and high resolution. Mn^II^-containing contrast agents are regarded as promising alternatives to Gd^III^-containing contrast agents [253,254,255]. Zhou and co-workers reported the combining of MnO_x_-containing mesoporous silica nanoparticles with ^99m^Tc to achieve SPECT-MRI dual-modal imaging contrast agents [256]. MnO_x_ nanoparticles dispersed within the mesopores of mesoporous silica nanoparticles to synthesize Mn-based contrast agents for *T*_1_-weighted MRI. The pH of the tumor microenvironment is lower than that of normal tissues due to its upregulated glycolytic metabolism. The Mn^II^ ions of the MnO_x_ nanoparticles can therefore dissociate under the weak acidic microenvironment of the tumor to increase the relaxivity for efficient *T*_1_-weighted MRI. Under these conditions, only the MnO_x_ nanoparticles within the tumor can dissociate into Mn^II^ ions, which is beneficial for the detection of the tumor microenvironment, thereby detecting the tumor at an early stage. ^99m^Tc is a commonly used radiotracer for SPECT, due to its suitable half-life (*t*_1/2_ = 6.02 h) and major γ-line at 140 keV that enables high-quality optimal imaging and avoids excessive patient radiation exposure. The enhanced SPECT–MRI image quality in tumor-bearing mice was further evaluated using 7.0 T micro-MRI and SPECT which provides semi-quantitative information about the tumor (Figure 7) [256].

**Figure 8:**
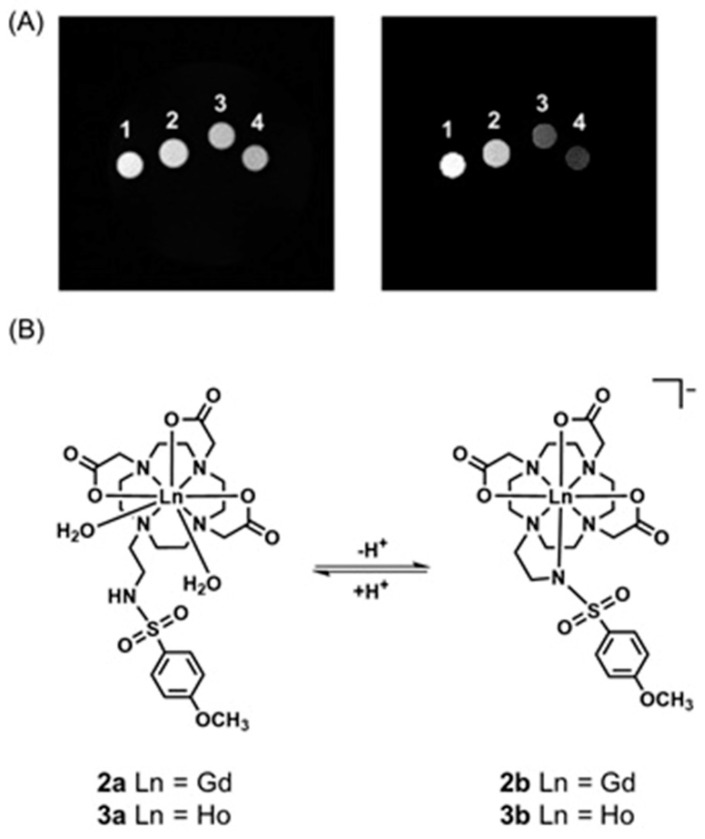
(**A**) MRI images of four samples at pH (1) 5.9, (2) 6.4, (3) 6.9, and (4) 7.4: (left) before normalization and (right) after normalization with respect to the Gd^III^ concentration of the four samples determined via SPECT measurements. (**B**) Chemical structures of complexes **2** and **3**. Adapted with permission of the Royal Society of Chemistry, from Dual MRI-SPECT agent for pH-mapping, Gianolio, E.; Maciocco, L.; Imperio, D.; Giovenzana, G. B.; Simonelli, F.; Abbas, K.; Bisi, G.; Aime, S. *47*, 5, **2011**; permission conveyed through Copyright Clearance Center, Inc. [257].

Aime and co-workers reported ^1^H-MRI and SPECT to detect changes in pH using Gd^III^-containing complex **2** and its isostructural ^166^Ho^III^-containing analog **3** as a radiotracer [257]. In this study, the radiotracer acted as a calibration standard to infer the concentration of **2**. In acidic pH conditions (pH < 5), the sulfonamide bearing arm is not coordinated and the coordination sphere of the Gd^III^ ion (**2a**) therefore, the coordination sphere is filled with two water molecules (*q* = 2). At pH 5 and 300 MHz, the observed relaxivity is 7.8 mM^−1^ s^−1^. Upon deprotonation, the sulfonamide group bearing a negatively charged nitrogen enters the coordination sphere of the Gd^III^ ion yielding a structure (**2b**) containing no water molecules in the inner coordination sphere (*q* = 0). The relaxivity of the **2b** at pH 8 and 300 MHz is 2.6 mM^−1^ s^−1^. ^166^Ho (*t*_1/2_ = 26.8 h) emits γ-radiation that can be detected using a gamma camera. Complexes **2** and **3** are expected to share the same biodistribution when administered in vivo, and hence, the mixture of **2** and **3** can be used as a dual MRI/SPECT probe (Figure 8) [257].

### 4.4. MRI and X-ray CT Imaging

In some cases, MRI and X-ray CT are complementary to each other [258]. MRI produces images with high resolution and accuracy, and X-ray CT produces images with high specificity [259,260]. Combined MRI and X-ray CT enable the precise determination of several diseases through the generation of anatomical images [261,262,263].

**Figure 9:**
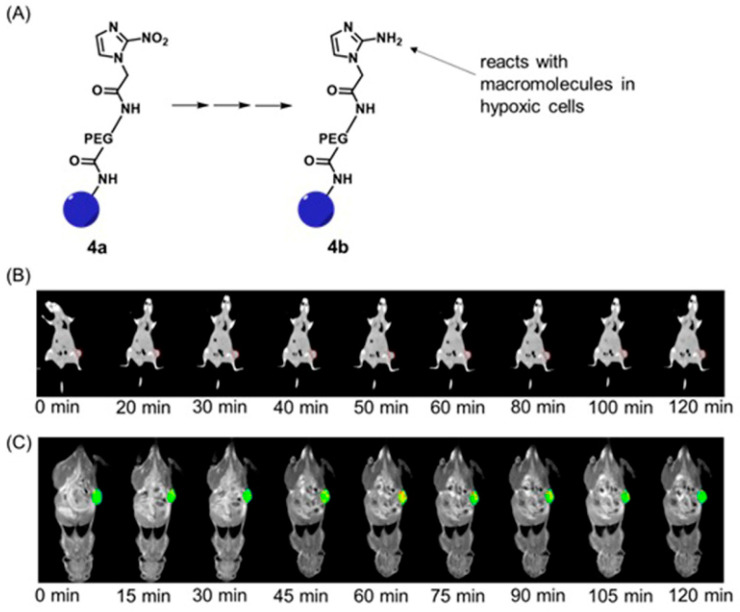
(**A**) Schematic illustration of the reduction in the nanohybrid for dual-mode MRI–X-ray CT imaging. Blue circles represent dendrimer nanohybrid. The nanohybrid accumulates in tumors is reduced in hypoxic cells and binds with biomacromolecules. (**B**) In vivo X-ray CT images of the hypoxic tumor at different time points post intravenous injection of the nanohybrid. The red dashed circle in each panel indicates the tumor site. The tumor site has the highest X-ray CT intensity at 60 min, and then the intensity starts to decrease gradually. (**C**) In vivo *T*_1_-weighted MRI images of the hypoxic tumor at different time points post intravenous injection of the nanohybrid. The tumor site has the highest MRI intensity at 60 min, and then the intensity starts to decrease gradually. Adapted with permission from Fan, Y.; Tu, W.; Shen, M.; Chen, X.; Ning, Y.; Li, J.; Chen, T.; Wang, H.; Yin, F.; Liu, Y.; Shi, X. *Advanced Functional Materials*, John Wiley and Sons [264].

Liu, Shi, and co-workers reported multifunctional dendrimer nanohybrids that have Gd^III^-containing complexes for *T*_1_-weighted MRI, Au nanoparticles for X-ray CT imaging, and 2-nitroimidazole moieties for hypoxia-responsiveness [264]. The reducing environment associated with hypoxic causes the nitro groups in the 2-nitroimidazole moieties in **4a** to reduce to amines, **4b**. Subsequently, the nanohybrids conjugate with macromolecules in hypoxic cells. As a consequence, the nanohybrid accumulates in hypoxic cells (Figure 9). After accumulation, Gd^III^-containing complexes produce *T*_1_-weighted contrast enhancement, and Au nanoparticles enhance X-ray CT images. The nanohybrids were tested for cytocompatibility, specificity to be endocytosed by hypoxic cancer cells, and the ability to be used for specific dual-mode X-ray CT–MR imaging of the hypoxic tumors in vivo (Figure 9) [264].

**Figure 10:**
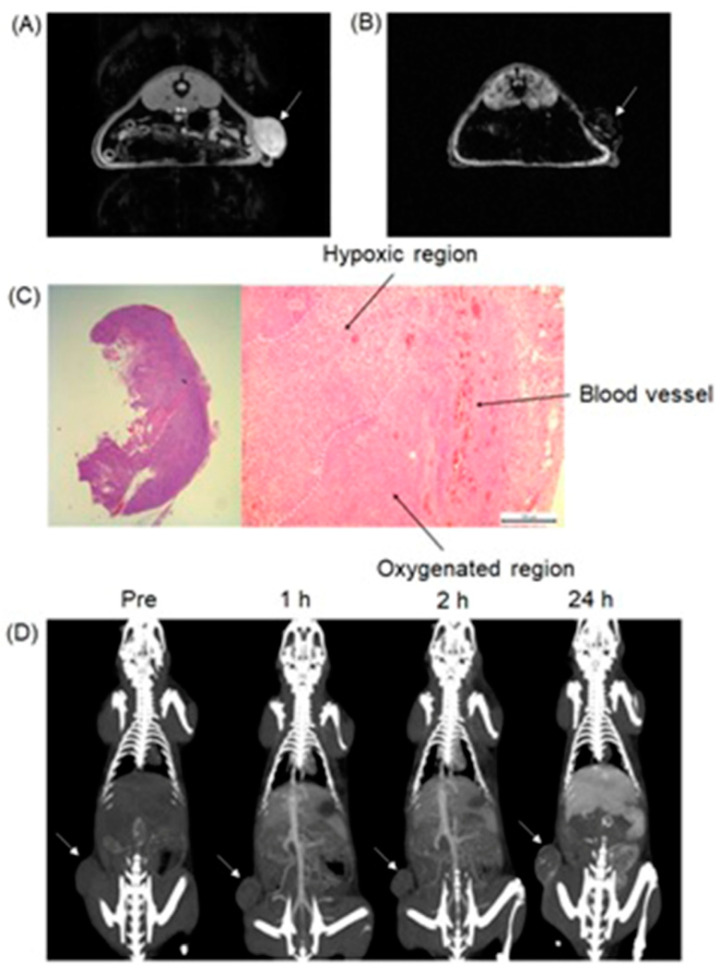
*T*_2_-weighted MRI image (**A**) before and (**B**) after intravenous injection of the Fe_3_O_4_/TaO_x_ core/shell nanoparticles prepared at a concentration 0.4 mg/mL Fe_3_O_4_. Tumor site indicated in a white arrow. (**C**) Hematoxylin and eosin-stained section of the tumor. Whole-tumor image (left) shows that the interior is inhomogeneous. Magnified image (right) of tumor shows hypoxic region, oxygenated region, and blood vessel. Scale bar represents 200 µm. (**D**) Coronal maximum intensity projection images of whole-body X-ray CT images of a rat before and after intravenous injection of Fe_3_O_4_/TaO_x_ core/shell nanoparticles. White arrows indicate the tumor site. Adapted with permission from Lee, N.; Cho, H. R.; Oh, M. H.; Lee, S. H.; Kim, K.; Kim, B. H.; Shin, K.; Ahn, T. Y.; Choi, J. W.; Kim, Y. W.; Choi, S. H.; Hyeon, T. Multifunctional Fe_3_O_4_/TaOx Core/Shell nanoparticles for simultaneous magnetic resonance imaging and X-ray computed tomography. *J. Am. Chem. Soc.*, **2012**, *134*, 10309–10312. Copyright 2012 American Chemical Society [265].

Choi, Hyeon, and co-workers reported multifunctional Fe_3_O_4_/TaO_x_ core/shell nanoparticles for simultaneous magnetic resonance and X-ray computed tomography that enables differentiation between oxygenated and hypoxic regions of tumors and map tumor-associated blood vessels [265]. The study showed that signal increased linearly in the CT images, in the CT images, whereas *T*_2_-weighted signal intensity decreased with increasing nanoparticle concentration. Despite the sensitivity of *T*_2_-weighted MRI, blood-pool imaging is difficult because *T*_2_ contrast agents reduce the signal intensity with increasing concentration of the contrast agent. Nanoparticles accumulate in the tumor via the enhanced permeation and retention effect. Because of the limited vasculature, delivery of nanoparticles to the hypoxic tumor core is difficult; therefore, the concentration of the nanoparticles is lower in the hypoxic core compared to the periphery of the tumor and the tumor-associated blood vessels. Before intravenous injection of the nanoparticles, the tumor appeared homogeneous and bright in the MRI image. After administration of the nanoparticles, the tumor appeared inhomogeneous, although the overall signal of the tumor was attenuated (Figure 10). The signal reduction in the peripheral region of the tumor was severe that this region appeared dark, whereas the tumor core exhibited gray color. Histological studies of the dissected tumor slice revealed that the inhomogeneous tumor consisted of a peripheral oxygenated region and a hypoxic core region (Figure 10). The cells in the peripheral region are healthy because sufficient oxygen and nutrients were supplied by peripheral vasculature. Upon administration of nanoparticles, signal enhancement of blood vessels, including the tumor-associated vessels and the periphery of the tumor, were observed in volume-rendered X-ray CT images. Therefore, combined X-ray CT and MRI differentiated oxygenated and hypoxic regions of a tumor and tumor-associated blood vessels [265].

### 4.5. MRI and PET Imaging

**Figure 11:**
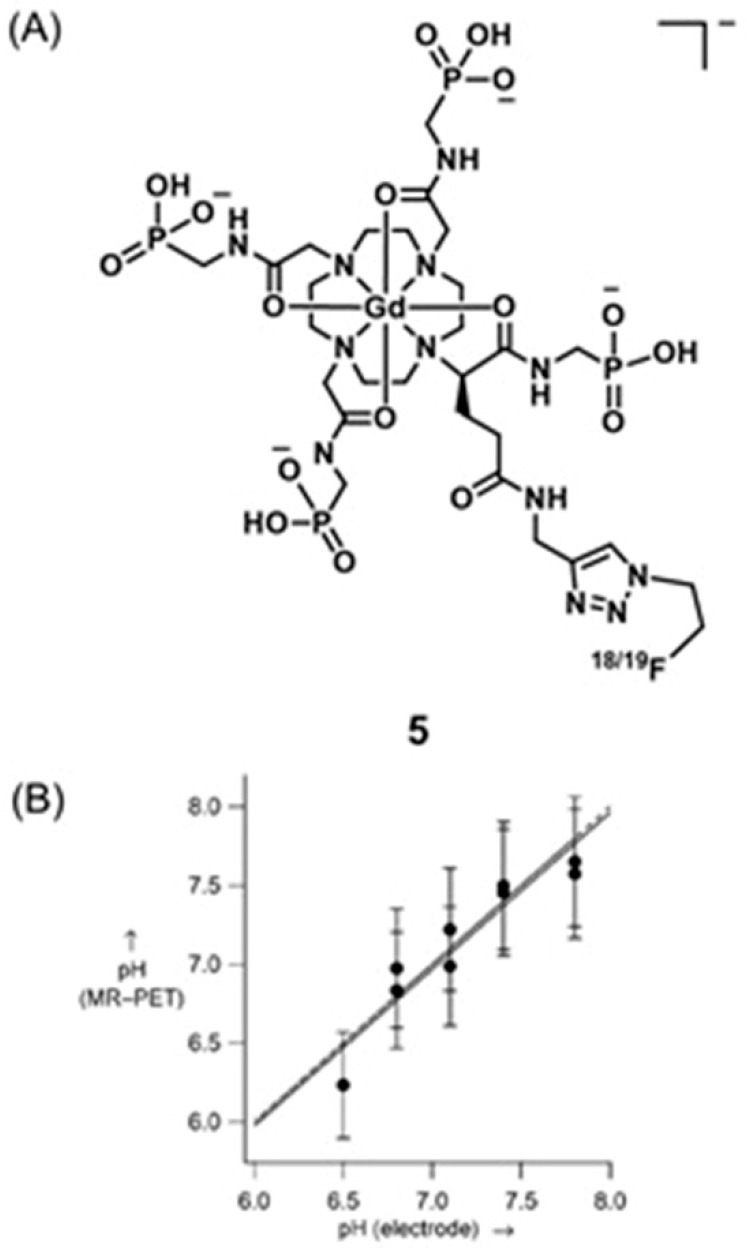
(**A**) Chemical structure of **5** used for dual-mode PET and ^1^H-MRI for quantitative pH measurements. Phosphonates are drawn singly protonated because of the expected first (p*K_a_* ≈ 2–3) and second (p*K_a_* ≈ 7–8) p*K_a_* values. (**B**) pH determined with the MRI/PET technique versus pH determined using a glass electrode. The solid line represents a linear fit of the data, and the dashed line represents a hypothetical 1:1 correspondence between the *x*- and *y*-axes. Adapted with permission from Frullano, L.; Catana, C.; Benner, T.; Sherry, A. D.; Caravan, P. *Angewandte Chemie International Edition*, John Wiley and Sons [266].

Caravan and co-workers reported a bimodal ^1^H-MRI and PET agent **5** for quantitative pH imaging [266]. They used the positron-emitting isotope ^18^F for PET imaging and Gd^III^-containing moiety for *T*_1_-weighted MRI. Images were acquired using MRI and PET on two sets of samples. In one set, *T*_1_ was varied, and the probe concentration was kept constant. In the other set, *T*_1_ was kept constant, and the probe concentration was varied. In both sets of samples, pH was varied. By combining PET and MRI datasets, pH values can be determined (Figure 11). Because acidic pH is an important biomarker of hypoxic tumors, their study was used to report pH values quantitatively and noninvasively (Figure 11) [266].

**Figure 12:**
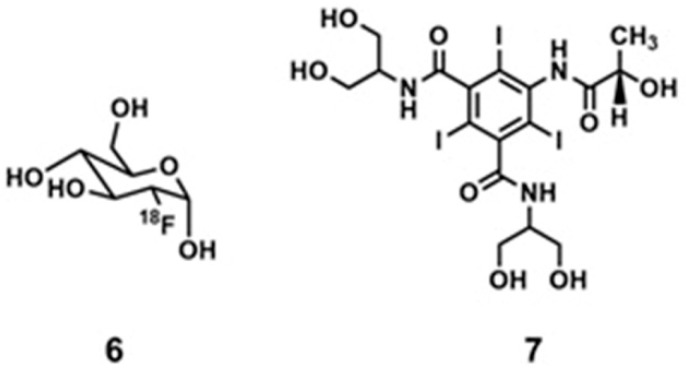
Chemical structures of **6** and **7**.

Aime and co-workers reported a noninvasive method for imaging tumor metabolism and pH by combining PET and CEST-MRI [267]. They assessed in vivo correlation between tumor ^18^F-fluorodeoxyglucose, **6**, uptake, and extracellular pH values in a murine modal bearing a breast cancer (Figure 12). Hypoxic tumors exhibit enhanced glucose uptake and glycolysis which leads to enhanced production of lactic acid that decreases extracellular pH. Therefore, dysregulated tumor pH and upregulated glucose metabolism are hallmarks of tumors that are useful information to gain an understanding of tumor biology. In the method described by Aime and co-workers, pH-responsive contrast agent **7** was used to demonstrate the occurrence of tumor pH. The extent of uptake of **6** was quantified using PET imaging. The results showed that high uptake of **6** corresponds to lower extracellular pH in hypoxic tumor microenvironments [267].

### 4.6. PET and X-ray CT Imaging

**Figure 13:**
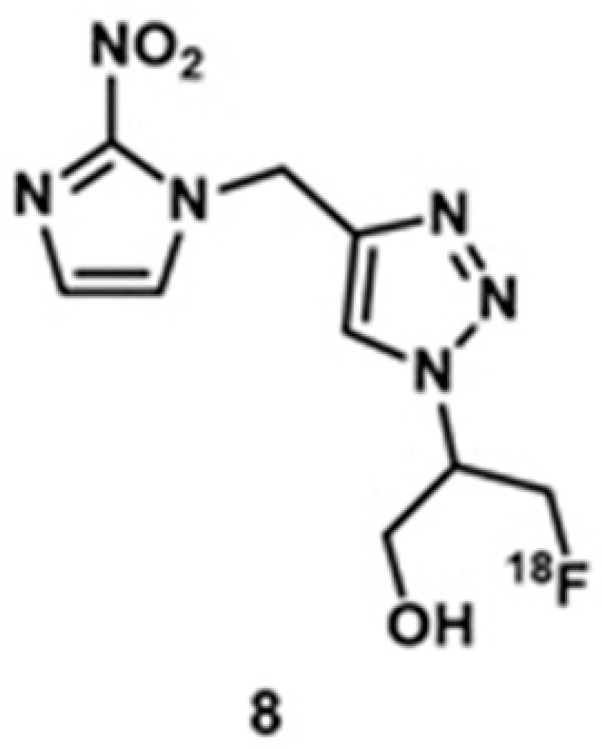
Chemical structure of **8**.

In clinical imaging, a combination of anatomic X-ray CT and functional PET is frequently used for comprehensive visualization, detection, and staging of malignancies [268,269,270]. Increased glucose metabolism is associated with hypoxia in the majority of solid tumors [271,272]. Because hypoxia is related to poor prognosis, promotes resistance to treatments, and increases metastatic potential, it is important to visualize and quantify tumor metabolism and hypoxia. Zegers and co-workers reported a comparison of tumor metabolism hypoxia using **6** and **8** for PET–X-ray CT imaging (Figure 13) [273]. Compound **8** is a nitroimidazole with pharmacokinetically attractive properties that has been used in preclinical imaging. They selected non-small cell lung cancer bearing patients because hypoxia is present in the majority of non-small cell lung cancers that can be visualized and quantified using functional PET imaging with radiolabeled 2-nitroimidazoles. Because of the reduced oxygen concentration in hypoxic cells, the nitroimidazole moiety in **8** undergoes reduction and subsequently binds with cellular macromolecules. Furthermore, because **6** is an analog of glucose, it provides functional information based on the increased glucose uptake and glycolysis of cancer cells. This study shows that **8** can be used to visualize and quantify tumor hypoxia in patients with non-small cell lung cancer. Furthermore, results showed that patients with larger tumor sizes and higher uptake are more likely to bear a larger hypoxic volume [273].

**Figure 14:**
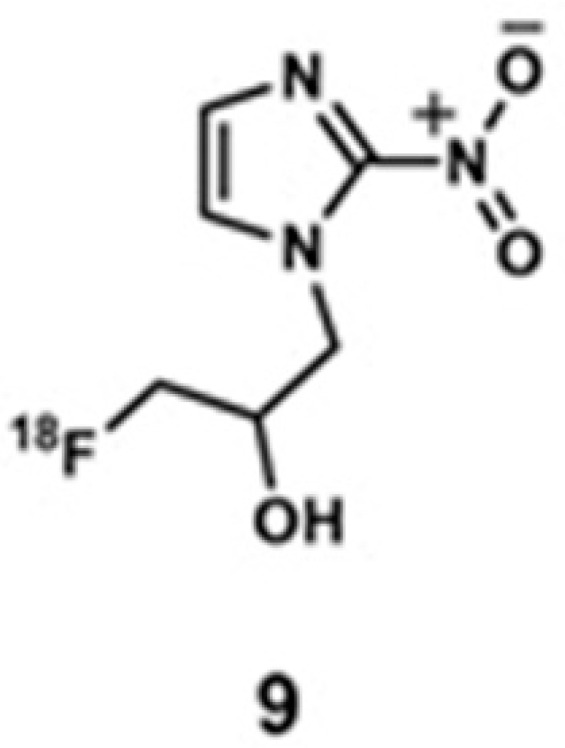
Chemical structure of **9**.

Guan and co-workers reported a comparison between **8** and ^18^F-fluoromisonidazole, **9**, in human head and neck cancer [274]. Owing to the reducing environment in hypoxic cells, the nitro group in the 2-nitroimidazole moiety in **9** reduces to an amine. Subsequently, the nanohybrid forms conjugate with macromolecules in hypoxic cells via the amine group. As a consequence, an accumulation of **9** in the hypoxic cells can be detected using PET imaging. The uptake region of **6** was compared with the uptake regions of **8** and **9**, and **8** and **9** highlight similar hotspots as **6** (Figure 14) [274].

**Figure 15:**
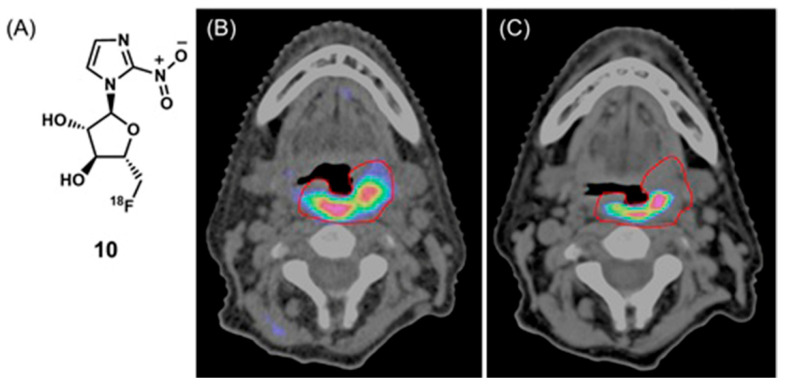
(**A**) Chemical structure of **10**. (**B**) PET–X-ray CT scan of a patient with oropharyngeal cancer before radiation therapy. The red solid line indicates the gross tumor volume. (**C**) Hypoxic volume after 12 Gy radiation therapy. After radiation therapy, the hypoxic volume decreased from 30.9 to 13.7 cm^3^. Adapted from *Radiotherapy and Oncology*, *105*, 1, Mortensen, L. S.; Johansen, J.; Kallehauge, J.; Primdahl, H.; Busk, M.; Lassen, P.; Alsner, J.; Sørensen, B. S.; Toustrup, K.; Jakobsen, S.; Petersen, J.; Petersen, H.; Theil, J.; Nordsmark, M.; Overgaaerd, J. FAZA PET/CT hypoxia imaging in patients with squamous cell carcinoma of the head and neck treated with radiotherapy: results from the DAHANCA 24 trial. 14–20, Copyright 2012, with permission from Elsevier [275].

Mortensen and co-workers evaluated an ^18^F-fluoroazomycin arabinoside, **10**, for staging tumor hypoxia in patients with head and neck squamous cell carcinoma using PET–X-ray CT imaging [275]. The reducing environment of hypoxic regions causes the nitro group in **10** to reduce to an amine group. Subsequently, the molecules form conjugates with macromolecules in hypoxic cells via the amine. As a consequence of conjugation, accumulation occurs in hypoxic cells. Patients with head and neck squamous cell carcinoma were treated with radiotherapy (66–76 Gy). Hypoxic volume was delineated using **10** and PET–X-ray CT imaging (Figure 15) [275].

### 4.7. SERS and Photoacoustic Imaging

Photoacoustic imaging has enhanced tissue penetration depth compared to fluorescence imaging and enables real-time imaging [122,123,276,277,278]. SERS has high specificity and sensitivity due to its unique fingerprint vibration spectrum, and as a consequence, can be used for multiplex imaging [279,280,281]. Therefore, combined SERS and photoacoustic imaging is an approach that has been applied for cell and tumor imaging [282,283].

**Figure 16:**
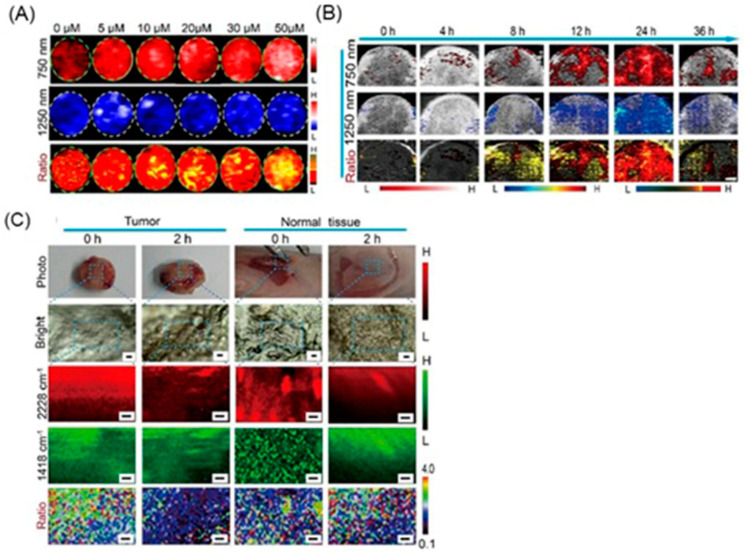
Representative in vitro and in vivo images. (**A**) Photoacoustic images at 750 and 1250 nm and the corresponding ratio images of the core-satellite with the changes of hydrogen peroxide concentration. In the presence of hydrogen peroxide, the substrate loaded in the core–shell is converted to an oxidized form with an increased photoacoustic signal at 750 nm, while the signal at 1250 nm of the nanorod remains unchanged, forming the ratiometric PA_750_/PA_1250_ signal. (**B**) Photoacoustic imaging at 750 and 1250 nm, and the corresponding signal ratio, of mice with subcutaneous 4T1 tumors at different times after intravenous injection of the nanoprobe (scale bar is 1 mm). (**C**) Confocal Raman imaging at 2228 cm^−1^ (red color, change peak) and 1418 cm^−1^ (green color, constant peak) and the ratio image (*I*_2228_/*I*_1418_) of normal and tumor tissues before and two hours after injection of the nanoprobe (scale bar is 10 µm). Adapted with permission from Li, Q.; Ge, X.; Ye, J.; Li, Z.; Su, L.; Wu, Y.; Yang, H.; Song, J. *Angewandte Chemie International Edition*, John Wiley and Sons [285].

Excessive production of reactive oxygen species, including hydrogen peroxide, is an important indicator of hypoxic tumors [284]. Song and co-workers reported a dual ratiometric SERS and photoacoustic real-time quantitative visualizing method to detect hydrogen peroxide in tumors and inflammation [285]. The reported core–satellite nanoprobe was prepared by forming an amide bond between the ammonia-modified mesoporous silica-coated nano-gapped gold nanorods and the gold nanoparticles modified with 4-mercaptobenzoboric acid and D-(+)-galactose. A substrate and a peroxidase enzyme were loaded onto the mesoporous silica shell. Due to the plasmonic coupling between the external gold nanoparticles, a strong electromagnetic field was generated, that enhanced the SERS intensity of the modified Raman molecule and the optical absorption of the nanostructure in the second near-IR region. In the presence of hydrogen peroxide, the gold nanoparticles dissociated resulting in a decrease in the Raman signal at 2228 cm^−1^ but no change in the Raman signal at 1418 cm^−1^ inside the nanogap of gold nanorods. During the dissociation, the substrate that was loaded in the mesoporous silica shell converted to an oxidized form in the presence of hydrogen peroxide and the peroxidase enzyme (catalyst) resulting in a strong absorption at 750 nm with the absorption at 1250 nm of gold nanorods remaining nearly unchanged. Therefore, the ratiometric SERS and photoacoustic signals were used to detect hydrogen peroxide in vivo and in vitro quantitatively and selectively (Figure 16) [285].

### 4.8. MRI, EPRI, and PET Imaging

Although EPR oximetry is not currently used in clinical imaging, combined EPRI and PET scanners provide information about tumor microenvironments in preclinical studies [281]. In combined systems, EPRI is used as a gold standard to measure the partial pressure of oxygen in the environment to evaluate the accuracy of PET imaging. Additionally, the combination of PET and EPRI enables simultaneous measurement of intra- and extracellular components of tissue microenvironments, for example, glucose consumption rate, hypoxia, and acidosis. Furthermore, simultaneous scanning increases the efficiency of the imaging process which is important for biomedical applications [286].

**Figure 17:**
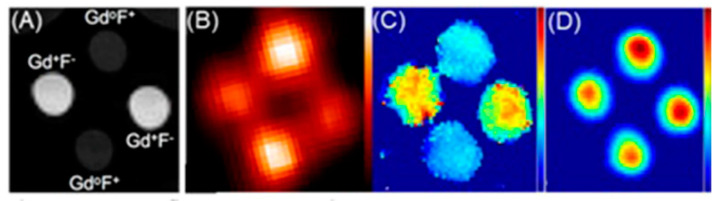
Imaging studies to detect hypoxia using *T*_1_-weighted MRI, PET, and EPRI. (**A**) *T*_1_-weighted MRI representing the presence and absence of Gd^III^-diethylenetriaminepentaacetic acid (defined in this example as Gd), and the presence or absence of ^18^F. The two variants of PET/EPRI imaging solution include: 1 mM of dFT, 1 mM of Gd and 25 µCi of **6** (defined as the Gd^+^F^−^ solution), and 1 mM of dFT and 49 µCi of **6** (defined as the Gd^o^F^+^ solution). (**B**) PET image intensity is related to the concentration of **6**. (**C**) EPR image of Lorentzian line width, and (**D**) EPR image of dFT concentration. Image intensity is related to dFT concentration. Adapted with permission of IOP Publishing, Ltd., from A combined positron emission tomography (PET)-electron paramagnetic resonance imaging (EPRI) system: initial evaluation of a prototype scanner, Tseytlin, M.; Stolin, A. V.; Guggilapu, P.; Bobko, A. A.; Khramtsov, V. V.; Tseytlin, O.; Raylman, R. R. *63*, **2018**; permission conveyed through Copyright Clearance Center, Inc. [287].

Raylman and co-workers reported a combined PET/EPRI prototype scanner for simultaneous acquisition of EPR and PET images of hypoxic samples that are created by removing endogenous oxygen by adding glucose and glucose oxidase [287]. The PET/EPRI scanner was used to detect oxygen-sensing EPR spin probe [per-deuterated ‘Finland’ trimethyl (trityl) radical (dFT)] and PET radiotracer **6** in samples (Figure 17). Because it is difficult to control oxygen concentration in the samples, gadolinium was also added to the solution to emulate the presence of oxygen. The study demonstrated that the highest concentration of gadolinium accurately corresponds to the samples with the lowest concentration of **6**. As demonstrated by spectral-linewidth in the EPR images of the samples the scanner has the ability to map oxygen concentration as simulated by the addition of gadolinium. The reported PET/EPRI scanner potentially enables the detection of cellular components of tumor microenvironments. Relatively low spatial resolution and detection sensitivity which are common limitations of the reported preclinical scanner potentially can be overcome using EPR-compatible PET systems with higher resolution and detection sensitivity than the reported scanner [287].

**Figure 18:**
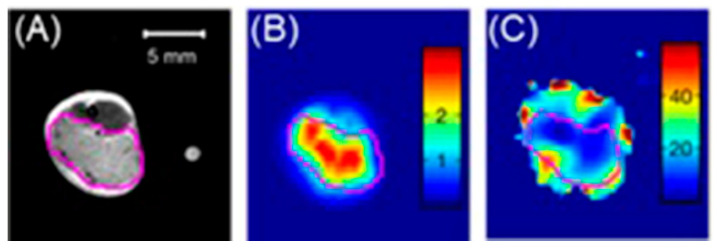
Tumor slice (Z = 2 mm) of a mouse (23.0 g) bearing a leg-born squamous cell carcinoma. (**A**) *T*_2_-weighted MR image defining the boundary of the tumor and marked by the pink line. (**B**) PET image of the same slice exhibits large uptake of **9** in the tumor, indicated by red color, and (**C**) pO_2_ image of the tumor slice acquired by EPRI. The hypoxic region is indicated by dark blue color. Adapted with permission of Royal Society of Chemistry, from Development of a PET/EPRI combined imaging system for assessing tumor hypoxia, Kim, H.; Epel, B.; Sundramoorthy, S.; Tsai, H. M.; Barth, E.; Gertsenshteyn, I.; Halpern, H.; Hua, Y.; Xie, Q.; Chen, C. T.; Kao, C. M. *16*, 03, **2021**; permission conveyed through Copyright Clearance Center, Inc. [288].

Kim and co-workers reported MRI-guided PET/EPRI scanner to probe oxygen concentration in hypoxic regions of mice bearing squamous cell carcinoma [288]. *T*_2_-weighted MRI was used as an anatomical reference to delineate the tumor region. Trityl spin probe and **9** were used to acquire EPRI and PET imaging data, respectively. Because of the reduced environment prevalent in hypoxic tumors, **9** tends to accumulate in hypoxic cells via covalent binding with macromolecules after the reduction in its nitro group. Furthermore, glutathione tends to bind with reduced **9** and accumulated in hypoxic regions. High uptake of **9** is shown in red, and uptake appears within the tumor boundary defined by *T*_2_-weighted MRI. Furthermore, the pO_2_ image from EPRI shows hypoxic regions (pO_2_ ≤ 10 Torr) by dark blue color in the image. Although there are some discrepancies observed between PET and EPRI images, some hypoxic regions are overlapped in both PET images using **9** and EPRI oxygen images (Figure 18) [288].

### 4.9. X-ray CT, MRI, and Photoacoustic Imaging

**Figure 19:**
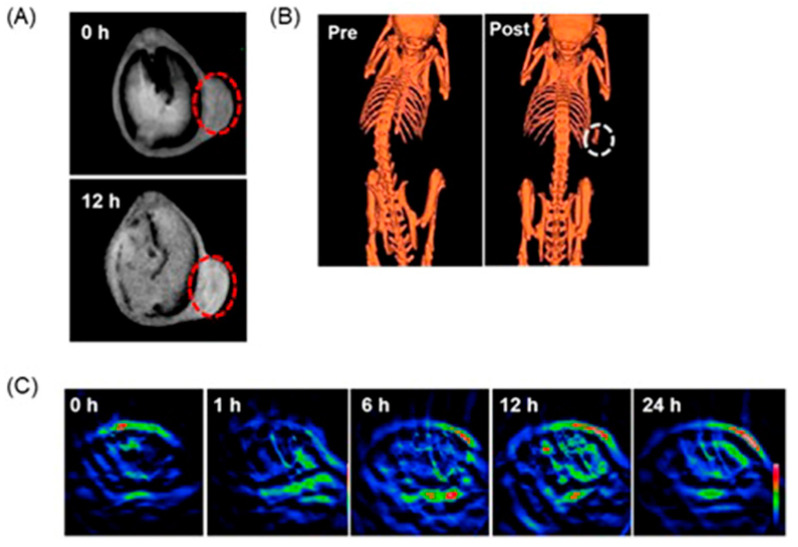
(**A**) *T*_1_-weighted MRI and (**B**) X-ray CT images of pre and 12 h post-injection of nanosponges into a 4T1 tumor-bearing mouse. (**C**) Multispectral optoacoustic images of 4T1 tumor injected with nanosponges. Dashed circles in (**A**,**B**) represent the tumor region. Adapted from *Biomaterials*, *220*, Wang, Y.; Song, S.; Lu, T.; Cheng, Y.; Song, Y.; Wang, S.; Tan, F.; Li, J.; Li, N. Oxygen-supplementing mesoporous polydopamine nanosponges with WS_2_ QDs-embedded for CT/MSOT/MR imaging and thermoradiotherapy of hypoxic cancer. 119405, Copyright 2019, with permission from Elsevier [290].

The combination of X-ray CT, MRI, and photoacoustic imaging enhances the precision and accuracy of the study of tumor environments [289]. The combined system is used to guide the staging of tumors. Li, Li, and co-workers reported mesoporous polydopamine nanosponges with embedded tungsten disulfide quantum dots and MnO_2_ that are responsive to hydrogen peroxide in an acidic tumor microenvironment [285]. These nanoplatforms were evaluated as trimodal contrast agents for X-ray CT, MRI, and multispectral photoacoustic imaging. Acidic and elevated hydrogen peroxide levels of tumor microenvironments of 4T1 carcinoma in mice were visualized using this nanoplatform (Figure 19). Because of the presence of acidic hydrogen peroxide, the entry of nanosponges that contained MnO_2_ to hypoxic cells enhances the contrast of *T*_1_-weighted images. Furthermore, embedded tungsten disulfide quantum dots provide the radiation enhancement effect in X-ray CT [290].

**Figure 20:**
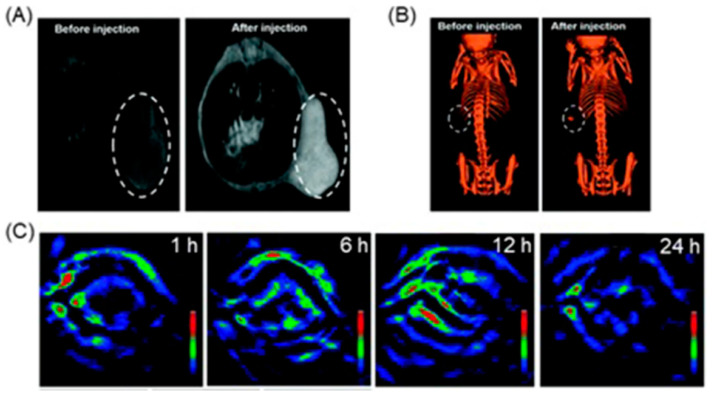
(**A**) *T*_1_-weighted images, (**B**) X-ray CT images, and (**C**) multispectral optoacoustic images of 4T1 tumor before and after injection with nanoparticles. Dash circles of (**A**,**B**) represent the tumor region. Adapted from Methods in Enzymology, *657*, Xu, S.; Shi, X.; Chu, C.; Liu, G. A TME-activated in situ nanogenerator for magnetic resonance/fluorescence/photoacoustic imaging, 145–156, Copyright 2021, with permission from Elsevier [291].

Yu, Li, and co-workers reported a nanoshell that contained mesoporous silica nanoparticles hybridized with MnO_2_ nanoparticles and gold nanoseeds [291]. The nanoshells enter hypoxic cells via enhanced permeability and retention effect. Upon entry, MnO_2_ was degraded and released Mn^II^ due to the presence of hydrogen peroxide and acidic milieu of hypoxic cells, resulting in an increase in signal intensities of both multispectral optoacoustic and *T*_1_-weighted MRI images (Figure 20) [291].

### 4.10. Fluorescence, Photoacoustic Imaging, and MRI

**Figure 21:**
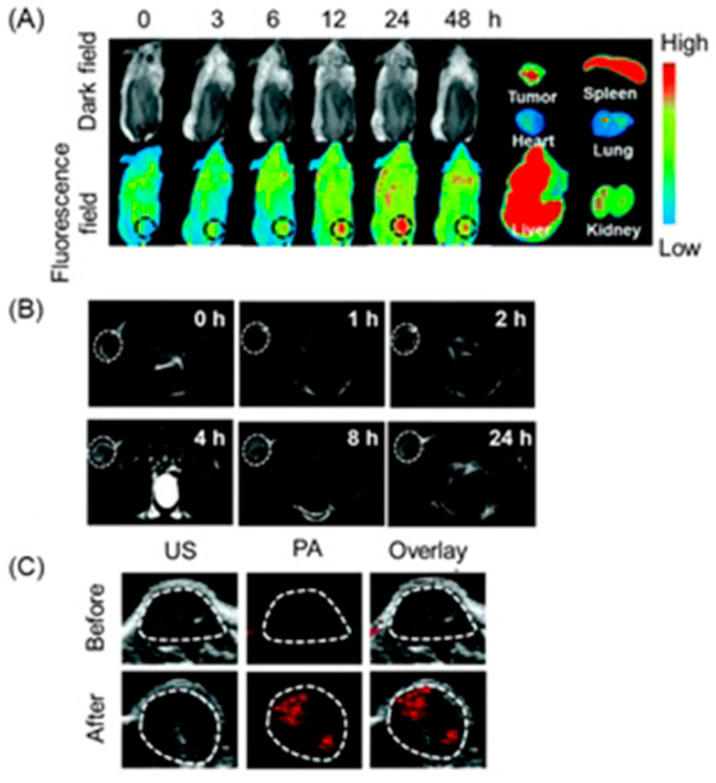
In vivo multimodal imaging. (**A**) Fluorescence images of mice bearing 4T1 tumors at 0, 3, 6, 12, 24, and 48 h after the injection of nanocomposite and fluorescence images of ex vivo organs and tumors excised at 24 h post-injection. (**B**) *T*_1_-weighted MRI of mice bearing 4T1 tumors at different time points after intravenous injection of nanocomposite, and (**C**) photoacoustic images in tumor region at 24 h before and after intravenous injection of nanocomposite. The white dashed lines enclose the tumor sites. Adapted with permission of the Royal Society of Chemistry, from A HMCuS@MnO_2_ nanocomplex responsive to multiple tumor environmental clues for photoacoustic/fluorescence/magnetic resonance trimodal imaging-guided and enhanced photothermal/photodynamic therapy, Li, Q.; Ren, J.; Chen, Q.; Liu, W.; Xu, Z.; Cao, Y.; Kang, Y.; Xue, P. *12*, 23, **2020**; permission conveyed through Copyright Clearance Center, Inc. [297].

Precise and reliable information about tumors can be obtained by combining fluorescence, photoacoustic imaging, and MRI [292,293,294,295,296]. With this combination, high sensitivity, deep tissue penetration, and high-resolution imaging are combined to generate a comprehensive picture of the hypoxic tumor region. Cao, Xue, and co-workers reported a hollow mesoporous copper sulfide and MnO_2_-based core–shell nanoplatform encapsulating a fluorophore, chlorin e6 (Ce6) as a highly efficient imaging and therapeutic agent for tumor hypoxia (Figure 21) [297]. Upon entering into the tumor via the enhanced permeability and retention effect, mild acidic conditions with elevated hydrogen peroxide levels cause degradation of MnO_2_. The subsequent release of Mn^II^ causes enhanced MRI signal and exposes encapsulated Ce6 that increases both fluorescence and photoacoustic signals. The acidic milieu of tumor microenvironments can be detected using this trimodal imaging strategy because of the combined properties of each imaging modality [297].

## 5. Dual-Mode Hypoxia-Responsive Probes

Information regarding the physiological status of tumors can be detected using molecular switches that are capable of differentiating hypoxic and normoxic conditions. Some multimodal imaging agents can be used to image tumors, and multiple imaging modalities can be used to detect these imaging agents. In this section, responsive imaging agents are described that are both (1) sensitive to hypoxia or hypoxia-induced pathological changes and (2) detectable using more than one imaging modality (Table 4). Advantages of the use of dual-mode hypoxia-responsive probes include the ability to overcome the concentration dependence of responsive probes, overcome the limitations of each method, and obtain information about tumor microenvironments.

### 5.1. Optical Imaging and MRI

**Figure 22:**
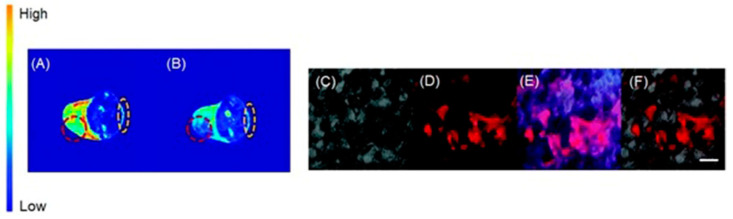
*T*_2_-weighted MRI images of subcutaneous tumor-bearing mouse (**A**) before, and (**B**) after injection of the nanoprobe. The tumor is highlighted in red dashed circle and normal tissues are highlighted in yellow dashed circle. A contrast enhancement (darkening) on the tumor site due to elevated levels of glutathione compared to normal tissues was observed. Time-gated luminescence images of the cryosection tumor from the tumor-bearing mouse injected with the nanoprobe. (**C**) bright field image, (**D**) time-gated luminescence image, (**E**) steady-state luminescence image, (**F**) merged image of (**C**,**D**). Scale bar: 10 µm. An intense red intracellular luminescence was observed in the cytoplasm of the cells, indicating that the nanoprobe was internalized into cells, and MnO_2_ nanosheets were reduced to Mn^II^ by intracellular glutathione, triggering luminescence recovery of the Eu^III^-containing complex. Adapted with permission of the Royal Society of Chemistry, from A dual-modal nanoprobe based on Eu(III) complex-MnO_2_ nanosheet nanocomposites for time-gated luminescence-magnetic resonance imaging of glutathione in vitro and in vivo, Song, B.; Shi, W.; Shi, W.; Qin, X.; Ma, H.; Tan, M.; Zhang, W.; Guo, L.; Yuan J. *11*, 14, **2019**; permission conveyed through Copyright Clearance Center, Inc. [302].

Among various types of optical imaging techniques, great attention has been placed on the use of time-gated luminescence with lanthanide-containing complexes [298,299,300,301]. These complexes have luminescence lifetimes that are longer than hundreds of microseconds, and consequently, they enable the ability to avoid short-lived (~ns) background fluorescence and scattered excitation in optical imaging, causing improvements in signal-to-noise ratios compared to steady-state experiments. Combined with MRI, which has high spatial resolution and nearly unlimited depth penetration, Song, Tan, and co-workers reported a time-gated luminescence and MRI dual-mode nanoprobe for glutathione detection, where glutathione is one of the key biomarkers of hypoxic tumors [302]. A β-diketone-Eu^III^-containing complex served as the luminescent material, and it was anchored to layered MnO_2_ nanosheets. The nanocomposite exhibited weak luminescence and MRI signal due to the quenching of both signals by MnO_2_. After peritumoral injection of the nanoprobe, both time-gated luminescence enhancement and *T*_2_-weighted MRI contrast enhancement were observed in the tumor region compared to control experiments in the healthy subcutaneous tissue of the same mouse (Figure 22). This observation was attributed to the presence of a large amount of glutathione that reduced MnO_2_ nanosheets into Mn^II^ ions. The study focused on increasing hypoxia detection accuracy and combining complementary advantages of fluorescence and MRI [302].

**Figure 23:**
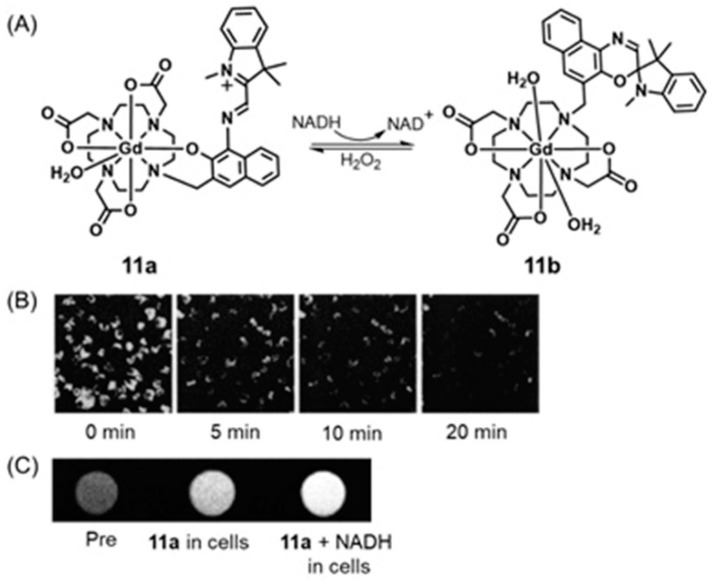
(**A**) The isomerization of complex **11** triggered by NADH and hydrogen peroxide. (**B**) Fluorescence images of P388D1 murine macrophages incubated with complex **11** obtained during the reaction with NADH (concentration of **11** = 0.103 mM, Gd/NADH molar ratio = 1:1). (**C**) *T*_1_-weighted MRI images of aqueous solutions of **11** immediately after addition of NADH (molar ratio between Gd^III^ and NADH = 1:1). Adapted with permission from Tu, C.; Nagao R.; Louie, A. Y. *Angewandte Chemie International Edition*, John Wiley and Sons [303].

Compared to redox-active luminophores, redox-active fluorophores are common, and some redox-responsive fluorophores can be detected using fluorimetry and MRI. Hypoxic cells contain elevated levels of reducing agents including NADH, and Louie and coworkers reported an NADH-responsive Gd^III^-containing complex that contains a fluorescent spironaphthoxazine moiety [303]. In polar solvents, spironaphthoxazine exhibits an acyclic merocyanine (open chain) form (**11a**) with phenoxide oxygen coordinated to Gd^III^. Because of the extended π-system, **11a** fluoresces in water with a quantum yield of 22%. Upon addition of NADH, fluorescence disappears within 30 min. Simultaneously, MR signal intensities of a series of mixtures of **11a** and NADH increase immediately after the addition of NADH. This response is caused by NADH-triggered isomerization of the merocyanine form into the spirocyclic isomer (**11b**) that removes phenoxide oxygen from the coordination sphere of Gd^III^, increasing the hydration number of Gd^III^ and, consequently, the relaxivity by 54% from 5.6 ± 0.4 to 8.6 ± 0.7 M^−1^ s^−1^. The disappearance of fluorescence and increase in relaxivity confirm the presence of elevated levels of NADH in hypoxic cells. From in vitro assessment using P388D1 murine macrophages (intracellular NADH level = 0.001 mM), images acquired immediately after the addition of NADH displayed fluorescence intensity that disappeared within 20 min. Furthermore, *T*_1_-weighted MRI experiments of P388D1 cells demonstrated that contrast enhancement occurred immediately upon the addition of NADH (Figure 23). Because the isomerization between the merocyanine and spirocyclic forms is reversible, the backward reaction in the presence of molecules including hydrogen peroxide, which is over-produced in hypoxic cells, is possible. However, overall, fluorescence and MRI signals responsive to NADH levels in cells are promising [303].

**Figure 24:**
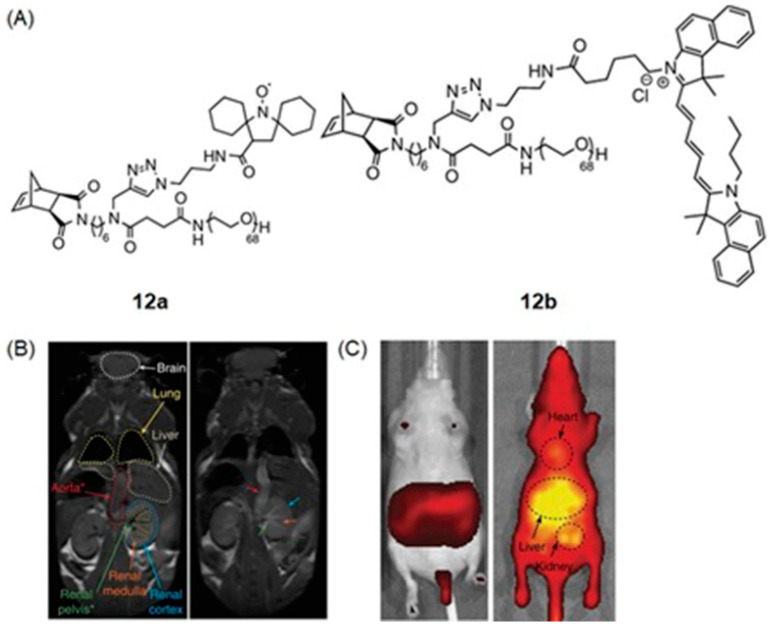
(**A**) Chemical structures of nitroxide-radical-containing organic molecule (**12a**) and cyanin5.5 near-IR fluorophore (**12b**) used to synthesize the polymer. (**B**) (left) *T*_1_-weighted MRI image of whole mouse before nanoparticle injection and (right) *T*_1_-weighted MRI image of the same mouse 30 min after nanoparticle injection. Some organs and tissues outlined for reference. The red asterisk indicates the structure labeled as aorta could also include inferior vena cava. The green asterisk indicates the structure labeled as renal pelvis could also include renal artery and renal vein. (**C**) Near-IR fluorescence imaging studies. (left) fluorescence image of whole mouse before nanoparticle injection. (right) fluorescence image of the same mouse in 30 min after injection. Maximum fluorescence is observed in the liver and kidneys, implying that the nitroxide radical-containing nanoparticles accumulate in the liver mostly in the reduced state. Adapted by permission from Springer Nature and Copyright Clearance Center: Springer Nature, *Nature Communications*, Redox-responsive branched-bottlebrush polymers for in vivo MRI and fluorescence imaging, Sowers, M. A.; McCombs, J. R.; Wang, Y.; Paletta, J. T.; Morton, S. W.; Dreaden, E. C.; Boska, M. D.; Ottaviani, M. F.; Hammond, P. T.; Rajca, A.; Johnson, J. A. Copyright 2014 [304].

Johnson and coworkers reported nitroxide radicals that can turn off MR signal and turn on fluorescence signal using redox-responsive branched-bottlebrush polymer nanoparticles (Figure 24) [304]. The polymer backbone is composed of multiple repeating units of nitroxide-radical-containing organic molecules (**12a**) and cyanin5.5 near-IR fluorophores (**12b**). Nitroxide radicals are quenchers of excited singlet states of fluorophores, and therefore, the fluorescence emission of **12b** is partially quenched in the nanoparticles. Because nitroxide contains a single unpaired electron, nitroxides catalytically shorten longitudinal relaxation times of nearby water protons to enhance the contrast of *T*_1_-weighted images. However, upon reacting with ascorbate or glutathione, nitroxides are reduced to diamagnetic hydroxylamines, leading to a decreased MRI contrast enhancement coupled with a 2- to 3.5-fold increase in fluorescence emission. Glutathione is over-expressed in hypoxic cells, and therefore, glutathione and other reducing agents including ascorbate mimic hypoxic tumors. In vivo, MRI and fluorescence imaging was performed by administering a solution of polymer nanoparticles in phosphate-buffered saline to mice (Figure 24) [304].

**Figure 25:**
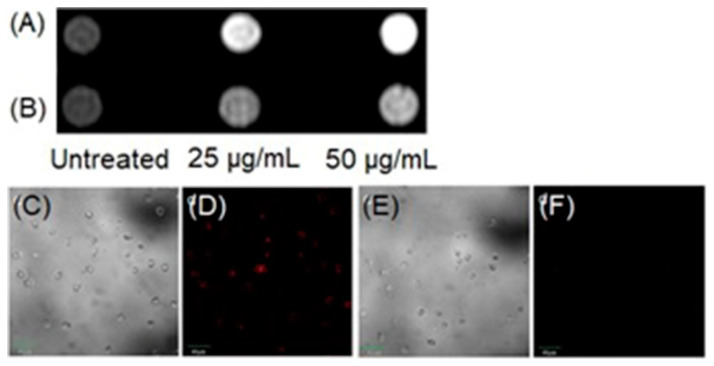
(**A**) *T*_1_-weighted MRI images of CCRF-CEM (hypoxic tumor cell line) cells and (**B**) Ramos (normoxic cell line) cells incubated with nanosheet at various concentrations in media for 3 h. Once MnO_2_ nanosheets are endocytosed and reduced by glutathione, Mn^II^ ions are produced. As a result, enhancement in *T*_1_-weighted MRI images was observed in hypoxic cells compared to normoxic cells. (**C**,**D**) Confocal microscopic images of CCRF-CEM cells treated with nanosheets. (**E**,**F**) Confocal microscopic images of Ramos cells treated with nanosheets. Red fluorescence was observed in the CCRF-CEM cells compared to Ramos cells after treated with nanosheets, indicating the fluorescence response of the nanosheets was activated in hypoxic cells. Adapted with permission from Zhao, Z.; Fan, H.; Zhou, G.; Bai, H.; Liang, H.; Wang, R.; Zhang, X.; Tan, W. Activatable fluorescence/MRI bimodal platform for tumor cell imaging via MnO_2_ nanosheet–aptamer nanoprobe. *J. Am. Chem. Soc.*, **2014**, *136*, 11220–11223. Copyright 2014 American Chemical Society [305].

Tan and co-workers reported a dual-activatable fluorescence and MRI bimodal glutathione-responsive nanoprobe that combined fluorescence cyanine5-labeled aptamers with redox-active MnO_2_ nanosheets (Figure 25) [305]. MnO_2_ efficiently quenched the fluorescence of cyanine5-labeled aptamers adsorbed onto the MnO_2_ nanosheets. The binding of aptamers to target cells caused partial recovery of fluorescence followed by endocytosis of the MnO_2_ nanosheets. Once endocytosed, the MnO_2_ nanosheets were reduced by intracellular glutathione, which is over-expressed in hypoxic cells, releasing Mn^II^ that enhanced contrast in *T*_1_-weighted MRI. The study enabled the combination of fluorescence and MRI for the precise detection of hypoxic cells with elevated glutathione levels (Figure 25) [305].

**Figure 26:**
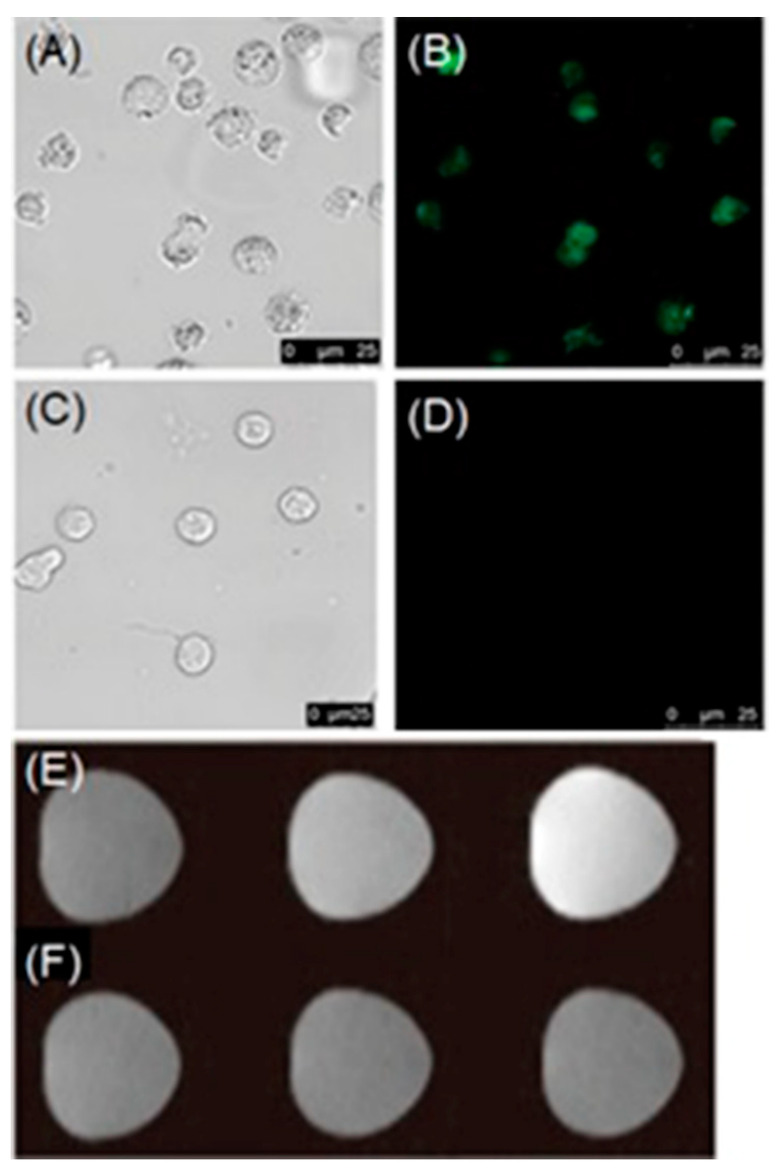
(**A**,**B**) Confocal microscopic images of CCRF-CEM cells and (**C**,**D**) Ramos cells treated with the nanoprobe, and the fluorescence signal amplified using rolling circle amplification. CCRF-CEM cells exhibited a green fluorescence compared to Ramos cells indicating that the fluorescence response of the nanoprobe was target cell-specific and activated by hypoxia. *T*_1_-weighted images of (**E**) CCRF-CEM cells and (**F**) Ramos cells incubated with the nanoprobe at various volumes (left to right: untreated, 30 µL, and 100 µL). CCRF-CEM cells exhibited more contrast enhancement in *T*_1_-weighted images compared to Ramos cells. These results were attributed to the target-cell recognition of the nanoprobe and reduction in MnO_2_ by intracellular glutathione. Adapted with permission [306].

Additionally using aptamers, Li and co-workers reported glutathione-activated aptamer nanoprobes with excellent sensitivity and selectivity [306]. In this case, rolling circle amplification technology improved the fluorescence signal of the probe. Target specificity of the probe was achieved by adsorbing fluorophore-labeled DNA aptamers onto MnO_2_ nanosheets. The fluorescence of the adsorbed aptamers was quenched by the MnO_2_ nanosheets. Reduction in MnO_2_ by intracellular glutathione released Mn^II^ that enhances contrast in *T*_1_-weighted MRI followed by initiating rolling circle amplification reaction in the presence of Phi29 DNA polymerase, circular DNA template, and deoxynucleotides (Figure 26). Long hairpin DNA probes carrying a large number of small hairpin DNA strands were produced in situ because of the action of rolling circle amplification. An amplified fluorescence signal enhances the sensitivity of the nanoprobe. The selectivity of the probe was demonstrated using various proteins, acids, and sugars that could provide interference for glutathione in cells, and those interferences were minimal [306].

**Figure 27:**
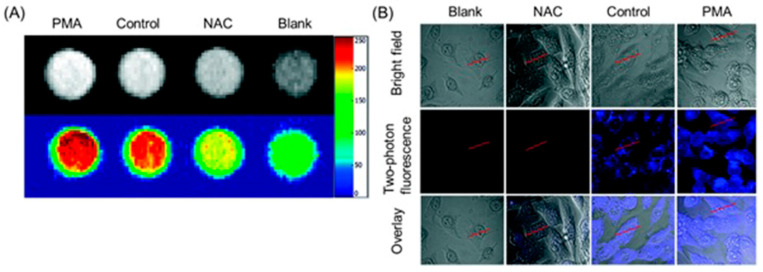
In vitro studies of MCF-7 cells incubated with TP-CQDs@MnO_2_ (375.0 µg mL^−1^). (**A**) *T*_1_-weighted MRI images of MCF-7 cells: PMA = cells incubated with TP-CQDs@MnO_2_ and phorbol-12-myristate-13-acetate; control = cells incubated with TP-CQDs@MnO_2_; NAC = cells incubated with TP-CQDs@MnO_2_ and N-acetyl-L-cystein (NAC); and blank = cells in buffer without adding TP-CQDs@MnO_2_. PMA is a hydrogen peroxide stimulant and NAC is a hydrogen peroxide scavenger. The brightest fluorescence of the two-photon images was observed with cells incubated with PMA and the weakest fluorescence was observed with cells incubated with NAC. The bright *T*_1_-weighted image of the control sample compared to the blank indicates that the TP-CQDs@MnO_2_ probe selectively visualizes endogenous hydrogen peroxide in MCF-7 cells. (**B**) Two-photon fluorescence images of MCF-7 cells incubated with TP-CQDs@MnO_2_ (375.0 µg mL^−1^). Blank = cells in buffer without adding TP-CQDs@MnO_2_; NAC = cells incubated with TP-CQDs@MnO_2_ and NAC; control = cells incubated with TP-CQDs@MnO_2_; PMA = cells incubated with TP-CQDs@MnO_2_ and PMA. Scale bars represent 20.0 µm. The fluorescence images show that the nanoprobe responds to endogenous hydrogen peroxide in a tumor microenvironment using two-photon fluorescence. Adapted with permission of the Royal Society of Chemistry, from Two-photon fluorescence and MR bio-imaging of endogenous H_2_O_2_ in the tumor microenvironment using a dual-mode nanoprobe. Zhong, H.; Yu, S.; Li, B.; He, K.; Li, D.; Wang, X.; Wu, Y.-X. *57*, 51, **2021**; permission conveyed through Copyright Clearance Center, Inc. [307].

Wu, Yu, Gan, and co-workers combined fluorescence and MRI to develop a dual-mode hydrogen peroxide-responsive bio-imaging nanoprobe based on two-photon carbon quantum dots for fluorescence imaging and MnO_2_ for MRI (TP-CQDs@MnO_2_) [307]. Compared to one-photon fluorescence, two-photon fluorescence imaging enables deeper tissue penetration and minimal background interference. The nanoprobe displayed high selectivity and spatial resolution with a low detection limit (1.425 pM) for hydrogen peroxide enabling differentiation between hydrogen peroxide levels in normal and hypoxic tissue. MnO_2_ nanosheets in the probe serve as a carrier and fluorescence quencher of the quantum dots. Upon endocytosis, MnO_2_ sheets degrade to Mn^II^ and subsequently enhance the contrast of *T*_1_-weighted MRI (Figure 27A). Additionally, upon degradation of MnO_2_ sheets, carbon quantum dots are released, increasing fluorescence intensity with two-photon fluorescence imaging (Figure 27B) [307].

**Figure 28:**
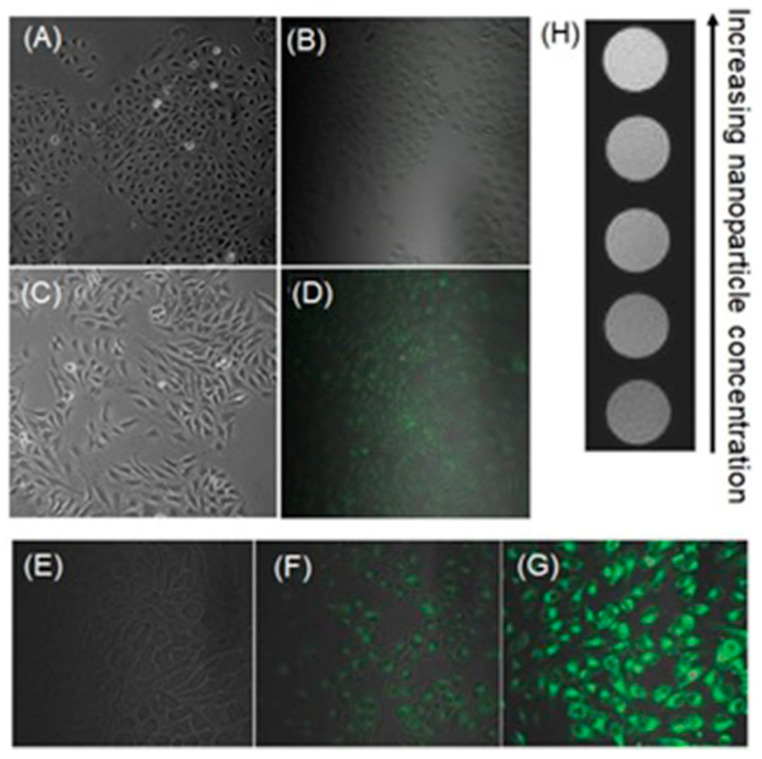
(**A**) Optical microscopy image and (**B**) fluorescence microscopy image of HaCaT cells. (**C**) Optical microscopy image and (**D**) fluorescence microscopy image of HeLa cells incubated with the nanoprobe. More green fluorescence was observed in HeLa cells compared to HaCaT cells, indicating greater accumulation and dissolution of the nanoprobe in acidic hypoxic cells. (**E**–**G**) Fluorescence microscopy images of HeLa cells treated with a MnO-based nanoprobe at 3, 12, and 24 h, respectively. Increase in fluorescence intensity was observed due to target-specific accumulation and dissolution of nanoprobe in hypoxic HeLa cells. (H) *T*_1_-weighted MRI images of HeLa cells incubated with the different nanoparticle concentrations (0–300 µg mL^−1^) for 24 h. Concentration-dependent MRI signal enhancement was observed as indicated by enhancement in in *T*_1_-weighted contrast of the samples containing HeLa cells. Adapted with permission from Hsu, B. Y. W.; Ng, M.; Tan, A.; Connell, J.; Roberts, T.; Lythgoe, M.; Zhang, Y.; Wong, S. Y.; Bhakoo, K.; Seifalian, A. M.; Li, X.; Wang, J. *Advanced Healthcare Materials*, John Wiley and Sons [308].

Li, Wang, and co-workers reported the use of fluorophore-bound Mn-based nanoparticles and nanosheets that have the ability to enhance *T*_1_-weighted contrast in MRI and fluorescence intensity under hypoxia-induced stimuli, including low pH and a reducing environment [308]. A hybrid silica nanoshell was prepared by encapsulating MnO nanoparticles and the fluorophore coumarin-545T. To enhance target specificity, the nanoprobes were conjugated with folic acid to target tumor cells that overexpress folate receptors. In this nanosystem, MnO nanoparticles serve as fluorescence quenchers of coumarin-545T. Upon receptor-mediated endocytosis into tumor cells, acidic conditions cause dissolution of MnO into Mn^II^ ions, resulting in contrast enhancement in *T*_1_-weighted MRI. Simultaneously, the fluorescence of coumarin-545T recovered. The presence of an acidic environment was confirmed using the nanoprobe that turns on both MRI and fluorescence signals. In vitro assessment of these nanoprobes was conducted using human dermal (HaCaT) and cervical cancer (HeLa) cell lines. Green fluorescence and contrast enhancement in *T*_1_-weighted MRI was observed in HeLa cells more than in HaCaT cells because of the acidic environment of the HeLa cells (Figure 28) [308].

**Figure 29:**
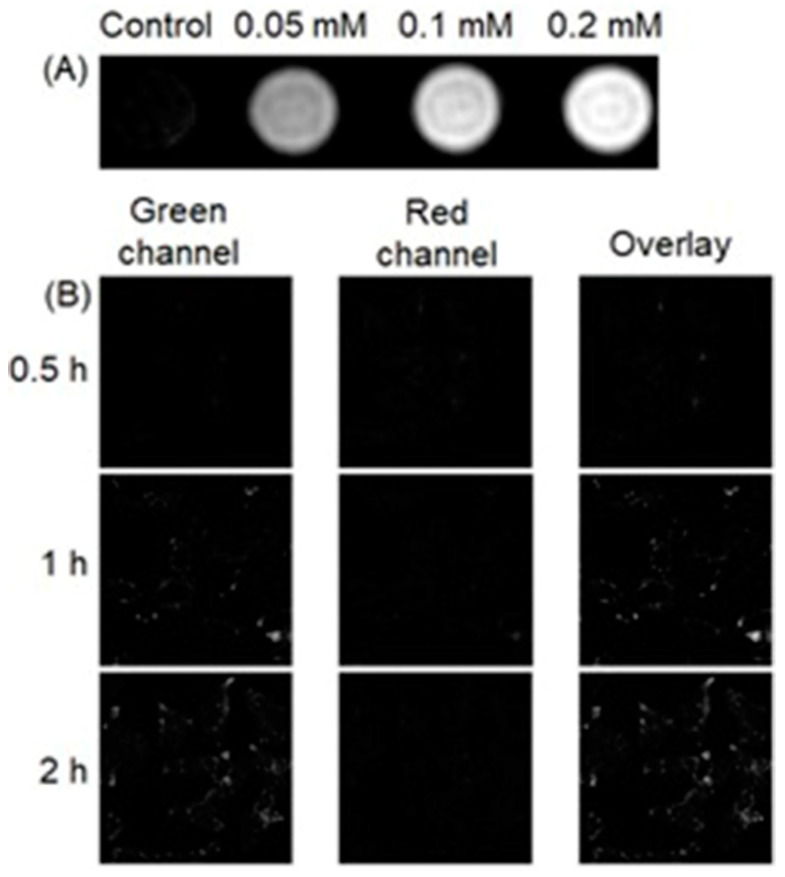
(**A**) *T*_1_-weighted spin-echo MRI images of HepG2 cells 4 h after incubation with core cross-linked polymeric micelles at varying Gd^III^ concentrations (0–0.2 mM). (**B**) Confocal fluorescence microscopy images of HepG2 cells incubated with cross-linked polymeric micelles. Adapted with permission from Hu, J.; Liu, T.; Zhang, G.; Jin, F.; Liu, S. *Macromolecular Rapid Communications*, John Wiley and Sons [309].

Intracellular transportation of nanoparticles is often associated with mildly acidic organelles including lysosomes, and this feature has been used to design pH-responsive polymeric nanocarriers for tumor-targeted delivery of imaging agents and therapeutic drugs. Jin, Liu, and co-workers reported core cross-linked polymeric micelles covalently labeled with Gd^III^-containing complexes for *T*_1_-weighted imaging and green-emitting fluorescent dyes [309]. Polymeric micelles are pH-responsive and exhibit mildly acidic pH-responsive signal enhancement for both fluorescence and MRI (Figure 29). Under neutral conditions, both MRI contrast agent and fluorophore are confined within the hydrophobic microenvironment of the micelles. Therefore, *T*_1_-weighted contrast enhancement is suppressed because of the restricted water exchange between the contrast agent and surrounding water. Additionally, fluorescence is quenched by deprotonated tertiary amine groups and high local fluorophore concentration. In mild acidic environments, the cross-linked micelle cores are rendered hydrophilic due to the protonation of tertiary amine residues. This phenomenon leads to recovery of both *T*_1_-weighted MRI signal and fluorescence due to efficient water exchange between contrast agent and surrounding water and inefficient mechanisms of fluorescence quenching. Because acidic pH is a characteristic feature of hypoxia, these probes are potential agents for imaging hypoxic tumor regions [309].

**Figure 30:**
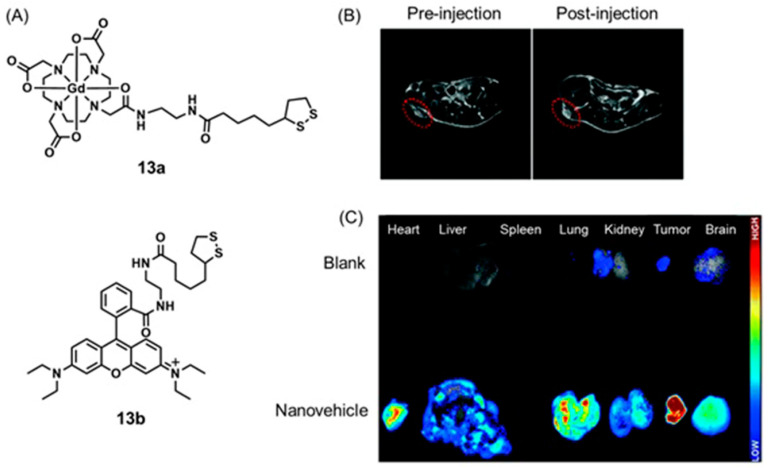
(**A**) Chemical structures of the **13a** and **13b**. (**B**) In vivo *T*_1_-weighted MRI images of U87 tumor-bearing nude mice before and after injection with nanovehicle. The red dashed circle indicates the tumor region. Larger signal intensity in the tumor site was observed post-injection in *T*_1_-weighted MRI compared to pre-injection, indicating acid-mediated dissolution of the nanovehicle and subsequent water exchange between surrounding and Gd^III^-containing complex. (**C**) The in vivo fluorescent images obtained using an excitation laser of 534 nm and an emission filter of 586 ± 20 nm. Adapted with permission of the Royal Society of Chemistry, from Multifunctional gold nanoparticles as smart nanovehicles with enhanced tumor-targeting abilities for intracellular pH mapping and in vivo MR/fluorescence imaging, Yu, K.-K.; Li, K.; Lu, C.-Y.; Xie, Y.-M.; Liu, Y.-H.; Zhou, Q.; Bao, J.-K.; Yu, X.-Q. *12*, 3, **2020**; permission conveyed through Copyright Clearance Center, Inc. [310].

Bao, Yu, and co-workers reported a series of responsive nanovehicles that can be used for intracellular pH mapping using *T*_1_-weighted MRI and fluorescence imaging [310]. Gd^III^-containing complex **13a** was used as the *T*_1_-weighted MRI component. Dual-sensitive functionalized nanoparticles were modified with a fluorescein derivative for basic pH measurements and rhodamine B derivative **13b** for acidic pH measurements. Tumor-targeting ability was obtained through the inclusion of cyclic arginine-glycine-aspartic acid peptide. All components were attached to gold nanoparticles via Au–S bonds. Quantitative pH measurements at the cellular level were performed using tumor-bearing mice. A signal enhancement in *T*_1_-weighted MRI and fluorescence signal in the tumor region was observed post-injection of functionalized nanoparticles. A significant enhancement of both signals was observed in tumor sites injected with nanoparticles that contained tumor-targeting peptides (Figure 30). Because acidic pH is an indicator of hypoxia in tumors, these nanovehicles are potentially suitable imaging agents for the detection of a variety of solid tumors [310].

**Figure 31:**
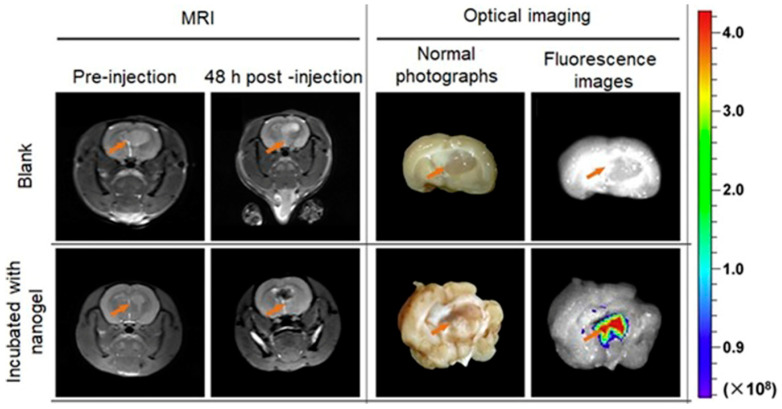
In vivo studies were performed with mice bearing gliomas (n = 9 in a group). Mice treated with saline (blank, **upper row**) and nanogel (**bottom row**). Images were acquired before and after tail vein injection (12 mg Fe/kg body weight). (Left) *T*_2_-weighted MRI images of gliomas before and 48 h post-injection. (Right) Normal photographs and ex vivo fluorescence images of gliomas at 48 h post-injection. *T*_2_-weighted MRI of brains acquired 6 h post-injection with nanogel exhibited contrast enhancement (darkening) in the tumor area. Consistently, intense fluorescence images were also observed in the brain 6 h post-injection. Adapted from *Biomaterials*, *34*, Jiang, L.; Zhou, Q.; Mu, K.; Xie, H.; Zhu, Y.; Zhu, W.; Zhao, Y.; Xu, H.; Yang, X. pH/temperature-sensitive magnetic nanogels conjugated with Cy5. 5-labeled lactoferrin for MR and fluorescence imaging of glioma in rats, 7418–7428, Copyright 2013, with permission from Elsevier [311].

Zhao, Yang, and co-workers reported a pH-sensitive magnetic nanogel conjugated with cyanin5.5-labeled lactoferrin for MRI and fluorescence imaging of tumors [311]. The nanogel changed hydrophilic and hydrophobic properties and size as a function of pH. Under physiological conditions, the nanogel was hydrophilic and swollen. In acidic environments of tumor tissues, the nanogel became hydrophobic and shrunken. In vivo studies of tumor-bearing mice injected with this nanogel demonstrated that pH-responsive structural changes facilitate the accumulation of the nanogel in tumor tissues and internalization by tumor cells (Figure 31) [311].

**Figure 32:**
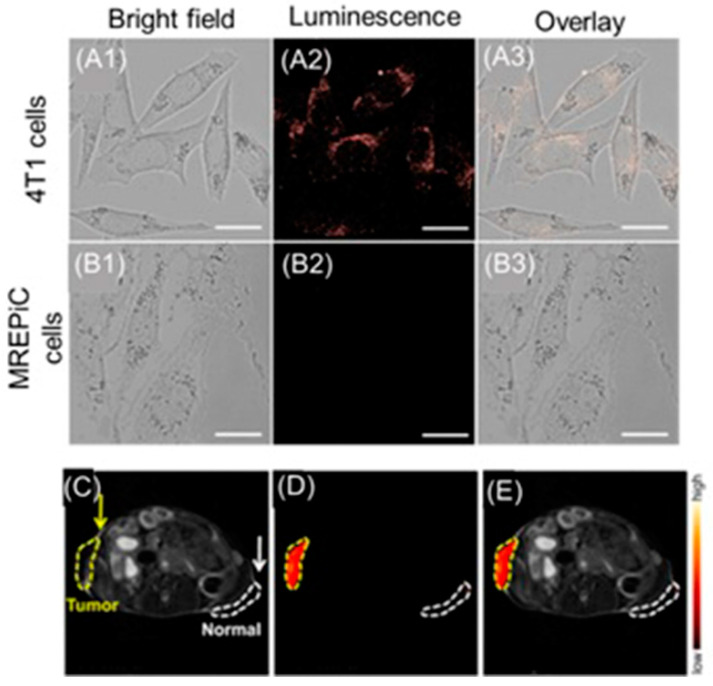
In vitro studies of hypoxic 4T1 cells, normoxic MREPiC cells, and in vivo studies of 4T1 tumor-bearing mice. Confocal laser scanning microscopy fluorescence images of (**A1**–**A3**) hypoxic 4T1 cells and (**B1**–**B3**) MREPiC normoxic cells incubated with nanoprobe for 4 h (400 µg mL^−1^). The excitation wavelength was 405 nm. All of the scale bars = 25 µm. Hypoxic cells incubated with the nanoprobe exhibited intense luminescence compared to normoxic cells. (**C**) ^1^H-, (**D**) ^19^F-MRI, and (**E**) overlay images of 4T1 tumor-bearing mice after intratumoral injection of the nanoprobe (20 mg mL^−1^, 150 µL). The same dose of nanoprobe was subcutaneously injected into normal tissue for comparison (white dashed lines denote the area). Yellow dashed lines mark the tumor site. Adapted with permission from Jiang, L.; Zhou, Q.; Mu, K.; Xie, H.; Zhu, Y.; Zhu, W.; Zhao, Y.; Xu, H.; Yang, X. pH/temperature-sensitive magnetic nanogels conjugated with Cy5.5-labeled lactoferrin for MR and fluorescence imaging of glioma in rats. *Biomaterials*, **2013**, *34*, 7418–7428. Copyright 2013 American Chemical Society [314].

Conventional ^1^H-MRI techniques for hypoxia imaging are often limited by low sensitivity due to large background signals [312]. ^19^F-MRI is a promising complementary technique to ^1^H-MRI that can overcome the issue of background interference. ^19^F-MRI has a negligible background signal in vivo [313]. Furthermore, the sensitivity of ^18^F-MRI can be improved by developing ^19^F-loaded nanoprobes. Therefore, the design and synthesis of dual-mode imaging probes for hypoxia based on ^19^F-MRI and fluorescence is an emerging area in biomedical applications. For example, Hu, Wang, and co-workers reported glutathione/pH dual-responsive nanoprobe capable of both fluorescence imaging in cells and ^19^F-MRI in deep tissue [314]. The nanoprobe was synthesized by encapsulating manganese oleate on the surface of fluorinated fluorescent quantum dots. Manganese oleate serves as an efficient quencher of both fluorescence and ^19^F-MRI signals. Upon entering hypoxic tumors that possess elevated levels of glutathione and mildly acidic pH, both fluorescence and ^19^F-MRI signals were turned on due to the degradation of Mn–O bonds within manganese oleate (Figure 32) [314].

**Figure 33:**
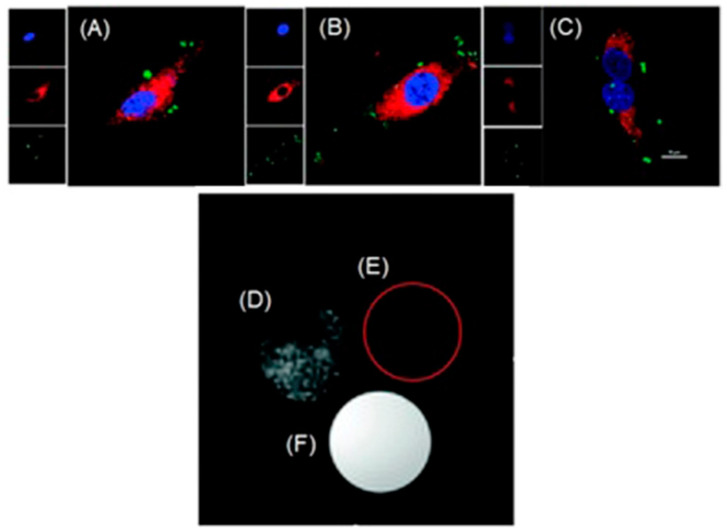
In vitro studies of the nanoparticles. Confocal fluorescence micrographs of nanoparticles incubated with (**A**) normal human lung fibroblast cells (MRC-5) for 3 h, (**B**) human lung cancer cells (A549) for 30 min, and (**C**) A549 cells for 3 h. Nuclei of all the cells are stained red, and nanoparticles are shown as green spots. These results indicate that the nanoparticles are selectively endocytosed by lung cancer cells (A549). In vitro studies. ^19^F-MRI images of fluorinated nanoparticles in (**D**) pH 7.4 buffer (phosphate-buffered saline), (**E**) A549 lung cancer cells, (**F**) pH 6.0 buffer (phosphate-buffered saline), where incubation was performed for 1 h. Field of view = 33 × 33 mm^2^. Adapted with permission of the Royal Society of Chemistry, from pH/temperature-sensitive magnetic nanogels conjugated with Cy5. 5-labeled lactoferrin for MR and fluorescence imaging of glioma in rats, Jiang, L.; Zhou, Q.; Mu, K.; Xie, H.; Zhu, Y.; Zhu, W.; Zhao, Y.; Xu, H.; Yang, X. *34*, 3, **2013**; permission conveyed through Copyright Clearance Center, Inc. [315].

One of the limitations associated with some nanoprobes is long retention times, which are undesirable in biomedical applications. However, in vivo studies demonstrated that the nanoprobe is a promising glutathione/pH-responsive agent that can selectively image tumor sites. Zhou and co-workers reported pH-responsive gold nanoparticle-capped fluorescein-functionalized mesoporous silica nanoparticles for ^19^F-MRI and fluorescent cancer cell imaging [315]. Under acidic conditions (pH < 6.0), the hydrazone linkers between gold nanoparticles and fluorescein-functionalized mesoporous silica nanoparticles are cleaved, removing the gold caps and releasing the entrapped ^19^F-containing contrast agent and fluorescein-functionalized mesoporous nanoparticles. Therefore, internalization into the acidic environment turns on both the ^19^F-MRI signal and fluorescence signal (Figure 33). Further, the gold nanoparticles were functionalized with folic acid to incorporate the tumor target ability of the nanoparticles [315].

**Figure 34:**
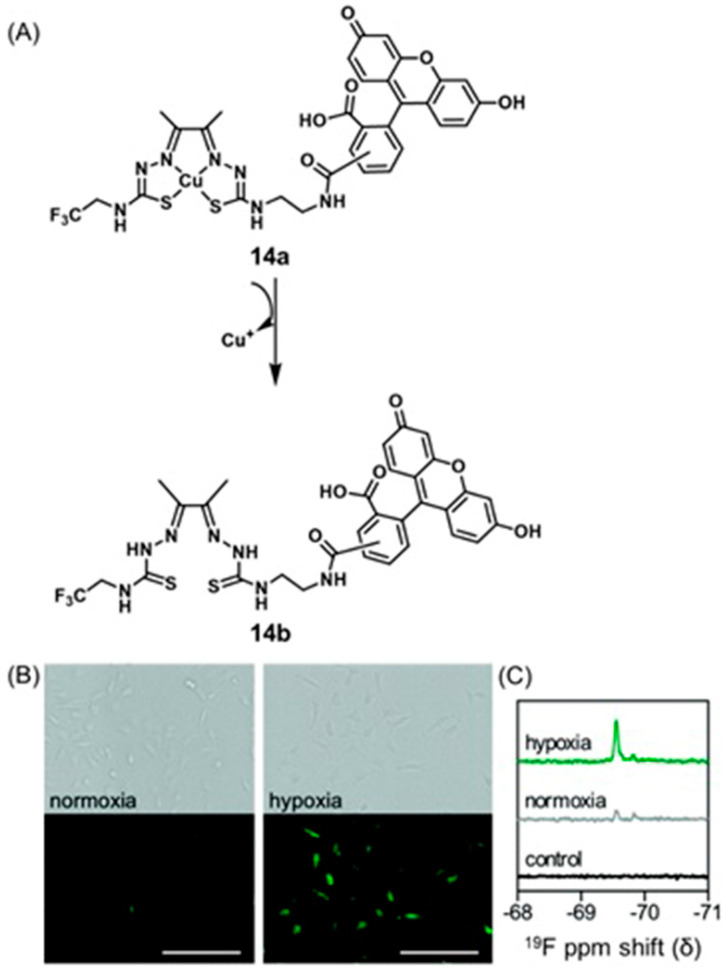
(**A**) Chemical structures and Cu^I^ elimination reaction that **14a** undergoes upon entering hypoxic cells. (**B**) Fluorescence images of normoxic [(left: brightfield (top) and fluorescence image (bottom)] and hypoxic [(right: brightfield (top) and fluorescence image (bottom)] of HeLa cervical cancer cells after 1 h incubation with **14a** (30 µM). Scale bar = 200 µm. (**C**) ^19^F-NMR spectra of lysed HeLa cells in anhydrous, *d*_6_-DMSO without incubating with **14a** (control, bottom), normoxic cells incubated with **14a** (60 µM) for 1 h (normoxic, middle), and hypoxic cells incubated with 14a (60 µM) for 1 h (hypoxia, top). Adapted with permission of the Royal Society of Chemistry, from A dual-responsive probe for detecting cellular hypoxia using ^19^F magnetic resonance and fluorescence. Kadakia, R. T.; Xie, D.; Martinez D. Jr.; Yu, M.; Que, E. L. *55*, 60, **2019**; permission conveyed through Copyright Clearance Center, Inc. [316].

Que and co-workers reported a dual-responsive ^19^F-MRI and fluorescence imaging probe for hypoxia detection [316]. The Cu^II^-containing probe, **14a**, exhibited no ^19^F-MRI signal and minimal fluorescence signal because of paramagnetic quenching. Upon entering hypoxic cells, the ability of the probe to enhance both modes of imaging was turned-on because of the reduction in Cu^II^ to Cu^I^. In vitro imaging studies using HeLa cells demonstrated that the imaging probe differentiates hypoxic and normoxic cells in both modalities (Figure 34) [316].

### 5.2. Fluorescence and Colorimetric Imaging

Fluorescence and colorimetric-based dual-mode hypoxia imaging are mainly targeted to confirm the hypoxia-relevant conditions or parameters. However, compared to other imaging techniques—including MRI, PET, and SPECT—fluorescence and colorimetric imaging require simple instrumentation and often can be performed at low costs. In these methods, organic dye molecules, quantum dots, and nanomaterials are usually used as chromophores.

**Figure 35:**
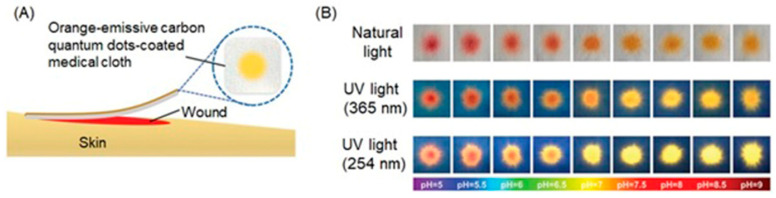
(**A**) Schematic representation of the medical cloth coated with quantum dots used in wound pH determination. (**B**) Photographs showing color under natural light and fluorescence images under UV light (excited at 365 and 254 nm) of the orange-emissive quantum dots in a buffer solution at different pH values. Adapted with permission from Yang, P.; Zhu, Z.; Zhang, T.; Zhang, W.; Chen, W.; Cao, Y.; Chen, M.; Zhou, X. *Small*, John Wiley and Sons [322].

Some chromophores respond to physical parameters, including pH and the reduction potential of the environment. Reports based on fluorescence and colorimetric detection of pH in nonbiological samples (for example, wastewater) are common [317,318]; yet, a few examples have been reported to detect biomolecules and the pH of physiological environments [319,320,321]. Physiological pH is important in detecting hypoxic tissues because pH tends to decrease as a consequence of reduced oxygen levels in cells [16,17]. Chen, Zhou, and co-workers reported orange-emissive carbon quantum dots for pH monitoring based on colorimetric and fluorescent imaging [322]. Orange-emissive carbon quantum dots were coated onto a medical cotton cloth and applied to chronic or infected wounds (where the pH is in the range of 5 to 9), and those cloths exhibited a response to pH variation via both fluorescence and visible colorimetric changes (Figure 35) [322]. Wounds occur due to various medical conditions including diabetes and hypoxic tumors [323,324]. Therefore, dual-mode monitoring of wound pH can be used as part of a diagnostic strategy for some chronic diseases.

Elevated levels of glutathione and hydrogen peroxide are critical factors in hypoxic tissues [77,100]. Therefore, agents that are responsive to those chemicals can be used in hypoxia detection. Hasan and co-workers reported fluorescent nitrogen–sulfur dual-doped carbon quantum dots for colorimetric detection of hydrogen peroxide and glutathione in human blood serum [325]. This platform detected hydrogen peroxide colorimetrically with a detection limit of 0.004 mM. Furthermore, glutathione was detected with this platform up to 0.07 µM using both colorimetric and fluorescence assays [325].

### 5.3. SERS and Fluorescence Imaging

Despite high sensitivity and fast imaging speed, applications of single fluorescent labeling techniques at the cellular level are limited because of photobleaching [326,327]. On the other hand, SERS possesses high photostability and little photobleaching compared to fluorescence imaging [328,329]. SERS also possesses the capability of recognizing different cell types [329]. However, in spite of its imaging properties, SERS suffers from slow detection speeds that limit the rapid recognition of pathological markers expressed in cancer cells [330,331]. Therefore, in modern cancer research, there is a growing interest to combine SERS and fluorescence imaging to enable fast and reliable sensing of multiple cancer cell types [332,333,334]. As a consequence, preclinical studies based on chromophores combined with metal nanoparticles have resulted in the visualization of biomolecules and physiological properties [335,336,337].

**Figure 36:**
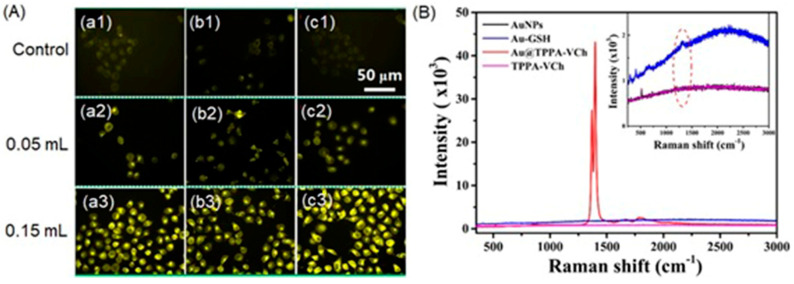
(**A**) Fluorescence images of parallel samples (a, b, and c) of HepG2 cells incubated with functionalized gold nanoparticles (10 µg mL^−1^) for 20 min. Excitation and emission wavelengths are 438 and 613 nm, respectively, in different volumes of glutathione. (**B**) SERS intensity of gold nanoparticles (AuNPs), immunoassay probe (TPPA-VCh), functionalized gold nanoparticle with immunoassay probe (Au@TPPA-VCh), and gold nanoparticles functionalized with glutathione (Au@GSH). The inset shows the enlarged spectra of AuNPs, AuGSH, and TPPA-VCh, Excitation wavelength = 438 nm; exposure time = 10 s. Adapted from *Spectrochimica Acta Part A: Molecular and Biomolecular Spectroscopy*, *223*, Yao, W.; Chang, L.; Yin, W.; Wang, T.; Yang, Y.; Yin, P.; Yang, M.; Ma, Y.; Qin, Y.; Ma, H. One immunoassay probe makes SERS and fluorescence two readout signals: Rapid imaging and determination of intracellular glutathione levels, 117303, Copyright 2019, with permission from Elsevier [340].

Gold nanoparticles have a strong binding affinity to sulfur-containing compounds including thiols and organosulfur compounds [338,339]. The surface of gold nanoparticles can be modified using nitrogen- or oxygen-containing chromophores to observe changes in SERS and fluorescence signals in response to glutathione levels in cells because glutathione can preferentially bind with gold nanoparticles and displace oxygen- or nitrogen-containing compounds. Yao, Ma, and co-workers reported a SERS-fluorescence dual-mode imaging system to probe intracellular glutathione levels [340]. A luminophore was synthesized by combining tris-(4-(pyridin-4-yl)-phenyl)amine and vinyl-benzylchloride, and the luminophore were loaded onto the surface of gold nanoparticles via Au–N bonds. As loaded, the fluorescence energy of the luminophore transferred to gold nanoparticles and consequently, quenched the fluorescence signal. Additionally, both gold nanoparticles and luminophores individually exhibited no Raman signals in the range of 250 to 3000 cm^−1^. However, gold nanoparticles loaded with luminophore exhibited a SERS effect. Upon entering hypoxic cells, Raman signals weakened and fluorescence increased because Au–S bonds with glutathione are stronger than Au–N bonds formed with the luminophore (Figure 36). This study demonstrated the complementary advantages of SERS and fluorescence to detect glutathione, which is produced at elevated levels in hypoxic cells [340].

**Figure 37:**
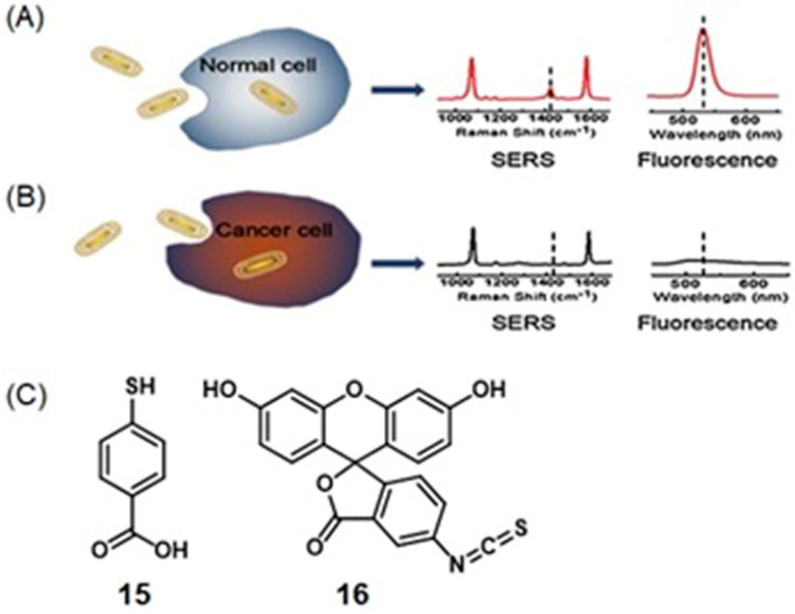
The sensing mechanism of functionalized gold nanoprobe in cells. (**A**) Under normoxic conditions. Internalized probe results in a carbon stretching band in the SERS spectrum at 1423 cm^−1^ and a strong absorption peak in fluorescence spectrum. (**B**) Upon entering into hypoxic cells, both signals disappear. (**C**) Chemical structures of **15** and **16**. Adapted from *Colloids and Surfaces A: Physicochemical and Engineering Aspects*, *562*, Yang, G.; Zhang, Q.; Liang, Y.; Liu, H.; Qu, L.-L.; Li, H. Fluorescence-SERS dual-signal probes for pH sensing in live cells, 289–295, Copyright 2019, with permission from Elsevier [341].

In addition to favorable binding affinities of SERS substrates, some fluorescence tags can be structurally modified to alter their fluorescence as a function of pH. Qu, Li, and co-workers demonstrated the pH sensing ability of fluorescein isothiocyanate combined with thiol-functionalized (**15**) gold nanorods using fluorescence and SERS imaging [341]. Fluorescein isothiocyanate, **16**, exhibits isoforms in an aqueous solution that change fluorescence as a function of pH. At pH 3, the fluorescence of fluorescein isothiocyanate is completely quenched, and fluorescence intensity increases with increasing pH (Figure 37). Additionally, the SERS spectra of the functionalized gold nanorods exhibit a characteristic spectral band at 1423 cm^−1^ (CO_2_^−^ expansion) that is sensitive to pH. In acidic media, the CO_2_^−^ stretching mode decreases because of the predominant existence of CO_2_H. When pH increases, CO_2_^−^ is predominantly present, causing an increase in the CO_2_^−^ stretching band at 1423 cm^−1^ (Figure 37). In vitro studies showed a fluorescence signal of tumor cells incubated with the nanoprobe compared to healthy cells. A characteristic spectroscopic peak at 1423 cm^−1^ of tumor cells incubated with nanoprobe is lower than that of healthy cells, indicating that normal cells possess a higher pH compared to tumor cells [341].

**Figure 38:**
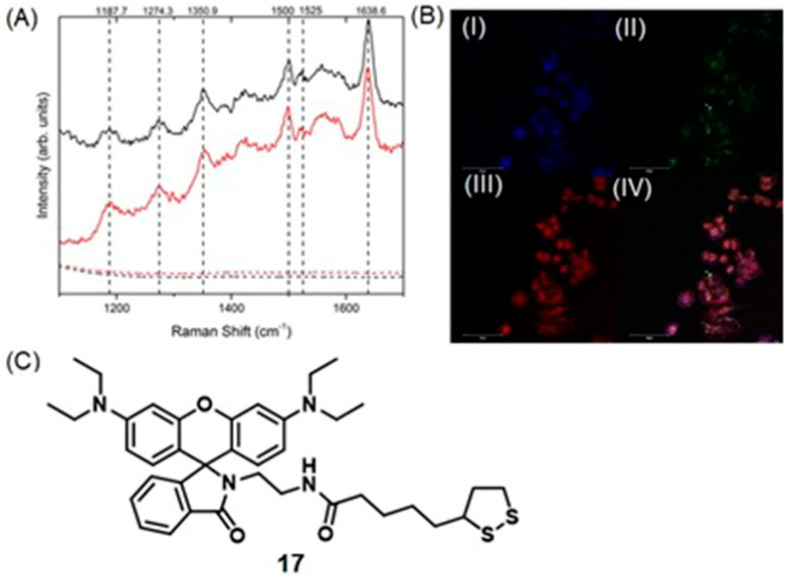
(**A**) Raman spectra of **17** (dashed lines) and gold nanoparticles functionalized with **17** (solid lines) at low pH (red) and high pH (black). (**B**) Co-localization of gold nanoparticles functionalized with **17** in A2780 cells visualized via confocal microscopy. (I) Lysotracker staining, (II) gold nanoparticles functionalized with **17**, (III) Mitotracker staining, and (IV) overlay (298 K). (**C**) Chemical structure of **17**. Adapted with permission from Eling, C. J.; Price, T. W.; Marshall, A. R. L.; Viscomi, F. N.; Robinson, P.; Firth, G.; Adawi, A. M.; Bouillard, J.-S. G.; Stasiuk, G. J. *ChemPlusChem*, John Wiley and Sons [344].

Imaging probes can be used to image organelles within cancer cells that exhibit hypoxia-induced pathological conditions. For example, the mitochondrial membrane in cancer cells exhibits increased negative potential compared to healthy cells; therefore, cationic species are prone to accumulate within mitochondria in cancer cells [342,343]. Adawi, Bouillard, Stasiuk, and co-workers developed a pH-sensitive fluorescence probe using gold nanoparticles functionalized with a rhodamine thiol derivative for mitochondrial imaging (Figure 38) [344]. Cationic derivatives of rhodamine possess a high affinity toward mitochondria in cancer cells, and consequently, rhodamine-conjugated imaging agents are known to concentrate within cancer cells. The fluorescence emission intensity of the rhodamine-conjugated functionalized (**17**) gold nanoparticles increases with increasing pH. This pH response is based on the p*K*_a_ of rhodamine changing from 4.59 to 6.62 upon conjugation with gold nanoparticles. This greater p*K*_a_ is good for biological pH imaging between pH 6.5 and 7.4. The Raman spectra of rhodamine-functionalized gold nanoparticles exhibit clear enhancement of Raman signals compared to rhodamine (Figure 38). This study incorporated the advantages of fluorescence and SERS to precisely monitor mitochondrial pH in cancer cells (Figure 38) [344].

**Figure 39:**
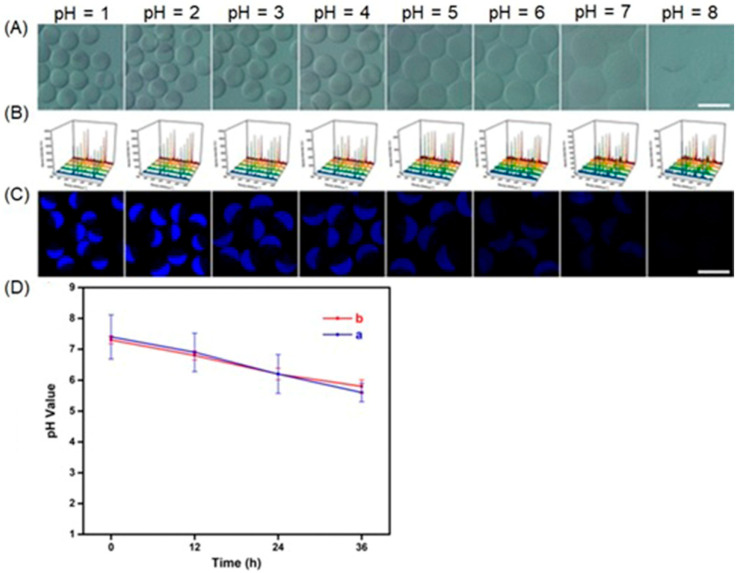
In vitro studies of Janus microgels. (**A**) Optical images of Janus microgels in buffer solutions with pH from 1.0 to 8.0 (scale bar = 200 µm). (**B**) SERS spectra of SERS hemispheres in buffer solutions with pH from 1.0 to 8.0. (**C**) Confocal laser scanning microscopy fluorescence images of Janus microgels in buffer solution with pH from 1.0 to 8.0 (scale bar = 200 µm). (**D**) Determination of pH values based on (blue, a) Raman intensity ratio of the peaks at 1395 and 1077 cm^−1^ and (red, b) fluorescence intensity. Adapted with permission from Yue, S.; Sun, X.; Wang, N.; Wang, Y.; Wang, Y.; Xu, Z.; Chen, M.; Wang, J. SERS–fluorescence dual-mode pH-sensing method based on Janus microparticles. *ACS Appl. Mater. Interfaces*, **2017**, *9*, 39699–39707, Copyright 2017 American Chemical Society [345].

One limitation of traditional SERS-based pH sensing probes is the inability to measure the pH of acidic (pH < 5.5) media due to aggregation. Xu and co-workers reported a novel pH-sensing method based on pH-sensitive Janus microgels encapsulating 4-mercaptobenzoic acid-labeled gold nanoparticles and carbon dots [345]. Combining the advantages of high specificity offered by SERS and the fast imaging ability of fluorescence imaging, this dual-mode method detected tumor cells with extracellular pH 5.5–6.0. The pH decreases in MCF-7 culture media were monitored as a function of time. The pH of the culture media dropped to 5.5–6.0 when the cells were cultured for 36 h (Figure 39). Acidic pH is one of the earliest signs of hypoxic tumors; therefore, it is important to monitor pH to enable the early diagnosis of tumors [345].

### 5.4. SERS and Colorimetric Imaging

Compared to other imaging techniques, colorimetry is simple and inexpensive. Combining SERS with colorimetry into high-specificity, dual-mode systems enable accurate detection of a wide range of parameters related to hypoxia.

Lee and co-workers reported the use of colorimetric and SERS dual-mode sensing for selective and sensitive detection of endogenous H_2_S in live prostate cancer cells [346]. H_2_S is a gaseous signaling molecule that plays an important role in biological processes in living organisms including tissue homeostasis and cardiovascular pathophysiology. Abnormal levels of H_2_S are related to various medical conditions including cancer. Therefore, the detection of H_2_S levels in cells enables the detection of hypoxia. The study reported by Lee and co-workers is based on a silver nanoplate-coated paper assay to detect concentrations of H_2_S below 100 nM. This method also possesses the ability to measure concentrations of H_2_S in the nanomolar or micromolar ranges depending on if colorimetry or SERS is used for detection [346].

**Figure 40:**
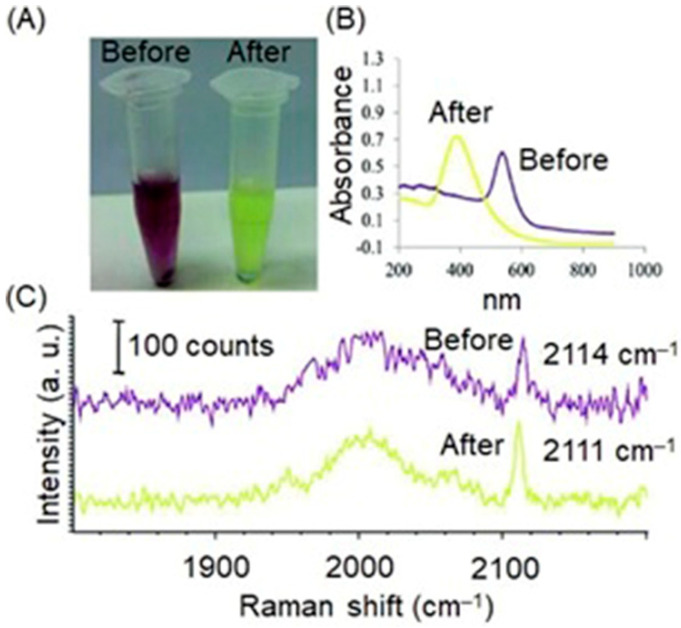
(**A**) Photographs of the probe (purple solution) and after addition of cysteine to the probe (yellow solution). (**B**) UV–visible spectra and (**C**) SERS spectra of 1 mM probe before (purple) and after (yellow) the addition of 1 mM cysteine. Adapted with permission of the Royal Society of Chemistry, from Dual-mode biosensor combining transition metal carbonyl-based SERS and colorimetric readout for thiol detection, Lin, D.; Zhou, J.; Yu, Y.; Chen, W.; Liao, P.-H.; Huang, H.; Kong, K. V. *11*, 41, **2019**; permission conveyed through Copyright Clearance Center, Inc. [347].

Huang, Kong, and co-workers reported a dual-mode biosensor combining transition metal carbonyl-based SERS and a colorimetric readout for thiol detection [342]. The probe was developed using an unsaturated triosmium carbonyl cluster [Os_3_(CO)_10_(µ-H)_2_] that undergoes color changes and shifts in the SERS spectrum carbonyl stretching vibration in the presence of thiol (Figure 40). The probe has a detection limit of 0.1 µM by SERS [347].

### 5.5. Fluorescence and Photoacoustic Imaging

**Figure 41:**
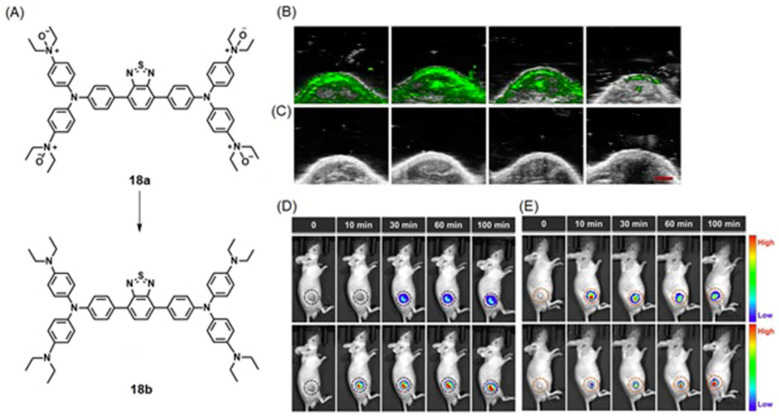
(**A**) Chemical structures of **18a** and **18b**. Time-dependent in vivo photoacoustic images of the tumor region in mice after tail vein injection of (**B**) nanoparticles of **18a** (200 µL, 1 mg mL^−1^) and (**C**) saline (200 µL) as control. (Left to right) Images were obtained after 6, 24, 48, and 72 h post-injection of nanoparticles. Scale bar = 2 mm. A strong photoacoustic signal was detected 6 h post-injection demonstrating the responsiveness of **18a** to hypoxia. (**D**,**E**) Time-lapse near-IR fluorescence images of mice before (0 min) and after (10, 30, 60, and 100 min) being intratumorally injected with (**D**) **18a** nanoparticles and (**E**) **18b** nanoparticles (50 µL, 1 mg mL^−1^). Top panels are the fluorescence signals collected upon excitation at 500 nm, and the bottom panels are the fluorescence signals collected upon excitation at 570 nm. The fluorescence in mice injected with **18a** decreases overtime with excitation at 500 nm, whereas fluorescence with 570 nm excitation increased overtime. The changes indicate the reduction in **18a** upon entering hypoxic cells. Because **18b** is not sensitive to hypoxia, fluorescence remained constant within 100 min post-injection regardless of the excitation wavelength. Adapted with permission [348].

Combining fluorescence imaging and photoacoustic imaging has led to the synthesis of dual-mode probes for imaging hypoxic tumor microenvironments. Li, Wang, Tang, and co-workers reported hypoxia-activated probe **18a** for near-IR fluorescence and photoacoustic dual-mode imaging of tumors [348]. Upon entering reduced environments, that are characteristic of hypoxic tumors, **18a** is reduced to **18b**. Due to aggregation-induced emission and the twisted intramolecular charge transfer features of **18b**, the probe possesses near-IR fluorescence and photoacoustic imaging properties that are useful in tumor imaging (Figure 41) [348].

**Figure 42:**
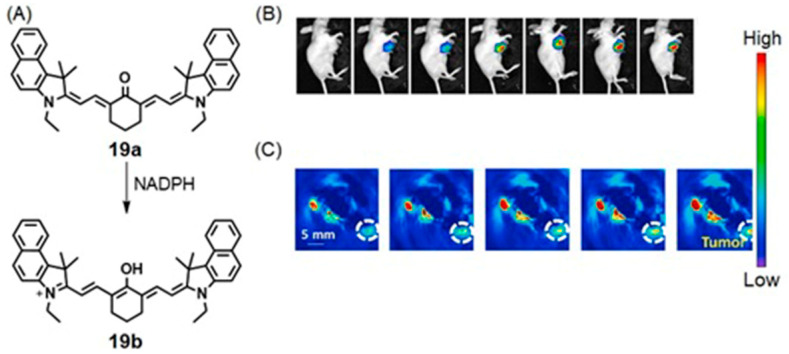
In vivo imaging studies of **19**. (**A**) Chemical structures and conversion of **19a** into **19b** in the presence of NADPH. (**B**) Time-dependent near-IR fluorescence images of tumor-bearing mice injected with **19a** [images were collected at (left to right) 0, 5, 30, 60, 90, 120, and 150 min]. (**C**) Time-dependent photoacoustic images of tumor-bearing mice injected with **19a** [images were collected at (left to right) 0, 30, 60, 90, and 120 min]. The tumor site is indicated by white dashed circles. Relative fluorescence intensity and photoacoustic intensity of images increase with time due to the formation of **19b**. Adapted from *Biomaterials*, *271*, Tian, Y.; Jiang, W.-L.; Wang, W.-X.; Mao, G.-J.; Li, Y.; Li, C.-Y. NAD(P)H-triggered probe for dual-modal imaging during energy metabolism and novel strategy of enhanced photothermal therapy in tumor, 120736, Copyright 2021, with permission from Elsevier [349].

The reduced coenzymes, NADPH, and NADH are important metabolic products and are closely associated with the occurrence and development of cancer [96]. Therefore, it is important to detect these reduced coenzymes to improve cancer detection and treatment. Li and co-workers reported a near-IR fluorescence and photoacoustic dual-modal multifunctional probe that consisted of **19a** [349]. The native version of the probe is not fluorescent in the near-IR region of the electromagnetic spectrum. However, upon reacting with NADPH, near-IR fluorescence was observed due to the formation of the conjugated structure **19b** (Figure 42). Furthermore, the probe exhibited photoacoustic signals upon reacting with NADPH. The probe was used to monitor NADPH during energy metabolism in tumor-bearing mice by fluorescence and photoacoustic dual-mode imaging (Figure 42) [349].

**Figure 43:**
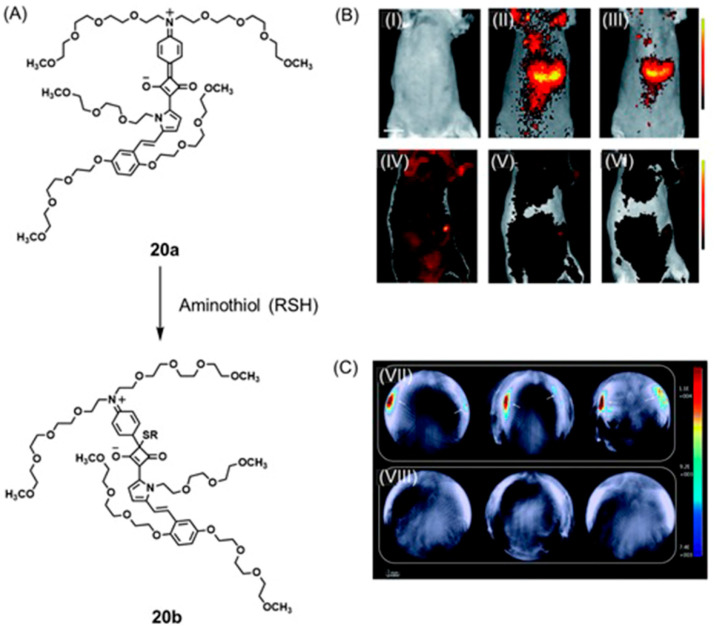
(**A**) Chemical structures and conversion of **20a** to **20b** in the presence of aminothiols. (**B**) In vivo fluorescence reflectance images from dorsal side of a seven-week-old fasting mouse (top panels) and post-food mouse (bottom panels). (I) pre-injected with **20a** in a fasting female mouse at 675 ± 15 nm excitation and 700 ± 10 nm emission. (II) and (III) images were recorded after 15 and 40 min, respectively, of injection of **20a** under fasting conditions. Images of mouse in post-food conditions were recorded upon excitation of 675 ± 15 nm and 700 ± 10 nm emission. Images were recorded at (IV) 5, (V) 15, and (VI) 40 min. Scale bar = 1.5 mm. Under fasting conditions, low levels of aminothiols are present in blood. Therefore, strong near-IR fluorescence and photoacoustic signals can be observed during fasting conditions. After food, high levels of aminothiols in blood are observed. Hence, both near-IR and fluorescence signals become weakened. Based on (II) and (III), a strong fluorescence emission indicates effective distribution of **20a** in blood and its accumulation in the abdominal area. After feeding, images (IV), (V), and (VI) exhibit weak fluorescence, indicating that thiol production after food was high and as-generated thiols reacted with **20a**. (**C**) Single wavelength multispectral optoacoustic images of a live mouse at 680 nm. (VII) Anatomy of the fasting mouse at 40 min post-injection of **20a**. (VIII) Anatomy of the mouse under post-food conditions at 40 min post-injection of **20a**. White arrows indicate fluorescence signals from **20a**. Adapted from Ref. [351] with permission from the Royal Society of Chemistry.

Because cellular thiols are essential biomolecules that play a major role in redox homeostasis and cellular growth, abnormal levels of thiols are closely associated with various conditions including oxidative stress [96]. Optical techniques are suitable for the detection of aminothiols because of their sensitivity, simple operation techniques, and low costs [350]. Ajayaghosh, Zhao, and co-workers reported an unsymmetrical near-IR squaraine dye, **20a**, as a contrast agent for real-time monitoring of aminothiol levels in live animals using near-IR fluorescence and photoacoustic bimodal imaging [351]. Squaraine exhibits a narrow absorption band at 680 nm that generates a photoacoustic signal and a strong near-IR emission at 700 nm. However, in the presence of thiols in biological systems, both near-IR and photoacoustic signals disappear. In vitro and in vivo studies were performed using mice with severe immune deficiency and demonstrated the applications of the squaraine dye in biomedical applications (Figure 43) [351].

**Figure 44:**
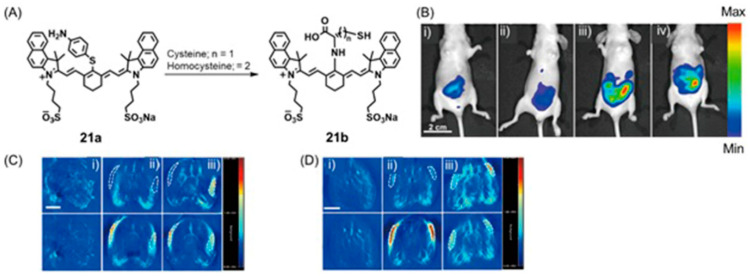
(**A**) Conversion of **21a** to **21b** in the presence of cysteine and homocysteine. (**B**) Fluorescence images of mice after intraperitoneal injection with **21a**. (I) images were collected 20 min after injection (50 µM, 100 µL of **21a**). (II) mice were injected with *N*-ethylmaleimide (a canonical biothiols scavenger) for 30 min, then **21a** (50 µM, 100 µL) was injected and imaging performed after 20 min. (III) and (IV) mice were pre-injected with 1 mM *N*-ethylmaleimide and then injected intraperitoneally with (III) cysteine (1 mM, 50 µL) or (IV) homocysteine (1 mM, 50 µL), and then **21a** (50 µM, 100 µL) was injected and imaging occurred after 20 min. Excitation wavelength = 660 nm; Emission wavelength = 780 nm. (**C**,**D**) Representative in vivo photoacoustic images of mice subcutaneously injected with **21a**. Signals were collected under two laser light sources 695 [top panels of both (**C**,**D**)] and 840 nm [bottom panels of both (**C**,**D**)]. (VI) before injection with **21a** (50 µM, 100 µL); (VII) injection with **21a**; (VIII) injection with saline (100 µL, left leg) and cysteine (100 µL, 1 mM, right leg); (IX) before injection with **21a** (50 µM, 100 µL); (X) injection with **21a**; (XI) injection with saline (100 µL, left leg) and homocysteine (100 µL, 1 mM, right leg). Scale bar = 5 mm. Adapted by permission from Springer Nature and Copyright Clearance Center: Springer Nature, *Science China Chemistry*, Springer, Fang, H.; Chen, Y.; Wang, Y.; Geng, S.; Yao, S.; Song, D.; He, W.; Guo, Z. A dual-modal probe for NIR fluorogenic and ratiometric photoacoustic imaging of Cys/Hcy in vivo. *Sci. China Chem.*, **2020**, *63*, 699–706. Copyright 2020 [355].

Cysteine is a thiol that plays a vital role in redox homeostasis [74,75,76]. Levels of cysteine and homocysteine are closely related to multiple pathological processes; therefore, detection of these thiols is important for the early diagnosis of diseases including cancer [352,353,354]. Chen, He, and co-workers reported a near-IR fluorescence and photoacoustic dual-mode molecular probe, **21a**, that is activated by cysteine or homocysteine [355]. The probe undergoes nucleophilic substitution and Smiles rearrangement in response to the presence of cysteine or homocysteine. This reaction turns on near-IR fluorescence and ratiometric photoacoustic signals. In vitro and in vivo studies were performed using the molecular probe to investigate its applications in biomedical applications (Figure 44) [355].

**Figure 45:**
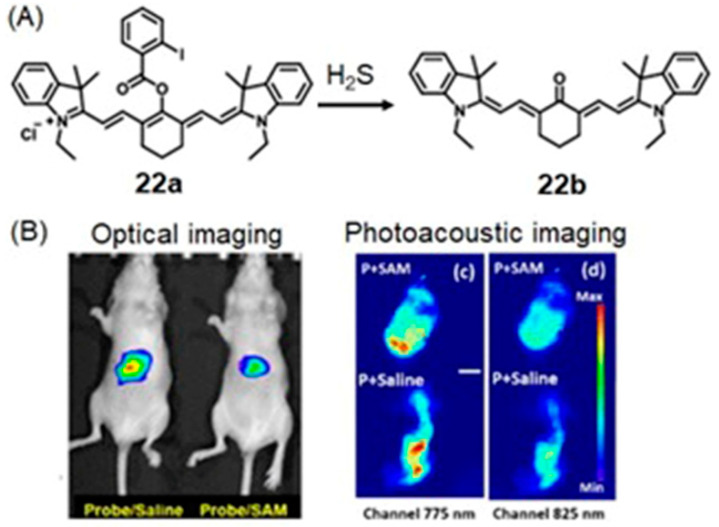
In vivo studies of complex **22** were performed using 6-week-old mice. (**A**) Chemical structures and schematic diagram of H_2_S-sensing mechanism of the probe. (**B**) Fluorescence images of mice injected with **22** and saline *S*-adenosyl-L-methionine (trigger H_2_S upregulation). Photoacoustic images of livers from mice injected with **22** and *S*-adenosyl-L-methionine and saline. (p indicates probe **22**). Images were recorded using channels 775 and 825 nm. Scale bar = 6 mm. Adapted with permission from Chen, Z.; Mu, X.; Han, Z.; Yang, S.; Zhang, C.; Guo, Z.; Bai, Y.; He, W. An optical/photoacoustic dual-modality probe: ratiometric in/ex vivo imaging for stimulated H_2_S upregulation in mice. *J. Am. Chem. Soc.*, **2019**, *141*, 17973–17977. Copyright 2019 American Chemical Society [356].

Homeostasis of H_2_S is disrupted in hypoxic tumors [99,100]. Yang, Guo, He, and co-workers reported a ratiometric optical/photoacoustic dual-mode probe that responds to H_2_S [356]. Some interferences inherent to both modalities including tissue scattering and autofluorescence are minimized in ratiometric imaging. The cyanine moiety of **22a** exhibits near-IR absorption and emission. In the presence of HS^−^, the nucleophilic substitution of the ester by HS^−^ produces enolic heptamethine cyanine that undergoes keto–enol tautomerization to form **22b** (Figure 45). Tautomerization shifts absorption and emission properties, enabling the detection of HS^−^ using a ratiometric method. In vivo assessment of the probe demonstrated that it detects upregulation of H_2_S in mouse livers using ratiometric optical and photoacoustic imaging (Figure 45) [356].

**Figure 46:**
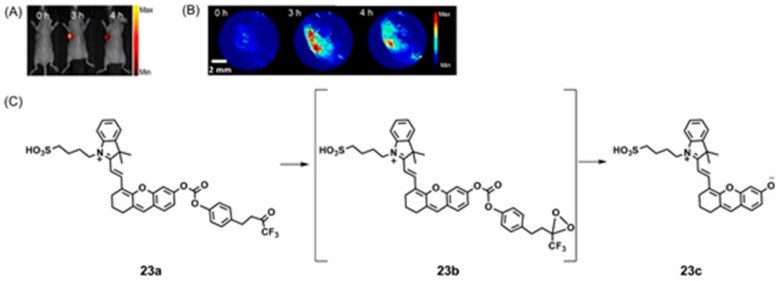
In vivo studies of the molecular probe **23**. (**A**) Real-time fluorescence images of tumor-bearing mouse after systemic administration of **23a** (50 µM in 100 µL saline) at 0, 3, and 4 h. (**B**) Representative photoacoustic maximum imaging projection of tumor from a systemic administration of a living mouse at 0, 3, and 4 h post-injection of **23a** (50 µM in 100 µL saline). (**C**) Chemical structures of **23a**, **23b**, and **23c**, and the conversion of **23a** to **23c**. Adapted with permission from Zhang, J.; Zhen, X.; Zeng, J.; Pu, K. A dual-modal molecular probe for near-infrared fluorescence and photoacoustic imaging of peroxynitrite. *Anal. Chem.*, **2018**, *90*, 9301–9307. Copyright 2018 American Chemical Society [357].

Zeng, Pu, and co-workers reported a hemicyanine dye that is caged by a trifluoromethyl ketone moiety for in vivo imaging of peroxynitrite (Figure 46) [357]. The probe is non-fluorescent when caged by the trifluoromethyl ketone moiety that contains an electron-donating oxygen atom. In the presence of ONOO^−^, the trifluoromethyl ketone undergoes a series of cascade oxidation–elimination reactions. As a consequence, a phenolic group is formed with enhanced electron-donating ability from the oxygen atom. The result is a fluorescence turn-on with red-shifted near-IR absorption, enabling the probe to be useful in dual-mode imaging. In vivo studies with 4T1 tumor-bearing mice demonstrated that both near-IR fluorescence and photoacoustic signals reached maximum values within three hours after tail vein administration of the probe. Relative to the background signals of the tumor, fluorescence, and photoacoustic signals exhibited 5.3- and 2.1-fold, respectively, greater intensities at three hours post-injection of the probe (Figure 46) [357].

Although levels of reactive oxygen species are important biomarkers of tumors, their reactivity, low abundance, and short-lived nature limit the ability to image these species in vivo [358]. However, the levels of reactive oxygen species are good indicators of hypoxic tumors, and therefore, it is important to develop probes that can accurately map these reactive species. Bohndiek and co-workers reported a probe (JW41) that contains a heptamethine carbocyanine dye scaffold, 2-deoxyglucose, and a boronic ester [359]. The dye exhibits fluorescence and photoacoustic properties, 2-deoxyglucose acts as tumor localization moiety, and the boronic ester reacts with hydrogen peroxide. In vivo studies demonstrated that after intravenous injection of the probe into tumor-bearing mice, it accumulated in tumors and visualized hydrogen peroxide in hypoxic cells [359].

**Figure 47:**
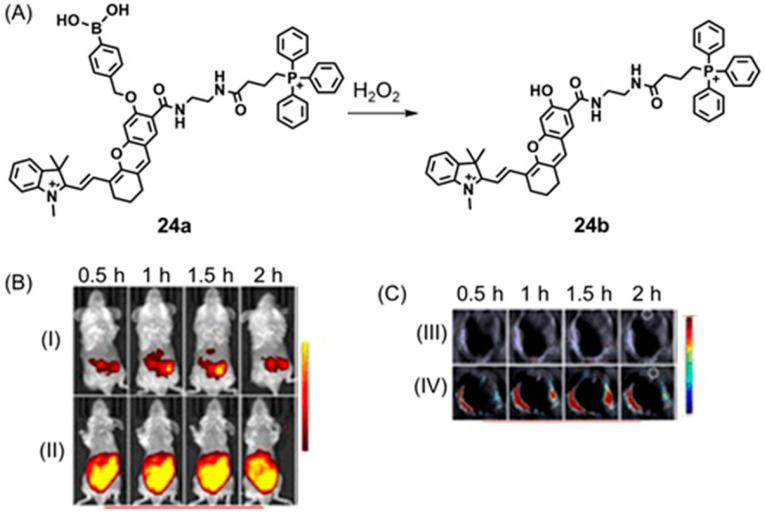
(**A**) Chemical structures of **24a** and **24b**. (**B**) Time-dependent near-IR fluorescence images and (**C**) time-dependent multispectral photoacoustic images of the abdomen of mice intraperitoneally injected with **24a** (250 µM, 200 µL). (**B**) (I) and (II) represent the (I) presence and (II) absence of lipopolysaccharide-induced inflammation. (**C**) (I) and (II) represent the (I) presence and (II) absence of lipopolysaccharide-induced inflammation. Adapted with permission from Chen, X.; Ren, X.; Zhang, L.; Liu, Z.; Hai, Z. Mitochondria-targeted fluorescent and photoacoustic imaging of hydrogen peroxide in inflammation. *Anal. Chem.*, **2020**, *92*, 14244–14250. Copyright 2020 American Chemical Society [360].

Hai and co-workers developed a near-IR probe for detection of hydrogen peroxide in mitochondria at a limit of detection of 0.348 μM [360]. The probe consists of three moieties that enable targeting of mitochondria, response to hydrogen peroxide, and generation of fluorescence and photoacoustic signals (Figure 47). Hemicyanine is a class of near-IR dyes that has outstanding absorption and emission properties due to intramolecular charge transfer mechanisms. Because the hydroxyl group of **24a** is caged before entering the mitochondria, fluorescence and photoacoustic signals are inhibited by intramolecular charge transfer transitions. Upon entering mitochondria, hydrogen peroxide reacts with the boronic acid moiety, causing its elimination from the probe. Removal of the boronic acid moiety exposes hemicyanine dye and subsequent 2.4- and 4.7-fold increases in fluorescence and photoacoustic signals, respectively, in HeLa cells (Figure 47) [360].

**Figure 48:**
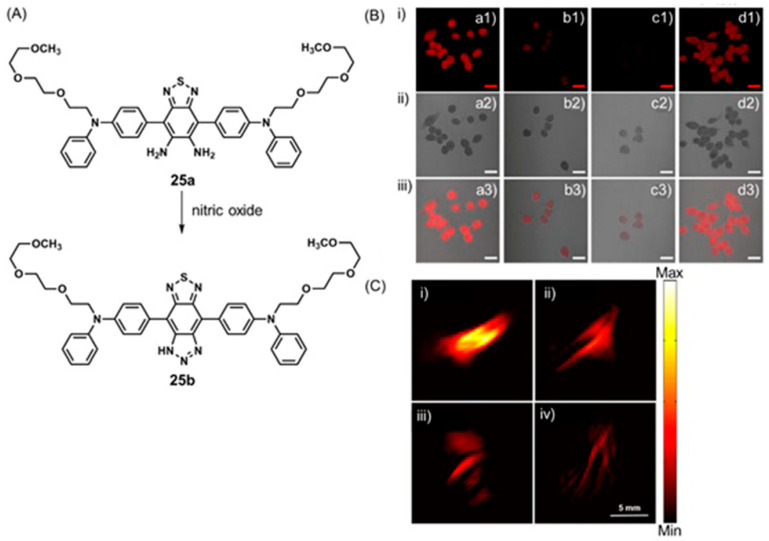
(**A**) Transformation of **25a** to **25b** in the presence of nitric oxide. (**B**) Confocal fluorescence images for detection of nitric oxide in RAW264.7 cells. (i), (ii), and (iii) represent the red channel, bright field, and merged images, respectively. Columns left to right represent (a) Cells incubated with the probe (2 µM) for 1 h. (b) and (c) The cells were pretreated with 2 and 5 µg mL^−1^ lipopolysaccharide, respectively, to induce inflammation for 4 h and then incubated with the probe (2 µM) for another 4 h. d) Cells were pretreated with *N*-acetylcystein (2 mM) to inhibit nitric oxide for 1 h, incubated with lipopolysaccharide (5 µg/mL) for 4 h, and then incubated with the probe (2 µM) for 4 h. Merged images were produced by combining the red channel and bright field. Excitation wavelength = 405 nm; emission wavelength = 500–600 nm. Scale bars = 20 µm. (**C**) In vivo photoacoustic images of subcutaneously injected seven-week-old mice (i) and (ii) induce inflammation by lipopolysaccharide (200 µL, 1 mg mL^−1^). (iii) and (iv) injected with phosphate-buffered saline (200 µL, pH 7.4) as reference sites. (i) and (iii) represent images acquired from the sites also subcutaneously injected with probe (150 µL, 10 µM). Adapted from *Sensors and Acutators B: Chemical*, *267*, Wang, S.; Li, Z.; Liu, Y.; Feng, G.; Zheng, J.; Yuan, Z.; Zhang, X. Activatable photoacoustic and fluorescent probe of nitric oxide for cellular and in vivo imaging, 403–411, Copyright 2018, with permission from Elsevier [361].

Nitric oxide plays an important role in hypoxia. Therefore, detection of nitric oxide in biological systems is important to the study of cancer. Zheng, Yuan, Zhang, and co-workers reported probe **25a** which contains diphenylamine and benzothiadiazolediamine to determine endogenous nitric oxide levels in cells using fluorescence and photoacoustic imaging (Figure 48) [361]. In the presence of nitric oxide under aerobic conditions, **25a** forms complex **24b**. This change results in an increase in intramolecular charge transfer in the structure. The probe has an absorption centered at 368 nm, and upon reacting with nitric oxide, the absorption peak shifts to 568 nm. This difference can be used to detect nitric oxide using photoacoustic imaging. Additionally, the probe has a fluorescence on–off response and excellent selectivity for nitric oxide compared to other reactive-oxygen and reactive-nitrogen species [361].

**Figure 49:**
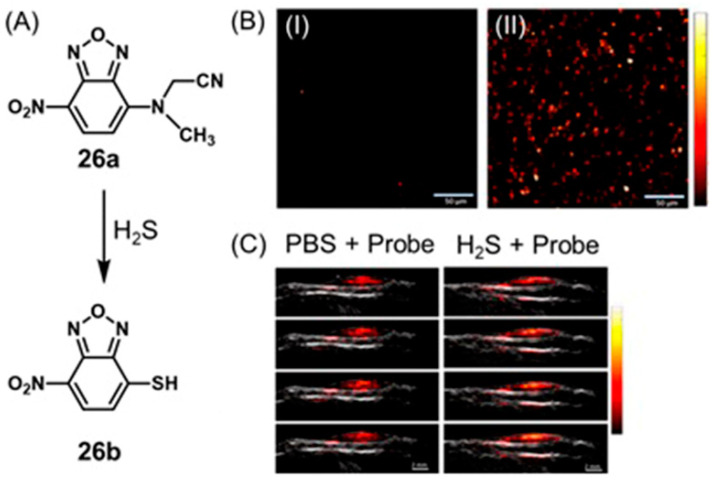
(**A**) Chemical structures of **26a** and **26b**. (**B**) Photoacoustic images of HeLa cells incubated with (I) **26a** and (II) pretreated with **26a** (50 µM) for 30 min and then incubated with H_2_S (500 µM) for 40 min. Excitation wavelength = 532 nm; pulse energy = 100 nJ; scale bars = 50 µm. (**C**) Time-dependent photoacoustic images of living mice subcutaneously injected into a leg of each mouse. Excitation wavelength = 532 nm; fluence = 1.5 mJ/cm^2^. Matrigel mixed with phosphate-buffered saline (150 µL) and **26a**. Matrigel mixed with H_2_S in phosphate-buffered saline (150 µL, final concentration 30 mM) and **26a** (50 µL, 100 mM). Adapted with permission from Zhang, J.; Wen, G.; Wang, W.; Cheng, K.; Guo, Q.; Tian, S.; Liu, C.; Hu, H.; Zhang, Y.; Zhang, H.; Wang, L.; Sun, H. Controllable cleavage of C–N bond-based fluorescent and photoacoustic dual-modal probes for the detection of H_2_S in living mice. *ACS Appl. Bio Mater.*, **2020**, *4*, 2020–2025. Copyright 2020 American Chemical Society [365].

Hypoxia can be studied by determining the amount of hydrogen sulfide in diseased tissue [99,100]. For example, hydrogen sulfide is significantly increased in colon cancer and thus imaging hydrogen sulfide in vivo enables the accurate diagnosis of colon cancer [362,363,364]. Wang, Sun, and co-workers reported nitrobenzoxadiazole amine derivatives that undergo H_2_S-specific thiolysis by cleavage of a C–N bond (Figure 49) [365]. Cleavage of the C–N bond was accelerated by incorporating electron-withdrawing groups. Compound **26a** showed a large fluorescence on–off response rate (*k*_2_ = 4.04 M^−1^ s^−1^) and selectivity for H_2_S over other thiols. Cleavage of the C–N bond of **26a** by H_2_S and formation of **26b** results in a bathochromic shift and decrease in fluorescence signal. On the other hand, due to the strong absorption of light by **26b**, the photoacoustic signal is enhanced. Therefore, the probe exhibits fluorescence quenching and photoacoustic signal enhancement in response to the presence of H_2_S. Studies with HeLa cells treated with **26a** demonstrated a decrease in fluorescence signals in the presence of H_2_S (200 μM) and a strong photoacoustic signal that is about 2.9-fold larger than that of the control group consisting of only **26a** without H_2_S [365].

**Figure 50:**
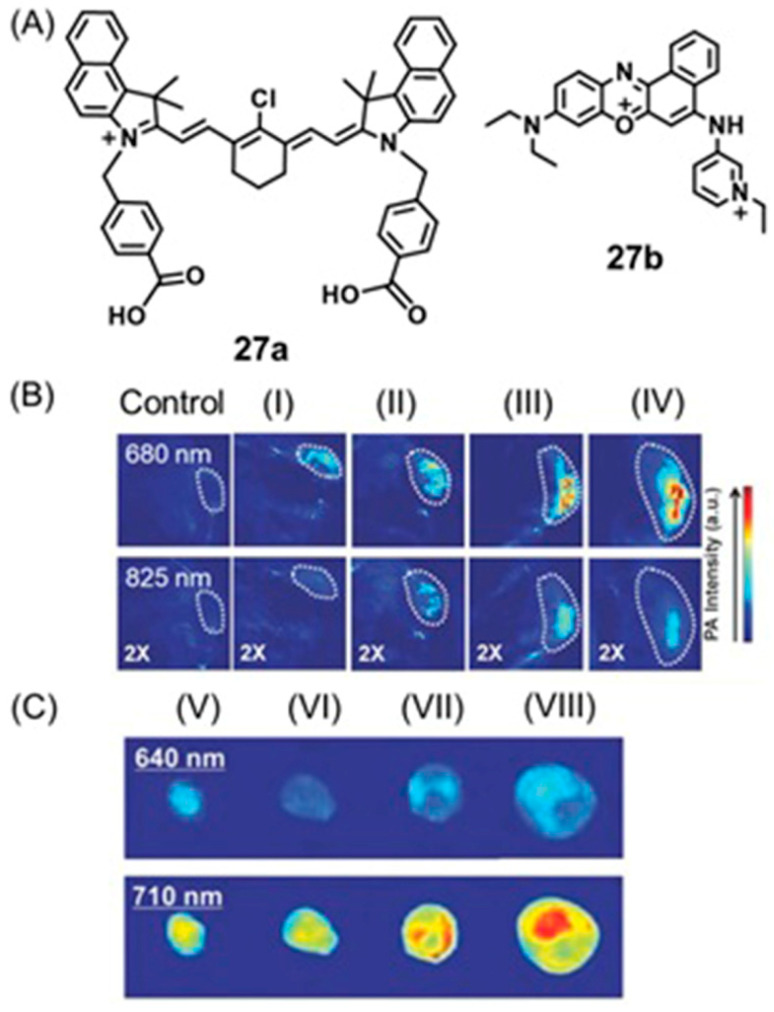
(**A**) Chemical structures of **27a** and **27b**. (**B**) Photoacoustic images of tumors having different sizes after mice were intravenously injected with the **27a**. Images were recorded at 680 and 825 nm excitations (II) 6, (III) 10, (IV) 14, and 18 days after injection. (I) represents the control (tumor without **27a**). (**C**) Ex vivo fluorescence images of tumor with different sizes collected from mice (V) 6, (VI) 10, (VII) 14, and (VIII) 18 days after intravenous injection of **27a**. Adapted with permission from Chen, Q.; Liu, X.; Chen, J.; Zeng, J.; Cheng, Z.; Liu, Z. *Advanced Materials*, John Wiley and Sons [361].

Zeng, Liu, and co-workers reported a self-assembled albumin-based nanoprobe for in vivo pH mapping using photoacoustic and fluorescence imaging [366]. Two near-IR dyes, **27a** and **27b**, were induced to self-assemble with human serum albumin to form albumin–dye nanocomplexes. Absorbance and fluorescence of **27b** are inert to pH changes, and thus **24b** serves as an internal reference. Dye **27a** is pH-responsive and acts as a pH indicator under both ratiometric photoacoustic and fluorescence imaging. In vivo applications of the nanoprobe as a pH indicator were demonstrated using tumor-bearing mice (Figure 50) [366].

**Figure 51:**
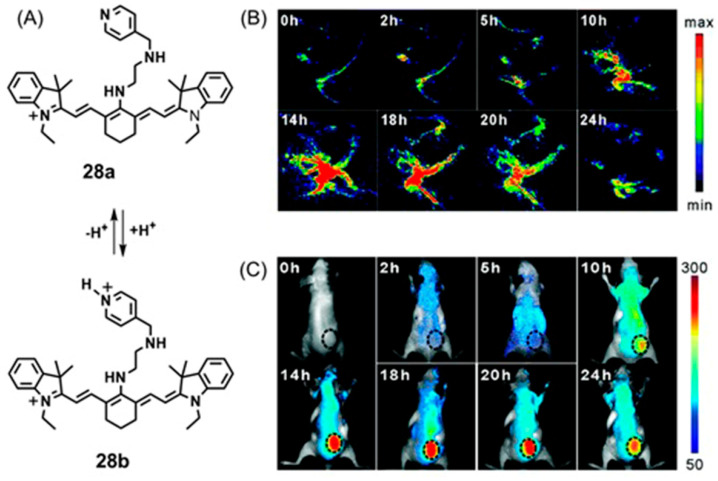
(**A**) Chemical structures of **28a** and **28b**. (**B**) Photoacoustic images of the tumor sites of MCF-7 tumor-bearing mice at different time points after intravenous injection of **28a**. (**C**) near-IR fluorescence images of the tumor sites (represented by black dashed circles) of MCF-7 tumor-bearing living nude mice at different time points after intravenous injection of **28a**. Adapted with permission of the Royal Society of Chemistry, from Tumor-targeted small molecule for dual-modal imaging-guided phototherapy upon near-infrared excitation. Meng, X.; Li, W.; Sun, Z.; Zhang, J.; Zhou, L.; Deng, G.; Gong, P.; Cai, L. 5, 47, **2017**; permission conveyed through Copyright Clearance Center, Inc. [367].

Gong, Cai, and co-workers reported a pH-sensitive small molecule for near-IR fluorescence and photoacoustic imaging [367]. The near-IR dye consisted of heptamethine cyanine which possesses high extinction coefficients and tumor accumulation. The dye was combined with a proton receptor that specifically accumulates in tumors, and the fluorescence of **27a** is increased in acidic tumor microenvironments. The proton receptor exhibits photoacoustic imaging, and thus the probe can be used for pH imaging in hypoxic tumors. In vivo near-IR fluorescence and photoacoustic imaging studies of tumor-bearing mice demonstrated that the probe exhibits localization in the tumor and pH-responsiveness (Figure 51). Albumin-dye nanocomplexes reported by Zeng, Liu, and co-workers and the heptamethine cyanine near-IR dye-containing compound reported by Gong, Cai, and co-workers both require relatively long times for specific tumor imaging (10 h to days) [366,367]. Therefore, to enhance the tumor-targeting ability, it is important to incorporate tumor-targeting molecules into the imaging agents.

**Figure 52:**
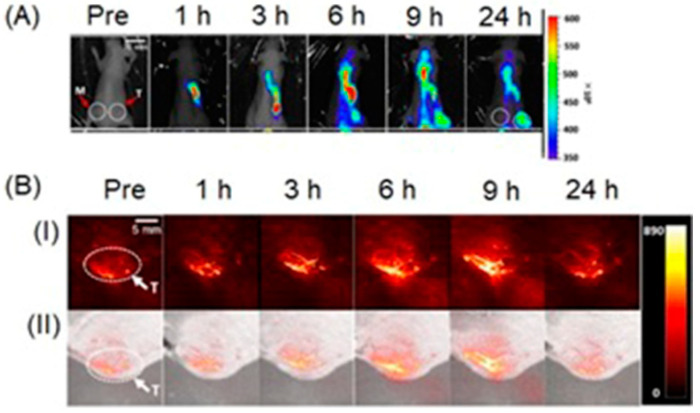
(**A**) Real-time fluorescence images of tumor-bearing mouse after administration of dye (100 µM in 200 µL of saline) at different time points. White dashed circles indicate the tumor site. M = muscle. T = tumor. (**B**) Photoacoustic images of A549 tumor-bearing mice were obtained before and 1, 3, 6, 9, and 24 h after injection of dye (100 µM in 200 µL of saline). (I) and (II) represent photoacoustic and merged images, respectively. White dashed circles indicate the tumor sites. T = tumor. Adapted with permission from Mu, H.; Miki, K.; Harada, H.; Tanaka, K.; Nogita, K.; Ohe, K. pH-activatable cyanine dyes for selective tumor imaging using near-infrared fluorescence and photoacoustic modalities. *ACS Sens.*, **2021**, *6*, 123–129. Copyright 2021 American Chemical Society [368].

Miki, Ohe, and co-workers reported pH-activatable cyanine dyes for selective tumor imaging using near-IR fluorescence and photoacoustic modalities [368]. The dye contains a cyclic structure under basic conditions, blocking the π-conjugation of indocyanine green for the near-IR absorption, making the molecule non-emissive. Under acidic conditions found in hypoxic tumors, the ring-opening process consequently recovers both fluorescence and photoacoustic signals (Figure 52). Tumor-targeting was achieved by incorporating tumor-targeting short peptide (cRGD), and short imaging times (6 h post-injection) were observed relative to previously reported methods (Figure 52) [368].

**Figure 53:**
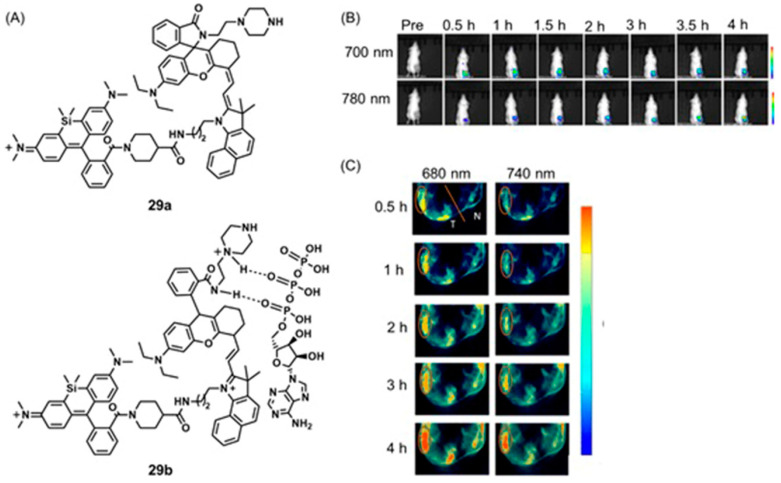
(**A**) Chemical structures of **29a** and **29b**. (**B**) Time-dependent dual-channel fluorescence images of tumor-bearing mice after intravenous injection of **29a**. Excitation wavelength = 660 nm; emission wavelengths = 700 ± 10 and 780 ± 10 nm. (**C**) Time-dependent dual-channel photoacoustic images of tumor-bearing mice after intravenous injection of **29a**. Excitation wavelengths = 680 and 740 nm. Adapted with permission from Liu, X.; Gong, X.; Yuan, J.; Fan, X.; Zhang, X.; Ren, T.; Yang, S.; Yang, R.; Yuan, L.; Zhang, X.-B. Dual-stimulus responsive near-infrared reversible ratiometric fluorescent and photoacoustic probe for in vivo tumor imaging. *Anal. Chem.*, **2021**, *93*, 5420–5429. Copyright 2021 American Chemical Society [369].

Most responsive probes are activated by a single stimulation. As a consequence, false results can be generated in terms of tumor-specific imaging. Therefore, it is important to develop probes that are responsive to more than one stimulus to enhance the accuracy of studies [40]. Yang, Yuan, and co-workers reported a dual-stimulus responsive near-IR reversible adenosine 5′-triphosphate (ATP) and pH probe, **29a**, for fluorescence and photoacoustic ratiometric imaging of tumors [369]. In hypoxic tumor microenvironments, H^+^ and ATP content are greater than in normal tissues because of the high tumor metabolic rate. Probe **29a** was constructed using silicon rhodamine as the donor, and a dye as the acceptor. Upon binding both H^+^ and ATP into the acceptor units, the probe is activated. The probe was used in ratiometric photoacoustic and fluorescence tumor imaging to demonstrate its suitability for in vivo applications (Figure 53) [369].

### 5.6. Photoacoustic Imaging and MRI

Combining MRI and photoacoustic imaging enables detection of hypoxia-related pathological conditions with less error than either modality individually. Photoacoustic imaging is a highly sensitive method that overcomes the low sensitivity of MRI. MRI overcomes the low soft tissue contrast and limited depth penetration of photoacoustic imaging.

**Figure 54:**
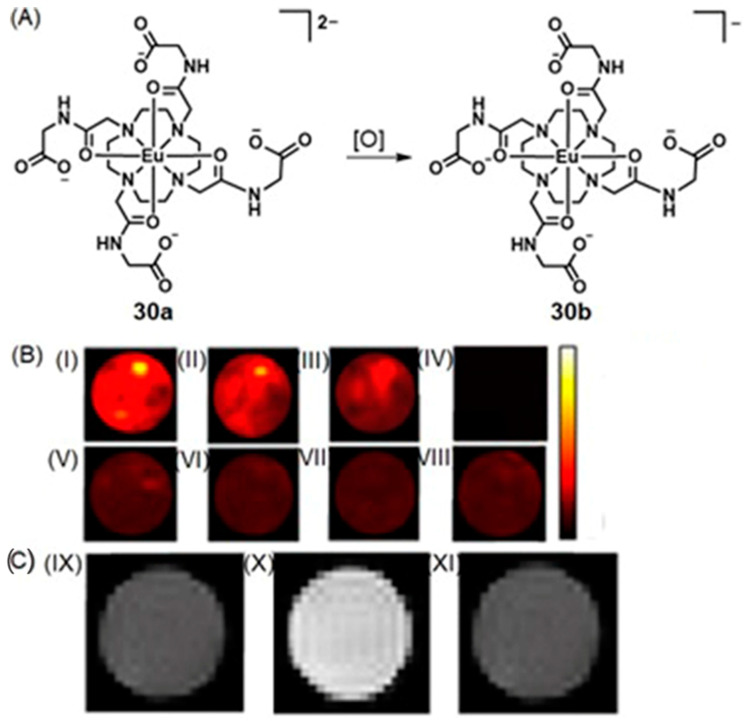
(**A**) Oxidation of **30a** to **30b** in the presence of oxygen. (**B**) Photoacoustic images of (I) **30a** (100 mM in phosphate-buffered saline, pH 7.4); (II) **30a** (60 mM in phosphate-buffered saline, pH 7.4); (III) **30a** (30 mM in phosphate-buffered saline, pH 7.4); (IV) optical glass tube (wall thickness = 0.35 mm) containing distilled water; (V) **30b** (100 mM in phosphate-buffered saline, pH 7.4); (VI) **30b** (60 mM in phosphate-buffered saline, pH 7.4); (VII) **30b** (30 mM in phosphate-buffered saline, pH 7.4); and (VIII) phosphate-buffered saline (pH 7.4). The color map represents the normalized intensity for photoacoustic images. (**C**) *T*_1_-weighted MRI of (IX) phosphate-buffered saline (pH 7.4); (X) **30a** (30 mM in phosphate-buffered saline, pH 7.4); and (XI) **30b** (30 mM in phosphate-buffered saline, pH 7.4). The tube diameters for MRI and photoacoustic imaging were 3 and 5 mm, respectively. Adapted with permission [370].

Redox potential is tightly regulated in cells, but hypoxia can trigger an imbalance of the levels of redox-active molecules in cells [99,100]. Mehrmohammadi, Allen, and co-workers reported oxidation-responsive Eu-containing species **30a** and **30b** that are detected using MRI and photoacoustic imaging (Figure 54) [370]. Complex **30a** shows positive contrast enhancement in *T*_1_-weighted MRI and bright photoacoustic images relative to complex **30b**. Previous experiments performed using complexes **30a** and **30b** demonstrated that **30a** is oxidized to **30b** when exposed to oxygen. Therefore, **30a** is stable under hypoxic conditions, and both MRI and photoacoustic signals turn off upon oxidation into **30b** [370].

**Figure 55:**
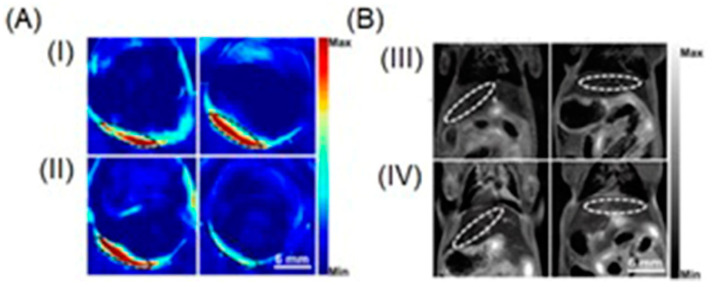
(**A**) Photoacoustic images of mice (I) before and (II) after intraperitoneally injected with Prussian blue and then treated with (left) saline and (right) paracetamol. (**B**) *T*_2_-weighted MRI images of mice (III) before and (IV) after intraperitoneally injected with Prussian blue and then treated with (left) saline and (right) paracetamol. Adapted with permission from Chen, F.; Teng, L.; Lu, C.; Zhang, C.; Rong, Q.; Zhao, Y.; Yang, Y.; Wang, Y.; Song, G.; Zhang, X. Activatable magnetic/photoacoustic nanoplatform for redox-unlocked deep-tissue molecular imaging in vivo via Prussian blue nanoprobe. *Anal. Chem.*, **2020**, *92*, 13452–13461. Copyright 2020 American Chemical Society [371].

Song and co-workers reported an activatable nanoprobe based on Prussian blue that combines MRI and photoacoustic imaging for deep-tissue ONOO^−^ imaging [371]. ONOO^−^ oxidizes Fe^II^ into Fe^III^. Oxidation results in the typical blue color of Prussian blue nanoparticles changing to light-yellow. As a consequence, the photoacoustic signal arising from the absorption at 710 nm due to electron transfer between Fe^II^ and Fe^III^ is weakened. Moreover, ONOO^−^ ions degrade Prussian blue nanoparticles into small fragments that shorten rotational correlation times and reduce the brightness of *T*_1_- and *T*_2_-weighted MRI. Therefore, both MRI and photoacoustic signal reduction were used as an indicator for sensing ONOO^−^ ions in female mice that had elevated levels of ONOO^−^ ions (Figure 55) [371].

**Figure 56:**
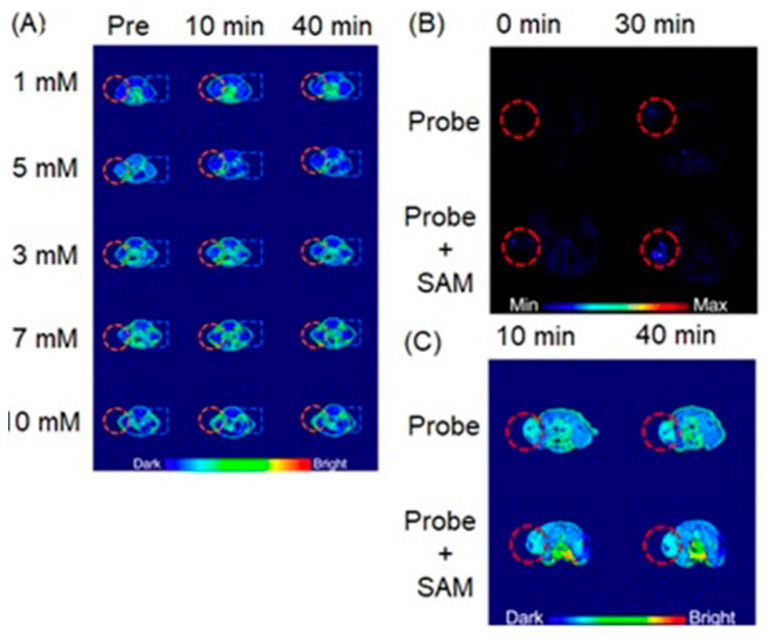
(**A**) *T*_1_-weighted MRI images of nude mice administered with probe and different amounts of NaHS (1, 3, 5, 7, and 10 mM; left side of the mouse) or phosphate-buffered saline (control; right side of the mouse) acquired before and at 10 and 40 min after injection. Orange dashed circles indicate the H_2_S-enhanced regions. Control regions are indicated by blue dashed rectangles. (**B**) In vivo photoacoustic images of HCT-116 tumor-bearing mice acquired at 0 and 30 min after administration of the nanoprobe. (**C**) In vivo *T*_1_-weighted images of HCT-116 tumor-bearing mice acquired at 0 and 30 min after administration of the nanoprobe. *S*-adenosyl methionine (SAM) was used to facilitate the enhancement of photoacoustic signal. Adapted with permission from Yan, C.; Liu, D.; An, L.; Wang, Y.; Tian, Q.; Lin, J.; Yang, S. Magnetic–photoacoustic dual-mode probe for the visualization of H_2_S in colorectal cancer. *Anal. Chem.*, **2020**, *92*, 8254–8261. Copyright 2020 American Chemical Society [372].

Among overexpressed thiols in hypoxic cells, H_2_S signals oxygen deficiency in tissues. An, Tian, Yang, and co-workers constructed core–shell Fe_3_O_4_@Cu_2_O-lipid-mPEG (polyethylene glycol) nanoparticles that detect endogenous H_2_S in colon tumors [367]. H_2_S in the cellular environment can partially vulcanize Cu_2_O in the shell to form porous copper sulfide and, consequently, enhance near-IR absorption resulting in photoacoustic signals. Moreover, due to the formation of the holes, inner-sphere water exchange at the Fe_3_O_4_ core is accelerated, generating contrast enhancement in *T*_1_-weighted MR images. In vivo studies performed using tumor-bearing mice with elevated levels of H_2_S exhibited bright photoacoustic and *T*_1_-weighted MRI in tumor sites compared to healthy tissues in the same mouse (Figure 56) [372].

**Figure 57:**
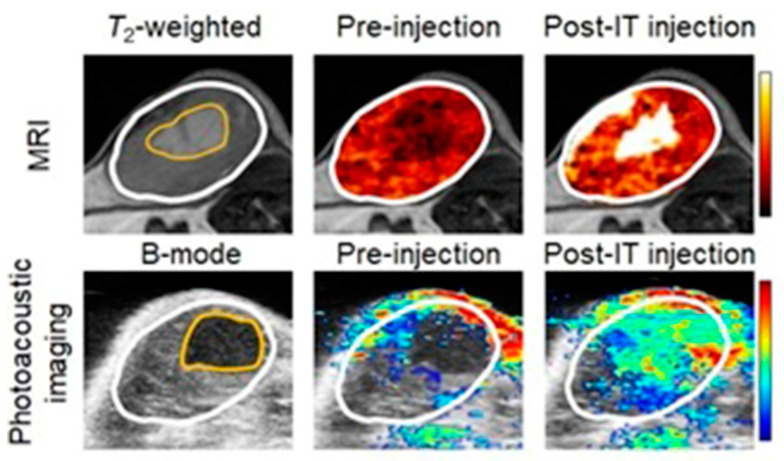
Ultrasound-guided intratumoral (IT) injection of MnO_2_-containing nanoparticles into head and neck squamous cell carcinoma-bearing mice. Panels of *T*_2_-weighted MRI and photoacoustic images showing before and after direct delivery of nanoparticles to hypoxic region (outlined in yellow) within the tumor (outlined in white). Adapted with permission [373].

Prasad and co-workers reported ultra-small NaYF_4_:Nd^III^/NaGdF_4_ nanocrystals coated with manganese dioxide (MnO_2_) for modulation and imaging of hypoxia in head and neck squamous cell carcinomas (Figure 57) [373]. MnO_2_ is oxidized to Mn^II^ in acidic environments by hydrogen peroxide to produce O_2_ and H_2_O. Degradation of MnO_2_ not only releases paramagnetic Mn^II^ but also exposes Gd^III^ incorporated in the ultrasmall nanoparticles that enhance contrast in *T*_1_-weighted MRI. Because dissociation of MnO_2_ produces O_2_, photoacoustic imaging was used to differentiate the optical absorption properties between oxygenated hemoglobin and deoxygenated hemoglobin to estimate oxygen saturation in tumor hypoxic environments [373].

**Figure 58:**
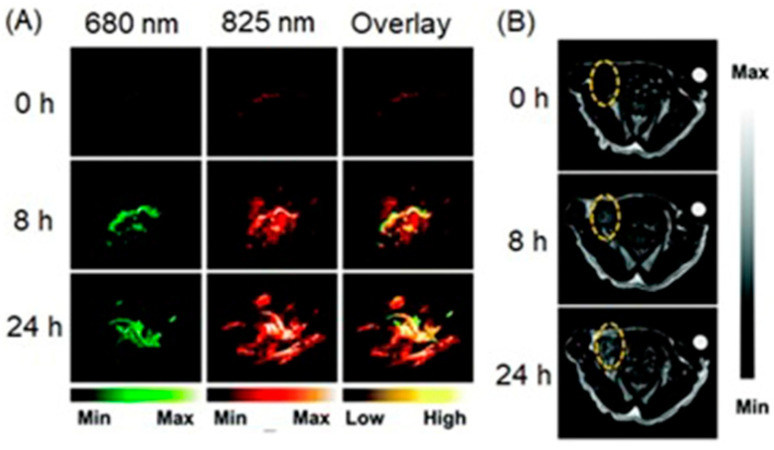
(**A**) Representative photoacoustic images and (**B**) representative *T*_1_-weighted images of 4T1 neoplastic mice after tail vein injection of nanoparticles. The two photoacoustic imaging channels represent hydrogen peroxide/pH-sensitive MnO_2_ nanoparticles that exhibit photoacoustic signal at 680 nm and hydrogen peroxide/pH-inert polymeric nanoparticles that exhibit photoacoustic signal at 825 nm. Adapted with permission of the Royal Society of Chemistry, from Intelligent polymer–MnO_2_ nanoparticles for dual-activatable photoacoustic and magnetic resonance bimodal imaging in living mice. Hu, X.; Zhan, C.; Tang, Y.; Lu, F.; Li, Y.; Pei, F.; Lu, X.; Ji, Y.; Li, J.; Wang, W.; Fan, Q.; Huang, W. *55*, 43, **2019**; permission conveyed through Copyright Clearance Center, Inc. [374].

Lu, Fan, and co-workers examined polymer–MnO_2_ nanoparticles for dual-activatable photoacoustic and magnetic resonance bimodal imaging in tumor-bearing mice [369]. MnO_2_ nanoparticles in their native form do not appreciably enhance the contrast of *T*_1_-weighted images. However, MnO_2_ nanoparticles exhibit near-IR absorption and serve as photoacoustic contrast agents for intravital imaging. Upon entry of the nanoparticles into hypoxic tumors, *T*_1_-weighted MRI signals are enhanced because of the degradation of MnO_2_ in the presence of hydrogen peroxide and mildly acidic conditions. Because the photoacoustic signal of MnO_2_ nanoparticles turns off in tumor microenvironments, the particles were conjugated with a fluorophore to show near-IR absorption with a peak at 825 nm (Figure 58) [374].

**Figure 59:**
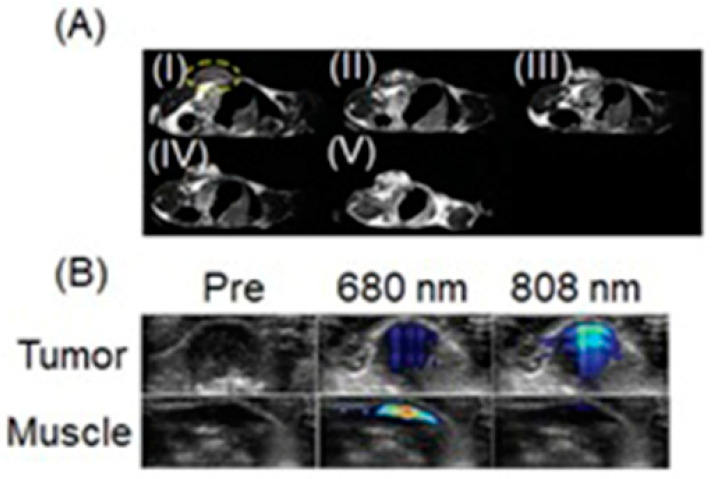
(**A**) Coronal MRI images of an EMT-6 tumor-bearing mouse before and after multi-site injection of the complex (16 mM Fe). The tumor site is indicated by yellow dashed circle in each image. Injected volumes are (I) 0, (II) 25, (III) 50, (IV) 75, and (V) 100 µL. (**B**) Photoacoustic imaging of a mouse with an EMT-6 tumor (upper panels) or muscle (bottom panels) injected with the Fe^III^-containing complex and under pulse laser irradiation at different wavelengths. Adapted with permission from Liang, G.; Han, J.; Xing, D. *Advanced Healthcare Materials*, John Wiley and Sons [375].

Liang, Han, and Xing reported a pH-responsive Fe^III^-containing complex that can be used to monitor tumor photothermal therapy by magnetic resonance imaging and photoacoustic imaging [375]. The complex exhibits both *T*_1_-weighted MRI contrast-enhancing properties and optical absorption in the red and near-IR regions of the electromagnetic spectrum. Optical absorption of the complex is pH-sensitive, with greater absorption intensity in weakly acidic environments relative to neutral environments. With this system, MRI contrast enhancement and photoacoustic signals were used to monitor photothermal therapy of tumors (Figure 59) [375].

### 5.7. Complementary MRI Methods

MRI offers different complementary imaging modes that enable some limitations of any specific method to be overcome. In this section, different modes of MRI are described that are combined to image hypoxia and hypoxia-related pathological conditions.

#### 5.7.1. T_1_- and T_2_-Weighted MRI

**Figure 60:**
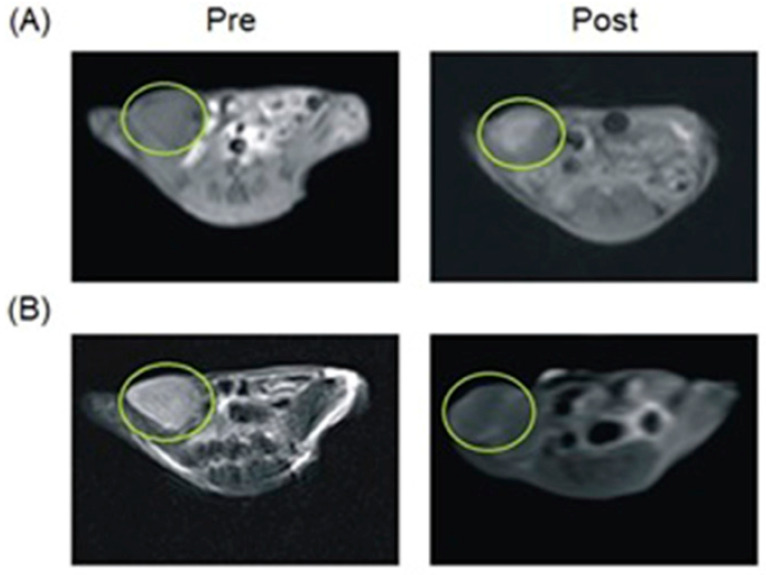
(**A**) *T*_1_-weighted and (**B**) *T*_2_-weighted MRI at 3.0 T of tumor-bearing mice before and after injection of nanoparticles. Yellow circles mark the tumor site. Adapted with permission [377].

Among molecular imaging techniques, MRI is excellent in soft tissue contrast. Additionally, the sensitivity of the method can be increased using contrast agents to enhance contrast in regions of interest. However, when using a single contrast agent to image hypoxic tissues, there are challenges to overcome to achieve accurate results. One approach to overcome this limitation is to combine *T*_1_- and *T*_2_-weighted contrast agents creating *T*_1_/*T*_2_ dual-mode probes for MRI to produce accurate information about conditions relevant to hypoxia [376]. Wang, Han, Chen, and co-workers reported a hypoxia-targeted *T*_1_- and *T*_2_-based dual-mode contrast agent for MRI [377]. The contrast agent was synthesized using polyethylene glycol and Mn^II^-modified iron oxide nanoparticles that are assembled with oligonucleotide HIF-1α aptamers. An HIF-1α-based aptamer ensures the accumulation of the nanoparticles in the hypoxic region. In vitro and in vivo studies were performed using human pancreatic carcinoma cell lines and a xenograft of Panc-1 showed enhanced accumulation of the nanoparticles in hypoxic regions (Figure 60) [377].

**Figure 61:**
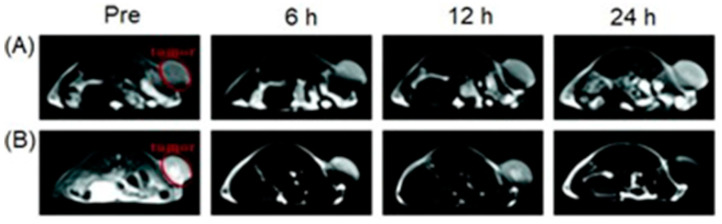
(**A**) *T*_1_- and (**B**) *T*_2_-weighted MRI images of a 4T1 tumor-bearing mouse. MRI images were acquired before and 6, 12, and 24 h after injection of the nanocomposites (10 mg kg^−1^). Solutions of nanocomposites were injected into the mouse through tail vein at different times. Red circles highlight the tumor site. Adapted with permission of the Royal Society of Chemistry, from Albumin/sulfonamide stabilized iron porphyrin metal organic framework nanocomposites: targeting tumor hypoxia by carbonic anhydrase IX inhibition and *T*_1_–*T*_2_ dual mode MRI guided photodynamic/photothermal therapy. Zhu, W.; Liu, Y.; Yang, Z.; Zhang, L.; Xiao, L.; Liu, P.; Wang, J.; Yi, C.; Xu, Z.; Ren, J. *6*, 2, **2018**; permission conveyed through Copyright Clearance Center, Inc. [378].

The carbonic anhydrase IX enzyme is overexpressed in the hypoxic microenvironment in the tumor [69]. Xu, Ren, and co-workers reported albumin/sulfonamide-stabilized iron porphyrin metal-organic framework nanocomposites that target tumor hypoxia through accumulation in tumors overexpressing carbonic anhydrase IX [378]. Iron in the reported nanocomposite serves as a contrast agent for *T*_1_- and *T*_2_-weighted MRI (*r*_1_ = 2.7 mM^−1^ s^−1^ and *r*_2_ = 19.68 mM^−1^ s^−1^). In vivo MRI ability of the nanocomposite was evaluated using a 4T1 tumor-bearing mouse. The results showed that the tumor location exhibited enhanced images relative to the pre-injection image (Figure 61). These results demonstrate that the nanocomposite-based *T*_1_/*T*_2_ contrast enhancement generated in MRI can be used to image hypoxic regions in tumor sites [378].

**Figure 62:**
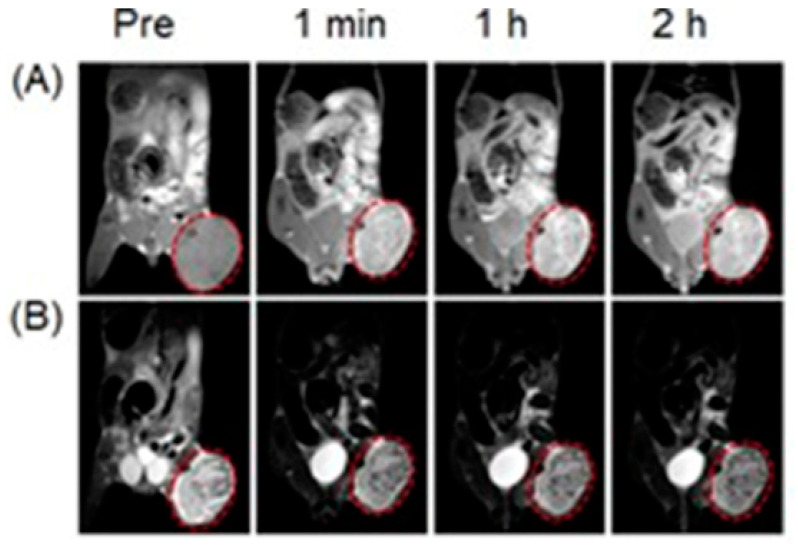
(**A**) *T*_1_- and (**B**) *T*_2_-weighted MRI of tumor-bearing mice obtained before (Pre) and after (1 min, 1 h, and 2 h) intravenous injection of nanocrystals. Red dotted circles represent the tumor sites. Adapted from *Biomaterials*, *101*, Kim, M.-H.; Son, H.-Y.; Kim, G.-Y.; Park, K.; Huh, Y.-M.; Haam, S. Redoxable heteronanocrystals functioning magnetic relaxation switch for activatable *T*_1_ and *T*_2_ dual-mode magnetic resonance imaging. 121–130, Copyright 2016, with permission from Elsevier [379].

Glutathione plays a central role in cell growth and function, and hypoxic tumor cells have greater glutathione concentrations than corresponding normal cells [100]. Haam, Huh, and co-workers reported redox-responsive heteronanocrystals, consisting of a superparamagnetic Fe_3_O_4_ core, a paramagnetic Mn_3_O_4_ shell, and a surface modified with polysorbate 80 [379]. The Mn_3_O_4_ shell is a redox-active switch that is activated by glutathione in intracellular reducing environments. Mn_3_O_4_ also protects the Fe_3_O_4_ core from aqueous environments to attenuate *T*_2_ relaxation. Upon reduction in Mn_3_O_4_, magnetically decoupled Mn^II^ ions (for *T*_1_ relaxation enhancement) are released, enabling Fe_3_O_4_ to interact with the water protons (*T*_2_ relaxation enhancement). The ability to use the reported material was demonstrated via effective passive tumor targeting for *T*_1_- and *T*_2_-weighted MRI in tumor-bearing mice (Figure 62) [379].

**Figure 63:**
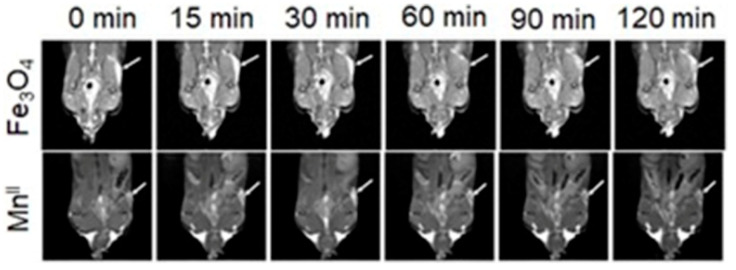
*T*_1_- and *T*_2_-weighted MRI of tumor-bearing mice acquired pre- and post-injection of Mn^II^ and Fe_3_O_4_ samples. The tumor is marked by white arrows. Adapted from *Biomaterials*, *194*, Sun, X.; Zhang, G.; Du, R.; Xu, R.; Zhu, D.; Qian, J.; Bai, G.; Yang, C.; Zhang, Z.; Zhang, X.; Zou, D.; Wu, Z. A biodegradable MnSiO_3_@ Fe_3_O_4_ nanoplatform for dual-mode magnetic resonance imaging guided combinatorial cancer therapy, 151–160, Copyright 2019, with permission from Elsevier [380].

Zou, Wu, and co-workers reported MnSiO_3_- and Fe_3_O_4_-containing nanoplatforms that are responsive to weakly acidic and large-concentration-glutathione conditions found in tumor microenvironments [380]. Fe_3_O_4_ nanoparticles effectively cover the pores of MnSiO_3_, and upon entering the weakly acidic and concentrated glutathione levels in the tumor microenvironment, the structure of the nanoplatform decomposes releasing Mn^II^. Both Fe_3_O_4_ and Mn^II^ contribute to *T*_2_ and *T*_1_ relaxation enhancement. In vivo studies demonstrated that the nanoplatform targets the weakly acidic and large glutathione levels that are characteristic of hypoxic tumor microenvironments (Figure 63) [380].

**Figure 64:**
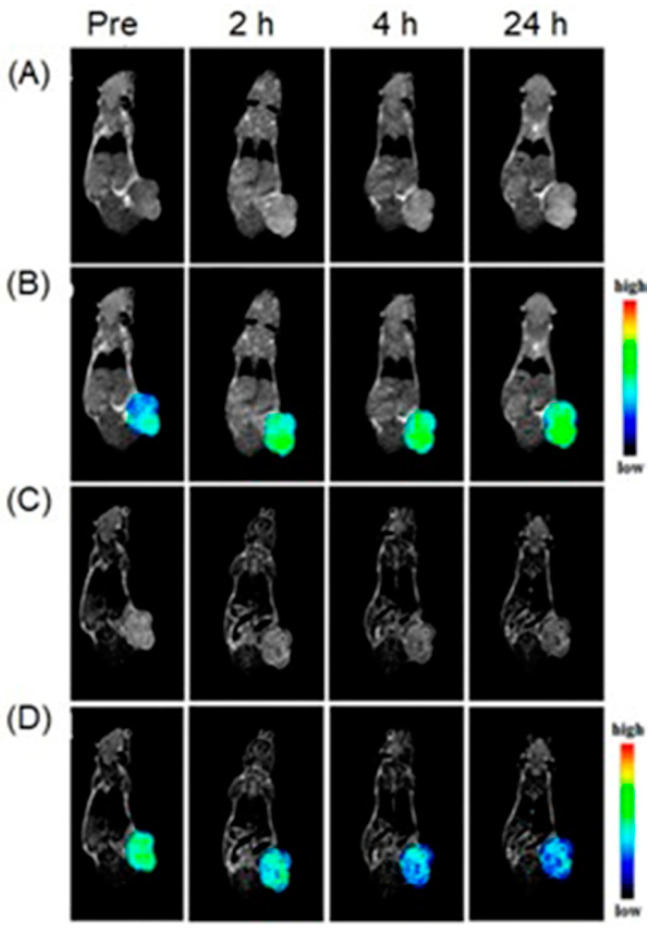
(**A**,**B**) *T*_1_-weighted and (**C**,**D**) *T*_2_-weighted MRI images of a tumor-bearing mouse before and after intravenous injection of nanocomposites at different times. Adapted with permission from Jin, L.; Liu, J.; Tang, Y.; Cao, L.; Zhang, T.; Yuan, Q.; Wang, Y.; Zhang, H. MnO_2_-Functionalized Co–P nanocomposite: a new theranostic agent for pH-triggered *T*_1_/*T*_2_ dual-modality magnetic resonance imaging-guided chemo-photothermal synergistic therapy. *ACS Appl. Mater. Interfaces*, **2017**, *9*, 41648–41658. Copyright 2017 American Chemical Society [381].

Yuan and co-workers reported a pH-responsive MnO_2_ functionalized cobalt phosphide nanocomposite to image hypoxic tumors [381]. MnO_2_ in the nanocomposite degrades upon entering the weakly acidic tumor microenvironment and releases Mn^II^ ions and activates *T*_1_-weighted contrast enhancement in MRI. A cobalt phosphide core enabled *T*_2_-weighted MRI contrast enhancement. Results show that the signal intensity of *T*_1_-weighted images of the tumor increased after injection, while the *T*_2_-weighted images became darker (Figure 64). The observed signal enhancements were attributed to the cellular uptake and tumor accumulation of the nanocomposite, owing to the enhanced permeability and retention effect and tumor microenvironment responsiveness after administration [381].

#### 5.7.2. ^1^H- and ^19^F-MRI

A challenge of contrast-enhanced MRI is the concentration dependency of contrast enhancement that is difficult to determine in vivo; therefore, strategies are needed to overcome the concentration dependency of contrast agents [40]. This section includes descriptions of multimodal contrast agents that elicit orthogonal responses with two imaging modalities and thereby enhance the accuracy of hypoxia detection and overcoming the concentration dependency of contrast agents for MRI.

**Figure 65:**
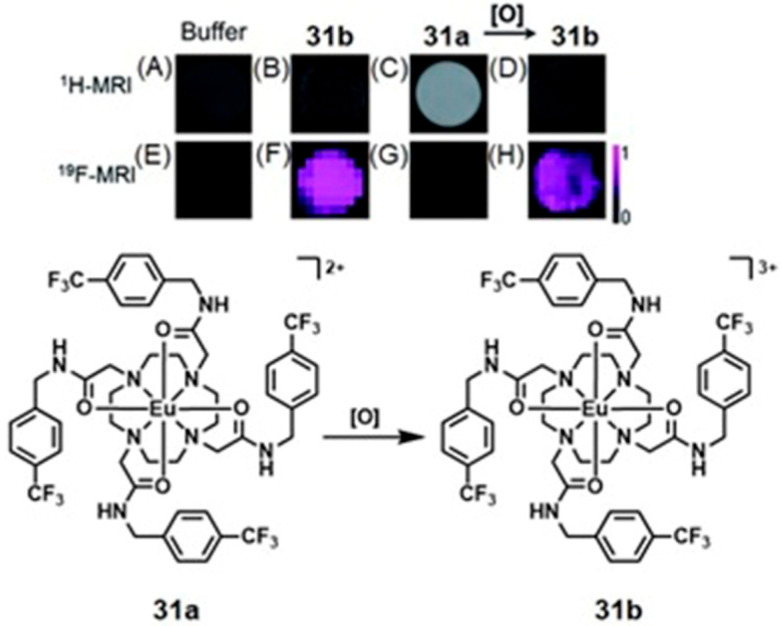
*T*_1_-weighted MRI images of solutions of (**A**) aqueous 3-morpholinipropane-1-sulfonic acid buffer (20 mM, pH 7.0); (**B**) **31b** (3.5 mM); (**C**) **31a** (3.5 mM); (**D**) **31b** after oxidation of **31a** (3.5 mM). ^19^F-MRI images of (**E**) aqueous 3-morpholinipropane-1-sulfonic acid buffer (20 mM, pH 7.0); (**F**) **31b** (3.5 mM); (**G**) **31a** (3.5 mM); (**H**) **31b** after oxidation of **31a** (3.5 mM). The scale bar on the right of (H) represents signal intensity for all ^19^F-MRI images in the figure. Chemical structures of **31a** (left) and **31b** (right). Adapted with permission of the Royal Society of Chemistry, from Fluorinated Eu^II^-based multimodal contrast agent for temperature- and redox-responsive magnetic resonance imaging, Basal, L. A.; Bailey, M. D.; Romero, J.; Ali, M. M.; Kurenbekova, L.; Yustein, J. T.; Pautler, R. G.; Allen, M. J. *8*, 12, **2017**; permission conveyed through Copyright Clearance Center, Inc. [393].

Some redox couples including Eu^II/III^, Cu^I/II^, Fe^II/III^, Co^II/III^, and Mn^II/III^ exhibit redox responsiveness in biologically relevant environments [382,383,384,385,386,387,388,389,390,391,392]. The different oxidation states of these metal ions exhibit different responses in MRI. For example, one oxidation state of a contrast agent might produce ^1^H-MRI signal enhancement, and upon oxidation, the resultant ion completely turns off ^1^H-MRI signal enhancement and produces a ^19^F-MRI signal. Pautler, Allen, and co-workers reported a fluorinated Eu^II^-containing multimodal contrast agent, **31a** (Figure 65) that exhibits orthogonal responses towards ^1^H- and ^19^F-MRI [393]. Bright *T*_1_-weighted images were obtained after injecting **31a** into the oxygen-rich intraperitoneal cavity of a mouse. After 9 min post-injection, the *T*_1_-weighted signal intensity disappeared and the ^19^F signal appeared (Figure 65). The concentration of oxygen in cells is a metric of hypoxia; therefore, the development of oxygen-responsive probes is important in studying hypoxia and hypoxia-related pathological conditions. In this work, the advantage of ^1^H- and ^19^F-MRI to orthogonally detect the contrast agent in vivo has been used to study the amount of oxygen present in the intraperitoneal cavity of the mouse [393].

**Figure 66:**
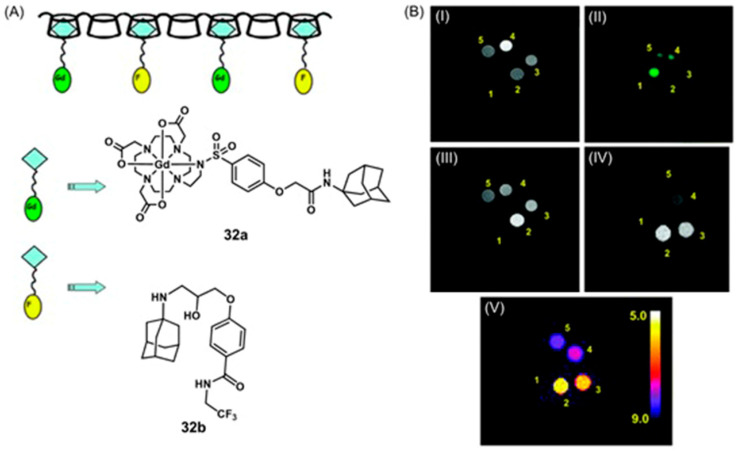
(**A**) Schematic representation of the supramolecular adduct between poly-β-cyclodextrin, Gd^III^-containing complex, **32a**, and the ^19^F-reporting molecule **32b** designed for pH mapping. (**B**) MRI images of solutions of (1) NaPF_6_ (25 mM); (2) poly-cyclodextrin (10 mM); **32b** (2.5 mM); **32a** (0.5 mM) at pH = 6.8; (3) poly-cyclodextrin (13 mM); **32b** (3.25 mM); **32a** (0.65 mM) at pH = 7.2; (4) poly-cyclodextrin (30 mM); **32b** (7.5 mM); **32a** (1.5 mM) at pH = 7.6; (5) poly-cyclodextrin (20 mM): **32b** (5 mM); **32a** (1 mM) at pH = 8.2. (I) *T*_1_-weighted spin-echo ^1^H-MR image acquired at 7.1 T (TR = 80, TE = 4.4, NEX = 32, 1 slice, 1 mm); (II) *T*_1_-weighted spin-echo ^19^F-MRI acquired at 7.1 T (TR = 2000, TE = 1.4, NEX = 384, 1 slice, 3 mm); (III) *T*_1_-weighted spin-echo ^1^H-MR image acquired at 7.1 T and normalized to the concentrations found from image (II); (IV) *T*_1_-weighted spin-echo ^1^H-MR image acquired at 1 T (TR = 80, TE = 7.2, NEX = 32), 1 slice, 2 mm) and normalized to the concentrations found from image (II); (V) pH-map image derived from image (IV). Adapted with permission of the Royal Society of Chemistry, from Poly-β-cyclodextrin based platform for pH mapping via a ratiometric ^19^F/^1^H MRI method. Gianolio, E.; Napolitano, R.; Fedeli, F.; Arena, F.; Aime, S. *40*, **2009**; permission conveyed through Copyright Clearance Center, Inc. [394].

Aime and co-workers used a combination of ^1^H- and ^19^F-MRI for measuring pH by incorporating **33a** and **33b** into poly-β-cyclodextrin [394]. The pH responsiveness is provided by **33a** with a coordination cage consisting of a tetraazamacrocycle bearing three acetate arms and one substituted sulfonamide arm. The protonation–deprotonation step of the sulfonamide moiety takes place at pH = 6.7. Upon deprotonation, the sulfonamide enters the coordination sphere of Gd^III^ by replacing the two inner-sphere water molecules. The pH-dependent change in hydration of Gd^III^ results in a change in the observed relaxivity of the complex. Furthermore, by controlling the molar ratio of **33a**/**33b**/poly-β-cyclodextrin (1:5:20), the ^19^F-MRI signal was measured and quantified using an external standard (25 mM of NaPF_6_). A proof of concept of this approach was demonstrated in vitro by acquiring ^1^H- and ^19^F-MRI of four samples containing the polymeric material at different concentrations and pH values (Figure 66) [394].

**Figure 67:**
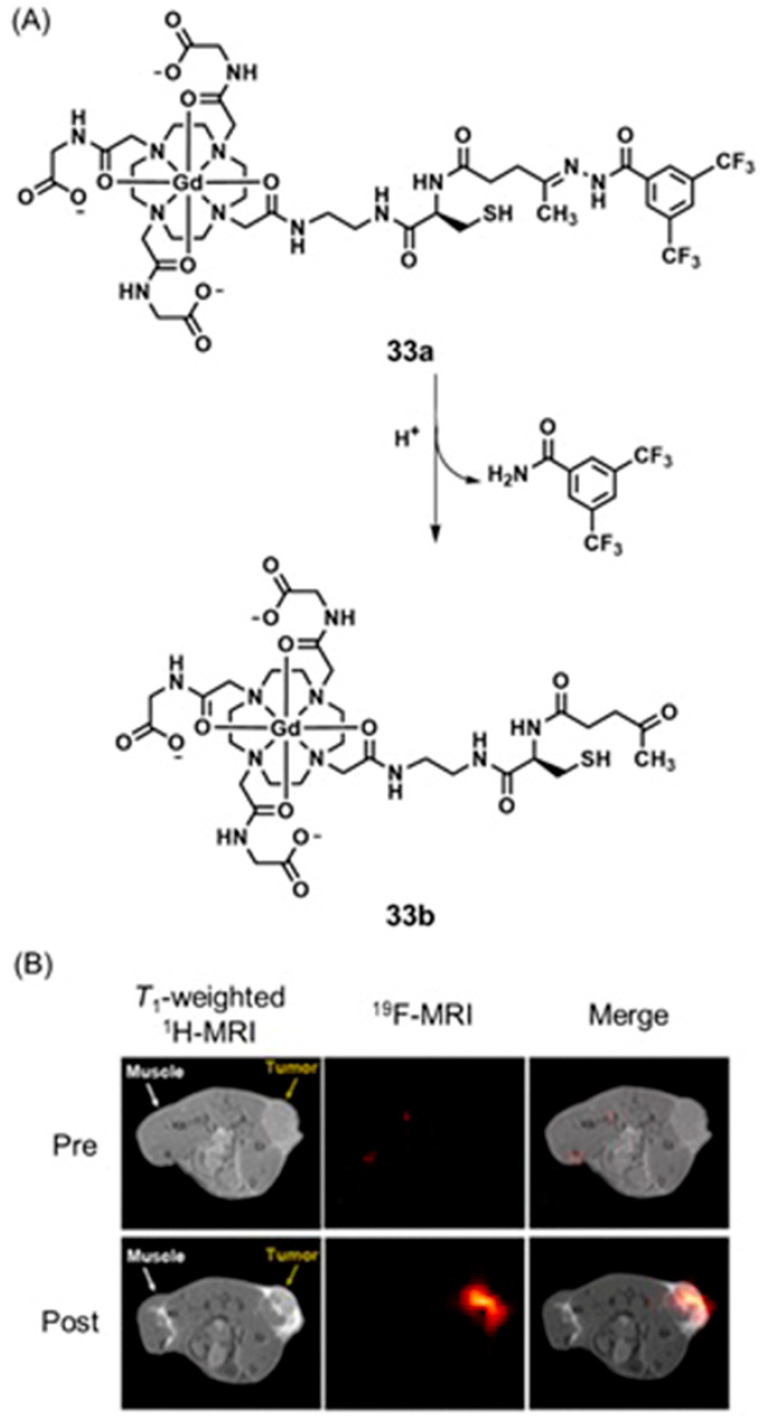
(**A**) Chemical reaction behind the ^19^F signal recovery of **33a** in response to mildly acidic tumor microenvironment. (**B**) *T*_1_-weighted ^1^H-MRI and ^19^F-MRI of tumor-bearing mice before and after administration of fluorinated gadolinium chelate-grafted palladium nanoconjugates at 9.4 T. Yellow and white arrows indicate the tumor and muscle, respectively. Adapted with permission from Tang, X.; Gong, X.; Ming, J.; Chen, D.; Lin, H.; Gao, J. Fluorinated Gadolinium Chelate-Grafted Nanoconjugates for Contrast-Enhanced *T*_1_-Weighted ^1^H and pH-Activatable ^19^F Dual-Modal MRI. *Anal. Chem.*, **2020**, *92*, 16293–16300. Copyright 2020 American Chemical Society [395].

Lin, Gao, and co-workers reported fluorinated gadolinium chelate-grafted palladium nanoconjugates for contrast-enhanced *T*_1_-weighted ^1^H-MRI and pH-activatable ^19^F-MRI [395]. Thw **33a** in the nanoconjugate acts as a contrast agent for ^1^H-MRI. The paramagnetic relaxation enhancement effect exerted by Gd^III^ on ^19^F causes abatement of the ^19^F signal. Upon entering mildly acidic tumor microenvironments, cleavage of the hydrazone bond separates ^19^F nuclei from Gd^III^ in **33b** and subsequently increases the signal in ^19^F-MRI. In vivo studies demonstrated that this activatable probe can be used for contrast-enhanced *T*_1_-weighted and pH-responsive ^19^F dual-modal MRI for the sensitive and accurate study of the hypoxic regions in tumor-bearing mice (Figure 67) [395].

#### 5.7.3. *T*_1_-Weighted MRI and CEST

**Figure 68:**
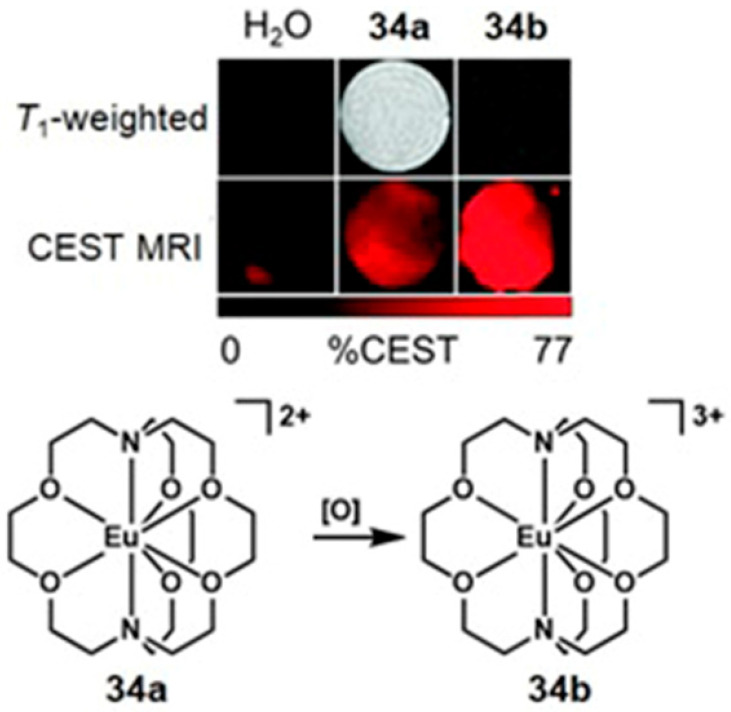
(Left to right) MR images of water, Eu^II^-containing liposomes, Eu^III^-containing liposomes (5 mm tube diameter, at 7 T and 24 °C). *T*_1_-weighted images (**top row**) and CEST map generated by subtracting presaturation at 1.2 ppm from presaturation at −1.2 ppm and the difference was divided by presaturation at −1.2 ppm (**bottom row**). %CEST represents the decrease in bulk water signal intensity as a result of presaturation of exchangeable water protons associated with liposomes. Adapted with permission of the Royal Society of Chemistry from Oxidation-responsive Eu^2+/3+^-liposomal contrast agent for dual-mode magnetic resonance imaging, Ekanger, L. A.; Ali, M. M.; Allen, M. J. *50*, 94, **2014**; permission conveyed through Copyright Clearance Center, Inc. [396].

Dual-mode hypoxia-responsive probes have been designed based on contrast agents that are responsive to complementary MRI techniques, including *T*_1_-MRI and CEST. Use of probes that exhibit on/off responses to complementary MRI techniques not only enables unambiguous detection of hypoxia but also can be used to confirm the physiological status of microenvironments. Allen and co-workers used the ability of Eu^II^ to oxidize to Eu^III^ under aerobic physiologically relevant conditions, to design an oxidation-responsive dual-mode contrast agent [396]. Eu^II^ and Eu^III^ ions exhibit orthogonal responses towards *T*_1_-weighted MRI and CEST. Liposome encapsulated Eu^II^-containing complex, **34a**, produces bright *T*_1_-weighted MR images but no observable signal in CEST. Upon oxidation under ambient atmosphere, the *T*_1_ relaxation time of the liposome solution containing **34a** decreased by up to 86%. As a consequence, *T*_1_-weighted images of liposome solution were indistinguishable compared to that of water. However, oxidation resulted in the recovery of the CEST signal (Figure 68) [396].

**Figure 69:**
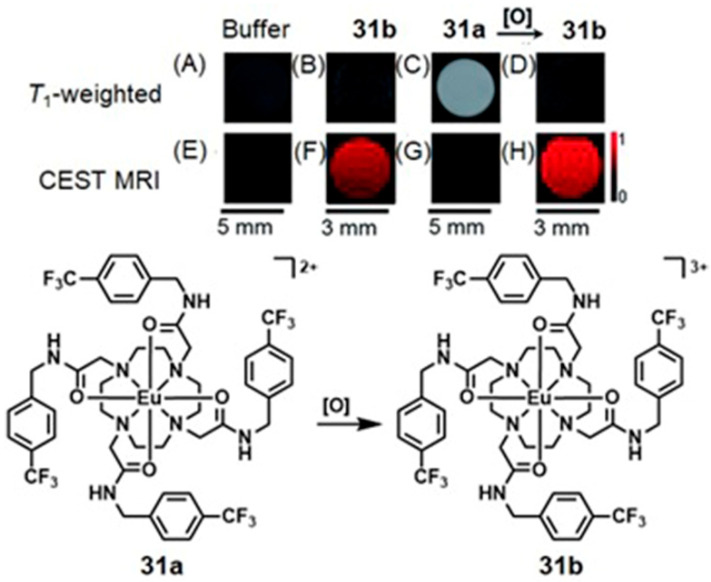
MRI of solutions of samples in aqueous 3-morpholinopropane-1-sulfonic acid buffer (20 mM, pH 7.0) in glass (3 mm) or plastic tubes (5 mm). Scale bars correspond to the three images in the column above each bar. (Top row) *T*_1_-weighted MRI images of solutions of (**A**) aqueous 3-morpholinopropane-1-sulfonic acid buffer (20 mM, pH 7.0); (**B**) **31b** (3.5 mM); (**C**) **31a** (3.5 mM); and (**D**) **31b** after oxidation of **31a** (3.5 mM). (Bottom row) CEST MRI images of (**E**) aqueous 3-morpholinopropane-1-sulfonic acid buffer (20 mM, pH 7.0); (**F**) **31b** (3.5 mM); (**G**) **31a** (3.5 mM); and (**H**) **31b** after oxidation of **31a** (3.5 mM). The scale bar on the right of (h) represents signal intensity for all CEST MRI images in Figure 69. Adapted with permission of the Royal Society of Chemistry, from Fluorinated Eu^II^-based multimodal contrast agent for temperature- and redox-responsive magnetic resonance imaging, Basal, L. A.; Bailey, M. D.; Romero, J.; Ali, M. M.; Kurenbekova, L.; Yustein, J. T.; Pautler, R. G.; Allen, M. J. *8*, 12, **2017**; permission conveyed through Copyright Clearance Center, Inc. [398].

An in vivo study with a Eu^III^-containing tetraamide complex was reported by Ratnakar, Kovacs, and co-workers [397]. After injecting the complex into mice, the complex generated strong *T*_1_ enhancement. The brightness of the *T*_1_-weighted image gradually diminished over several minutes followed by the appearance of a strong CEST effect at the injection site. This effect was caused by the oxidation of Eu^II^ into Eu^III^. Additionally, a fluorinated Eu^II^-containing complex, **31a**, reported by Pautler, Allen, and co-workers exhibits redox sensitivity towards an oxidative environment [393]. Under reduced oxygen levels, **31a** showed strong *T*_1_ enhancement leading to a bright *T*_1_-weighted image. Upon oxidization into **31b** under aerobic conditions, a dark *T*_1_-weighted image and bright CEST image were obtained (Figure 69). These pre-clinical studies demonstrate that Eu^II^- and Eu^III^-containing complexes that exhibit *T*_1_-weighted and CEST imaging capabilities are potential contrast agents that can be used to detect oxygen levels and redox species in hypoxic tumors [393].

**Figure 70:**
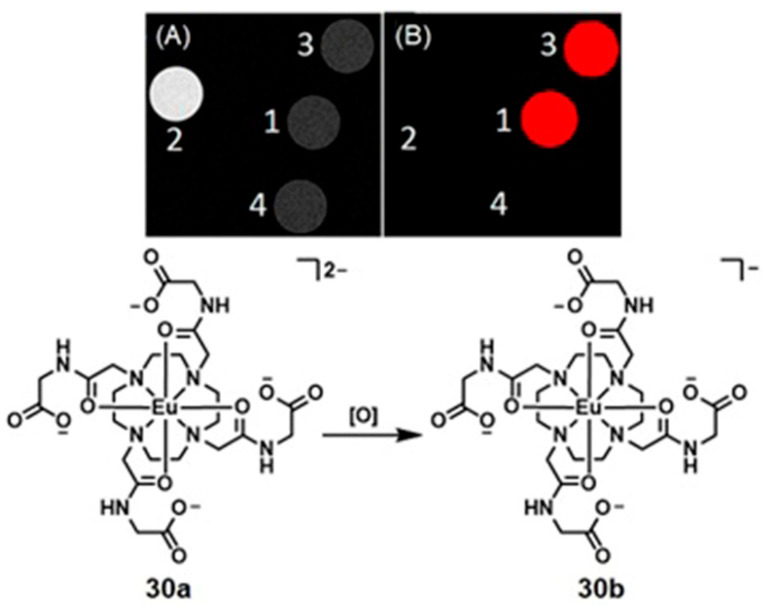
Images of (**A**) *T*_1_-weighted MRI and (**B**) CEST difference (55 ppm image subtracted from the –55 ppm image) of 5 mm diameter tubes containing (1) **30b**, (2) **30a**, (3) **30b** after oxidation of **30a**, and (4) water. Adapted with permission from Ekanger, L. A.; Mills, D. R.; Ali, M. M.; Polin, L. A.; Shen, Y.; Haacke, E. M.; Allen, M. J. Spectroscopic characterization of the 3+ and 2+ oxidation states of europium in a macrocyclic tetraglycinate complex. *Inorg. Chem.*, **2016**, *55*, 9981–9988. Copyright 2016 American Chemical Society [398].

Allen and co-workers reported a Eu^II^- and Eu^III^-containing tetraglycenate complexes as *T*_1_-weighted MRI and CEST agents [398]. The reduced version of the complex exhibits a strong *T*_1_ shortening effect and consequently produces a bright *T*_1_-weighted image, and it quenches the CEST signal. Upon oxidation into Eu^III^ under aerobic conditions, the Eu^III^-containing complex does not appreciably shorten the *T*_1_ relaxation time of nearby water protons but recovers the CEST signal and thus produces a bright CEST image (Figure 70) [398].

## 6. Conclusions

Dual-mode imaging is a promising method to provide comprehensive information for hypoxia research. The construction of dual-mode probes with sensitive responses to microenvironments not only combines complementary advantages of two imaging modalities, but also minimizes the limitations of single-mode probes that otherwise are amplified in complex physiological environments. For example, dual-mode imaging is used to overcome the limitation of concentration-dependent probe response [40]. It also can provide more information about tumor microenvironments than single imaging modalities [280,282,298]. When a complementary modality is selected, dual-mode imaging overcomes the limitations of single-mode techniques, including limited spatial resolution, low depth penetration, and low sensitivity [123,125].

In this review, advances in the sensitive detection of cancer using dual-mode imaging systems and a diverse group of responsive probes for hypoxia, oxidative stress, pH, and dysregulation of redox homeostasis are described. However, despite recent progress, most attempts are still in the preclinical stage and are not used in clinical imaging due to several challenges. Therefore, innovative strategies are needed to fill the gap between benchtop research and clinical imaging. Some aspects that are likely to be useful in future research to bridge laboratory research and tumor hypoxia imaging in cancer patients include the following:

(1) Dual-mode radiometric probes to overcome limitations of individual imaging modalities, for example, concentration dependency of contrast agents in MRI. Dual-mode imaging enables the integration of complementary advantages while minimizing the limitations of each method; however, these advantages and limitations are unique to instrumentation or the physics of methodology (for example, energy, resolution, sensitivity, and background signal). The use of ratiometric probes is important to uniquely address some of the critical challenges associated with each imaging modality (concentration dependency and potential interferences associated with heterogeneous physiological environments) and ratiometric probes enable obtaining precise information about environments and minimizes the chances of observing false negative and false positive results.
(2) Dual-mode probes to detect relevant differences in physiological environments. Almost all reported probes are unable to detect the early stages of hypoxia. For example, pH-sensitive probes undergo stimuli-responsive changes in the probe when the pH is usually about 6.0–6.5. If a probe could detect a pH value slightly less than 7.4, such tissues could be flagged as potential cancer early, although with a secondary confirmation to avoid false positives. In PET imaging, most tracers detect tumors when the oxygen concentration is less than the clinically relevant hypoxic level (<10 mmHg). Detection of early stages of hypoxia can help maximize the survival rate of cancer patients; therefore, the development of dual-mode probes that are responsive to small changes in physiological conditions is highly desirable in future biomedical applications.
(3) Increased biocompatibility of dual-mode probes for clinical imaging. Functionalizing dual-mode probes to increase biocompatibility, target solubility (water or lipid), and target specificity while minimizing toxicity is needed to use dual-mode probes in clinical imaging. Furthermore, imaging agents should be developed to remain active long enough that the instrument can detect the probe within an imaging-relevant time frame but short enough to clear within a reasonable amount of time.
(4) Development of multiple stimuli-responsive probes. Reported dual-mode probes to date are responsive to specific changes associated with tumor microenvironments. However, the development of probes that are capable of simultaneously detecting more than one stimulus is likely to provide a more precise and accurate characterization of hypoxic tumors.
(5) Integration of dual-mode diagnostic agents with therapeutic agents to detect and treat cancer simultaneously. New physical and chemical theories have been used in synthesizing activity-based sensing probes for tumor imaging and therapy [399,400]. This strategy has the potential to minimize patient exposure to chemicals and harmful radiation and increase the efficiency of clinical diagnosis and therapy of cancer, under the right circumstances. Furthermore, theragnostic dual-mode probes could minimize potential side effects that arise due to the use of multiple probes.
(6) The use of multiple imaging modalities to obtain accurate information about tumor microenvironments. Several multi-modal imaging probes have been reported to measure hypoxia. The inclusion of extra modalities, when selected for a specific purpose, can further overcome the limitations of using only two modalities; however, care must be shown to reasonably determine the utility of these extra modalities.

In summary, hypoxia is a critical medical condition that needs to be diagnosed in its early stages to increase the survival chances of patients and to develop new treatments for diseases. Dual-mode imaging of hypoxia has the potential to enable accurate diagnosis while overcoming the limitations of single-mode imaging techniques. Existing methods are still not capable of capturing subtle changes in tissue environment that help to identify tumors in their early stages. As a result, there is an opportunity to incorporate new ideas into existing methods and use them in clinical imaging. As opposed to its advantages, dual-modality imaging of hypoxia has limitations as well. For example, the necessity to use more than one imaging mode can be costly and time-consuming, and the design and synthesis of multimodal probes can be challenging. We expect that this review article will provide guidance for future development of imaging science focused on understanding and imaging hypoxia using more than one imaging modality at a time.

## Figures and Tables

**Table 1:** Summary of hypoxia-mediated pathophysiological changes in tumor microenvironment.

Change	Chemicals Involved	Causes	Consequences
pH	Lactic acid, H^+^, CO_2_	Partial degradation of glucose, imbalance in ion exchange between intra- and extra-cellular environment.	Tumor acidosis.
Reactive species	^•^OH, O_2_^•−^, H_2_O_2_, NOX, NO^•^, ONOO^−^, H_2_S	Partial cellular respiration.	Promote tumor growth.
Redox buffers	NADH, Glutathione, NADPH	Production of reactive oxygen species.	Promote tumor growth.
Inorganic ions	Ca^2+^, PO_4_^3−^, ATP	Incomplete cellular respiration, production of reactive oxygen species.	Help survival of tumor cells.

**Table 2:** Summary of imaging modalities and their properties frequently used for tumor imaging.

Modality	Type of Information	Spatial Resolution (mm)	Penetration Depth	Probe/Agent Sensitivity	References
Optical	anatomical or tracer	0.1–10	<1 cm	10^−9^–10^−12^ M	[119,120,121,122,123]
Ultrasound	anatomical or tracer	0.01–0.1	mm to cm	10^−12^ M	[123,124]
SPECT ^1^	tracer	8–10 (clinical) 1–2 (preclinical)	unlimited	10^−10^–10^−11^ M	[123,125]
X-ray CT ^2^	anatomical or tracer	0.5–1 (clinical) 0.05–0.2 (preclinical)	unlimited	not determined	[123,126,127]
PET ^3^	tracer	5–7 (clinical) 1–2 (preclinical)	unlimited	10^−11^–10^−12^ M	[123,125]
MRI ^4^	anatomical or tracer	~1 (clinical) 0.025–0.1 (preclinical)	unlimited	10^−3^–10^−5^ M	[123,128,129]
CRET ^5^	tracer	mm	unlimited	10^−11^–10^−12^ M	[123,125,130]
MPI ^6^	tracer	1 mm	unlimited	10^−9^ M	[131,132]
EPRI ^7^	tracer	µm	nm	10^4^ spins per voxel (~100 zmol)	[133]
MSI ^8^	surface imaging	µm	mm to µm	ppm	[134]
SERS ^9^	surface imaging	mm	~5 mm	10^−12^–10^−15^ M	[123]

^1^ single-photon emission tomography; ^2^ X-ray computed tomography; ^3^ positron emission tomography; ^4^ magnetic resonance imaging, ^5^ Cerenkov radiation energy transfer, ^6^ magnetic particle imaging, ^7^ electron paramagnetic resonance imaging, ^8^ mass spectrometric imaging, ^9^ surface-enhanced Raman spectroscopy. (M = molarity; mol/L).

**Table 3:** Summary of imaging hypoxia with multiple probes and multiple modalities.

Dual- or Multi-Modal Imaging Method	Advantages	Limitations
MRI and mass spectrometric imaging	Surface imaging with high spatial resolution	Invasive
Photoacoustic and ultrasound imaging	Sensitive soft tissue imaging, good depth penetration	photostability of chromophores
MRI and SPECT imaging	Deep tissue imaging, high spatial resolution	Exposure to ionizing radiation
MRI and X-ray CT imaging	Detailed anatomical imaging with high spatial resolution	Exposure to ionizing radiation
MRI and PET imaging	Anatomical and tracer imaging with high spatial resolution, deep tissue imaging	Exposure to ionizing radiation
PET and X-ray CT imaging	Tracer and anatomical imaging, highly sensitive	Exposure to ionizing radiation
SERS and photoacoustic imaging	Surface imaging, high sensitivity	Photostability
MRI, EPRI, and PET imaging	Detailed anatomical and tracer imaging	Exposure to ionizing radiation
X-ray CT, MRI, and photoacoustic imaging	Detailed anatomical and tracer imaging, sensitivity	Exposure to ionizing radiation
Fluorescence, photoacoustic imaging, and MRI	Detailed anatomical and tracer imaging, sensitivity	photostability of chromophores

**Table 4:** Summary of dual-mode hypoxia-responsive probes.

Dual-Mode Imaging Method	Advantages	Limitations
Optical imaging and MRI	sensitivity, depth penetration, and spatial resolution	photostability of chromophores, contrast enhancement can be concentration-dependent
Fluorescence and colorimetric imaging	sensitivity, low cost	photostability of chromophores
SERS and fluorescence imaging	sensitivity, photostability	Invasive
SERS and colorimetric imaging	photostability, low cost	Invasive
Fluorescence and photoacoustic imaging	sensitivity, depth penetration, noninvasive	photostability of chromophores
Photoacoustic imaging and MRI	Noninvasive, sensitivity, resolution, and depth penetration	photostability of chromophores
*T*_1_- and *T*_2_-weighted MRI	Accuracy of information	Difficult designing probes that are both *T*_1_ and *T*_2_ active
^1^H- and ^19^F-MRI	Anatomical and tracer information overcomes concentration dependency of probes	solubility of ^19^F-containing probes, sensitivity of ^1^H-MRI
*T*_1_-weighted MRI and CEST	Anatomical and functional information overcomes concentration dependency of probes	Time-consuming, low sensitivity of ^1^H-MRI

## Data Availability

Not applicable.
